# Oxazolidine-Based Chloro-Bridged Dimeric Copper(II) Complex: A Bifunctional Catalyst for Enzyme-Mimetic Aerobic Oxidations and the Oxygen Evolution Reaction

**DOI:** 10.1021/acsomega.6c02828

**Published:** 2026-07-22

**Authors:** Pravesh Kumar, Vinod J, Tarisha Gupta, Ray J. Butcher, Biswajit Mondal, Ritwik Modak

**Affiliations:** † Manipal Institute of Technology Bengaluru, 76793 Manipal Academy of Higher Education, Manipal 576104, India; ‡ Department of Chemistry, 242275 IIT Gandhinagar, Palaj, Gujarat 382355, India; § Department of Chemistry, Howard University, Washington, District of Columbia 20059, United States

## Abstract

Oxazolidine-based dinuclear Cu­(II) complexes, ***[Cu*** ^***II***^_***2***_ ***(μ***_***2***_ ***-Cl)***_***2***_ ***(Cl)***_***2***_ ***(L***_***1R2**S**/1S2R***_)_***2***_ *]* (**1** *and* **2**), were synthesized using a chiral *N,N*-donor ligand (***L***_***1R2**S**/1S2R***_) and CuCl_2_·2H_2_O. Single-crystal X-ray diffraction (SCXRD) confirmed the formation of a μ_2_-Cl-bridged copper complex. Structural features of the dinuclear complexes have been examined in detail, which provides information on C–H···π and π···π supramolecular interactions. The catalytic performance was evaluated in three complementary transformations: the oxygen evolution reaction (OER), catecholase activity, and phenoxazinone synthase (PHS) activity. Electrochemical investigations provide insights into water oxidation catalysis, while spectrophotometric studies were conducted to evaluate catecholase activity toward 3,5-di-*tert*-butylcatechol (DTBC), and for PHS, *o*-aminophenol (OAPH) was used. Faradaic efficiencies (FEs) were calculated as 86 ± 6% for O_2_ and 76 ± 3% for H_2_ in the case of OER activity, and catalytic efficiencies (*k*_cat_) were 1.9 ± 0.4 × 10^3^ and 5.5 ± 0.6 × 10^3^ h^–1^ for catecholase- and PHS-like activities, respectively. Electrochemical, kinetic, and mechanistic studies (in the case of biomimicking activities) reveal that the ligand environment facilitates the redox cycle of Cu­(I)/Cu­(II) and supports the substrate oxidation pathway. Dinuclear copper complexes resemble the active sites of copper oxidase enzymes, and the development of such systems is important for understanding biological activity and designing oxidation catalysts. These findings provide new insights into the design of multifunctional, earth-abundant catalysts capable of energy conversion and oxidation chemistry at the same time.

## Graphical abstract

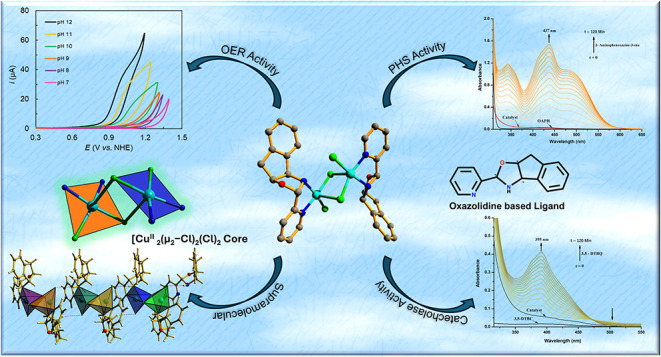

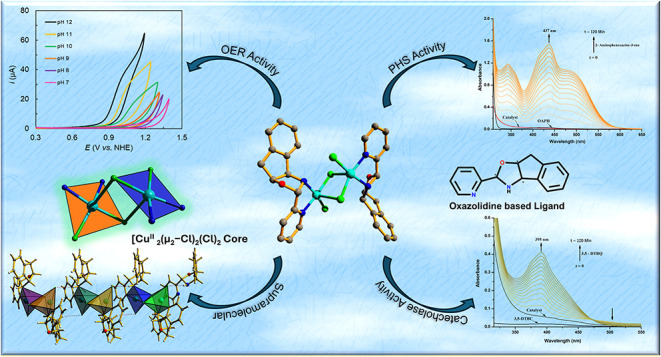

## 1 Introduction

Copper, as one of the most abundant and versatile metals in the earth’s crust, exhibits a variable oxidation state, Cu­(I)/Cu­(II), a feature that underpins its central importance in material science and bioinorganic chemistry.^1^ The ability of copper to reversibly shuttle between oxidation states in its catalytic cycle makes copper an important component of metalloenzymes in natural pathways.^2^ In combination with its natural abundance, low toxicity, and cost-effectiveness compared to other metals, copper has emerged as a sustainable alternative for designing efficient catalytic systems.^3–6^ The versatility of copper-based complexes is not only limited to homogeneous and heterogeneous catalysis but it has also attracted significant attention for their diverse functional applications, ranging from magnetism to antimicrobial applications, anticancer drugs, luminescent probes for bioimaging, and environmental applications.^7–16^ Among these, the catalytic applications are especially influenced by ligand design, as the ligand framework determines the coordination geometry, electronic structure, redox potential, and stability of the metal center. Oxazolidine, a five-membered heterocycle with substantial coordination versatility and tunability when appended with pyridine-type donor groups, becomes a promising *N,N*-donor ligand capable of stabilizing transition-metal centers across multiple oxidation states.^17–20^ The rigid geometry of the ligand is also important for controlling the geometry and reactivity of the metal complexes.^21–24^ This electronic property has affected catalytic performance, as the ligand’s electron-donor capacity directly influences the metal center’s reactivity, substrate binding, and activation process.^25–28^

The oxygen evolution reaction (OER) is acknowledged as the most kinetically demanding half-reaction in water electrolysis, because of its intricate four-electron transfer mechanism and slow reaction dynamics.^29^ Improving OER catalyst performance is, therefore, a critical step toward achieving efficient, low-cost, and large-scale hydrogen production.^30,31^ Oxygen evolution at low overpotentials and hydrogen evolution with high Faradaic efficiency in different media, ranging from acidic to neutral to alkaline, is very important. Although noble-metal oxides such as RuO_2_ and IrO_2_ exhibit excellent OER activity, their scarcity and high cost severely limit their practical deployment, prompting intensive research into earth-abundant alternatives.^32,33^ Copper-based molecular and hybrid catalysts have recently gained attention as promising OER candidates, particularly due to their tunable redox chemistry and ability to access high-valent intermediates under electrochemical conditions.^34–37^ Several studies have demonstrated that appropriate ligand environments can stabilize catalytically active Cu–oxo species, which are proposed to play a key role in O–O bond formation.^38–40^ Dinuclear copper systems are of particular interest as metal–metal cooperativity can lower activation barriers by facilitating multielectron transfer processes and intermediate stabilization.^41–44^ Halide-bridged dinuclear copper complexes, in particular, have been shown to enhance electronic communication between metal centers, thereby improving catalytic robustness and sustaining high current densities during OER.^43^ Despite these advances, rational design strategies linking ligand architecture, nuclearity, and OER performance remain underexplored.

In parallel with electrocatalysis, copper also facilitates aerobic oxidation, effectively mimicking the behavior of natural metalloenzymes (Figure 1). For example, copper plays an important role in the oxidation of catechol to its quinone form, which is important for melanin production, wound healing, and plant defenses.^1,37,45^ Aerobic oxidation can be understood as the capacity of the copper center to efficiently reduce oxygen, with O_2_ serving as the terminal oxidant. Similarly, in phenoxazinone synthase activity, the coupling of two aminophenol groups oxidizes to form a phenoxazinone ring, where the copper center stabilizes the intermediate, facilitates the reaction pathway, and provides a route for the synthesis of phenoxazinone-based drug scaffolds.^46,47^ So, tuning copper-based multifunctional catalysts with halide bridging often serves dual applications, i.e., OER activity and biomimetic models for metalloenzymes such as catechol oxidase (CO) and phenoxazinone synthase (PHS). In such a catalyst, two copper centers facilitate electron transfer, substrate binding, and bond formation and cleavage.^1,37,47^ When combined with an oxazolidine-derived ligand, the structural framework supports selectivity and high reactivity; therefore, the combination of these properties is valuable for constructing a metal-based complex.^48,49^

**Figure 1:**
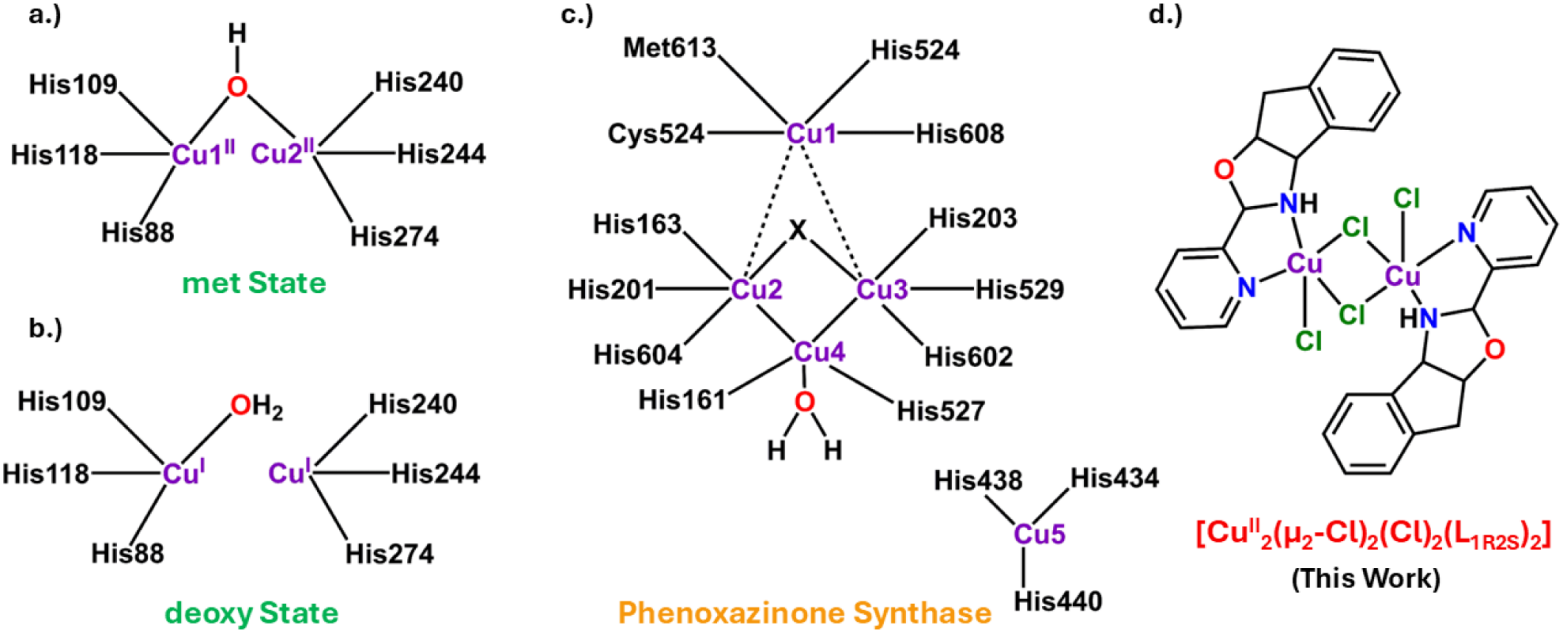
Copper centers, including the coordinating ligands: (a) Met state of catechol oxidase. (b) Deoxy state of catechol oxidase. c) A tetranuclear unit and its surrounding ligands of phenoxazinone (where “X” represents an unidentified bridging ligand such as OH), along with three histidine-coordinated type 2 copper atoms and d) copper complex in this work.

While many mononuclear copper-based complexes with oxazolidine and related motifs have been characterized for their catalytic activity, studies of chloro-bridged dinuclear complexes remain limited.^50^ So, considering the advantages of dinuclear metal complexes and the oxazolidine-based ligand system, we synthesized chloro-bridged dinuclear complexes, **(Cu** ^**II**^_**2**_ **(μ**_**2**_ **-Cl)**_**2**_ **(Cl)**_**2**_ **(L**_**1R2** *S* **/1S2R**_)_**2**_), stabilized by an oxazolidine-pyridine-based ligand (**L**_**1R2** *S* **/1S2R**_), which combines rigidity with tunability and flexibility, where one enantiomer has been previously reported by Wiskur et al.^27^ We determined the structure through single-crystal diffraction studies and other detailed characterization techniques in both the solid state and solution phase. Electrochemical studies reveal that the copper catalyst delivers sustained OER activity over an extended period under milder conditions. As copper-based biomimetic complexes are structurally well-defined systems for probing enzymatic oxidation pathways, the catalytic activities against two common substrates, i.e., 3,5-DTBC (3,5-di-*tert*-butylcatechol) and OAPH (2-aminophenol), were tested. Thus, here we report the bifunctionality of a chloro-bridged binuclear Cu-catalyst **(Cu** ^**II**^_**2**_ **(μ**_**2**_ **-Cl)**_**2**_ **(Cl)**_**2**_ **(L**_**1R2S**_)_**2**_) for OER and substrate oxidation using O_2_ as the terminal oxidant.

## 2 Experimental Section

### 2.1 General Information

2-Pyridinecarboxyaldehyde, 1R2*S*/1S2R amino-2,3-dihydro-1H-inden-2-ol, 3,5-di-*tert*-butylcatechol, and 2-aminophenol were procured from Sigma-Aldrich. CuCl_2_·2H_2_O, sodium sulfate anhydrous, and other reagents were purchased from Loba Chemie and Merck India, respectively. Sodium hydrogen phosphate was purchased from Novus-Chem Strength . Spectroscopy-grade solvent was used for catalytic activity. All other chemicals and reagents were of reagent quality unless specified. ^1^H and ^13^C NMR spectra were recorded on Bruker Biospin in CDCl_3_ with TMS as the internal standard. FTIR measurements were recorded on a Bruker ALPHA II Compact FT-IR Spectrometer in 4000–400 cm^–1^ range. Elemental analyses for C, H, and N were performed on a PerkinElmer 2400 II analyzer. The absorption spectra were recorded on the Shimadzu UV-3600i Plus at room temperature using a quartz cuvette with a 1 cm path length. The circular dichroism (CD) spectra of both the complexes were recorded on a Jasco J-810 spectropolarimeter in methanolic solution. The HRMS data were recorded with a Waters Synapt-G2S ESI-Q-TOF Mass instrument. The PXRD pattern of the complexes was carried out using a Rigaku SmartLab X-ray Diffractometer instrument, with data recorded within a 2θ range of 5° to 50°.

### 2.2 Synthesis and Characterization

#### 2.2.1 Synthesis of Ligands (**L**_**1R2S**_ and **L_1S2R_**)

The Schiff base ligand **L**_**1R2S**_ was prepared by a method adapted from a literature procedure.^27,51^ 2-Pyridinecarboxyaldehyde (0.214 g, 2.0 mmol) was dissolved in methanol (15 mL) in the presence of Na_2_SO_4_. To this, a solution of (1R,2S)-1-amino-2,3-dihydro-1H-inden-2-ol (0.298 g, 2.0 mmol), also dissolved in the same solvent (15 mL), was added dropwise at room temperature with continuous stirring. The reaction mixture was then heated under reflux for 5 h and allowed to cool at room temperature. After filtration, the dark oily liquid was obtained by removing the solvent in a rotary evaporator and then dried overnight in a desiccator over CaCl_2_ and used without further purification. Yield: 82%. C/H/N analysis, calcd for C_15_H_14_N_2_O: C, 75.61; H, 5.92; N, 11.76; found: C, 75.59; H, 5.94; N, 11.75. ^1^H NMR (400 MHz, CDCl_3_, ppm) δ_H_: 8.6 (d, *J* = 4.44 Hz, 1H, HC = N_Py_), 7.6 (dt, *J* = 1.64 Hz, 1H, Ar), 7.5 (t, *J* = 4.88 Hz, 1H, Ar), 7.2–7.3 (m, 5H, Ar), 5.1 (d, *J* = 5.44 Hz, 1H, HC–N), 5.1 (s, 1H, O–CH–N), 4.8–4.9 (m, *J* = 2.56 Hz, 1H, HC–O), 3.2 (m, 3H, H_2_C & NH) (Figure S1 and S2, Supporting Information). ^13^C NMR (400 MHz, CDCl_3_, ppm) δ: 155.83, 149.65, 142.32, 141.20, 136.81, 128.44, 127.28, 126.03, 124.85, 124.02, 123.03, 91.52, 80.37, 68.95, 39.36 (Figure S3, Supporting Information). Selected FT–IR (in cm^–1^) data: *v̅*_(N–H)_ = 3284 (b), *v̅*_(C=N‑py)_ = 1588 (s), *v̅*_(N–C)_ = 1088 (m), *v̅*_(O–C)_ = 1021 (s) (Figure S7, Supporting Information). UV–Vis (in CH_3_OH, c = 1 × 10^–5^ M, λ_max_ [nm] with ε [M^–1^·cm^–1^]): 282 (18200), 271 (65600), 265 (110600), and 259 (121100) (Figure S11, Supporting Information). ESI-HRMS (MeOH, *m*/*z*): Observed: 239.15; Calculated for [**L**_**1R2S**_ **+** H^**+**^]: 239.11 (Figure S15, Supporting Information).

The same methodology was applied for the synthesis of **L**_**1S2R**_, with the exception that the amine utilized was 1S,2R-(1-amino-2-indanol) in place of the previously specified amine. Yield: 83.2%. C/H/N analysis, calcd. for C_15_H_14_N_2_O: C, 75.61; H, 5.92; N, 11.76; found: C, 75.62; H, 5.91; N, 11.77. ^1^H NMR (400 MHz, CDCl_3_, ppm) δ_H_: 8.6 (d, *J* = 3.44 Hz, 1H, HC=N_Py_), 7.6 (dt, *J* = 1.24 Hz, 1H, Ar), 7.5 (t, *J* = 3.12 Hz, 1H, Ar), 7.2–7.3 (m, 5H, Ar), 5.1–5.2 (d, *J* = 4.32 Hz, 1H, HC–N), 5.1 (s, 1H, O–CH–N), 4.9 (m, *J* = 2.12 Hz, 1H, HC–O), 3.2 (m, 3H, H_2_C & NH) (Figure S4 and S5, Supporting Information). ^13^C NMR (400 MHz, CDCl_3_, ppm) δ: 155.84, 149.63, 142.32, 141.19, 136.80, 128.43, 127.27, 126.02, 124.84, 124.01, 123.02, 91.52, 80.37, 68.94, 39.36 (Figure S6, Supporting Information). Selected FT–IR (cm^–1^) data: *v̅*_(N–H)_ = 3284 (b), *v̅*_(C=N‑py)_ = 1593 (s), *v̅*_(N–C)_ = 1094 (m), *v̅*_(O–C)_ = 1016 (s) (Figure S8, Supporting Information). UV–Vis (in CH_3_OH, c = 1 × 10^–5^ M, λ_max_ [nm] with ε [M^–1^·cm^–1^]): 283 (17400), 271 (66900), 265 (112800), and 259 (123700) (Figure S12, Supporting Information). ESI-HRMS (MeOH, *m*/*z*): Observed: 239.15; Calculated for [**L**_**1S2R**_ **+** H^**+**^]: 239.11 (Figure S16, Supporting Information).

#### 2.2.2 General Procedure for the Synthesis of Metal Complexes (**1** and **2**)

The general technique detailed below has been used to synthesize both Schiff base metal complexes.^27^ The solution of Schiff base **L**_**1R2S**_ **/L**_**1S2R**_ (0.238 g, 1.0 mmol) in methanol was stirred, and to this solution, a methanolic solution of CuCl_2_·2H_2_O (0.170 g, 1.0 mmol) was added dropwise with continuous stirring at room temperature. Then, the resulting reaction mixture was stirred for 2 h. Following the filtration of the bluish-green solution, it was left undisturbed, and after a few days, blue-colored X-ray diffraction grade crystals of the complexes deposited at the bottom of the vessel. After the separation, cooled methanol (2 × 3 mL) was employed for washing.

#### 2.2.3 [Cu^II^_2_(μ_2_-Cl)_2_(Cl)_2_(L_1R2S_)_2_] (**1**)

Yield: 76.5%. Selected FT–IR data (cm^–1^): *v̅*_(N–H)_ = 3152 (b), *v̅*_(C=N‑py)_ = 1606 (s), *v̅*_(N–C)_ = 1068 (m), *v̅*_(O–C)_ = 1048 (m) (Figure S9, Supporting Information). C/H/N analysis, calculated for C_30_H_28_Cl_4_Cu_2_N_4_O_2_: C, 48.33; H, 3.79; N, 7.58; found: C, 47.90; H, 3.34; N, 7.75. UV–Vis (in CH_3_OH, c = 1 × 10^–5^ M, λ_max_ [nm] with ε [M^–1^·cm^–1^]): 702 (1000), 303 (62200), 291.5 (81700), and 260 (101200) (Figure S13, Supporting Information).

#### 2.2.4 [Cu^II^_2_(μ_2_-Cl)_2_(Cl)_2_(L_1S2R_)_2_] (**2**)

Yield: 75%. C/H/N analysis, calculated for C_30_H_28_Cl_4_Cu_2_N_4_O_2_: C, 48.33; H, 3.79; N, 7.58; found: C, 48.19; H, 3.50; N, 7.48. Selected FT–IR data (cm^–1^): *v̅*_(N–H)_ = 3152 (b), *v̅*_(C=N‑py)_ = 1605 (s), *v̅*_(N–C)_ = 1068 (m), *v̅*_(O–C)_ = 1045 (m) (Figure S10, Supporting Information). UV–Vis (in CH_3_OH, c = 1 × 10^–5^ M, λ_max_ [nm] with ε [M^–1^·cm^–1^]): 703 (1000), 304 (61100), 291 (83850), and 260 (106550) (Figure S14, Supporting Information).

### 2.3 Single-Crystal X-ray Crystallography

Suitable plate-shaped single diffraction-quality crystals of complexes **1** and **2** for crystallographic analysis were selected under a microscope. The data for the analysis of a single crystal were obtained at 100 K using a Rigaku (XtaLAB Synergy-DW) with a CuKα radiation source and processed using CrysAlisPro.^52^ The structures were solved by SHELXL-2019/2 (Sheldrick, 2019) and refined by using the full-matrix least-squares method on F² with anisotropic parameters for all non-hydrogen atoms.^53^ The crystallographic figures were generated using Diamond 3.0 and Mercury 3.8 software.^54,55^ The crystallographic and important geometrical parameters of the clusters are shown in Tables S1 and S2, Supporting Information. CCDC: 2502869 and 2502870 are included in the supplementary crystallographic data for this paper. These data can be obtained free of charge from the Cambridge Crystallographic Data Centre via www.ccdc.cam.ac.uk/data_request/cif.

### 2.4 Kinetic Studies (Experimental) Details

The catecholase- and phenoxazinone (PHS)-like activities of the complex were studied using a Shimadzu UV 1900 I spectrophotometer with 3,5-di-*tert*-butylcatechol and 2-aminophenol (ortho-aminophenol) as model substrates, respectively. The activity of catecholase was examined by observing the quinone band at 400 nm and for PHS at 435 nm in MeOH as a medium at room temperature over time. For the determination of intermediates during the catalytic activity, electrospray ionization high-resolution mass spectra (ESI-HRMS) were obtained with a Waters XEVO G3-QTOF-HRMS instrument operating in positive ion mode.

### 2.5 Electrocatalytic OER (Experimental) Details

All electrochemical measurements were carried out using a three-electrode system with a *CHI 7044E* potentiostat. A glassy carbon electrode (GCE) with a geometrical area of 0.07 cm^2^ served as the working electrode, with a platinum wire as the counter electrode and an Ag/AgCl (sat. KCl) electrode as the reference. The cyclic voltammogram (CV) was taken in methanol with 0.1 M tetrabutylammonium hexafluorophosphate (TBAPF_6_) as a supporting electrolyte. For electrochemical oxygen evolution reaction (OER) studies, 0.1 M phosphate buffer solution (PBS) with adjusted pH was used with 0.1 M Na_2_SO_4_ as a supporting electrolyte.

Constant potential electrolysis was performed at 1.80 V (vs RHE) at room temperature under constant stirring in an undivided cell with a 1 × 1 cm^2^ carbon cloth as the working electrode, Pt as the counter electrode, and Ag/AgCl as the reference. The amounts of produced hydrogen and oxygen in the reactor headspace were analyzed using a gas chromatograph (CIC-Dhruva) equipped with a thermal conductivity detector (TCD).^35,56^ The calibration of the instrument was performed using a commercial gas mixture cylinder (with known concentrations) under similar reaction conditions. The molar amounts were calculated through the area–ppm–mole conversion using the calibration data according to the literature.^36,57^

## 3 Results and Discussion

### 3.1 Syntheses and General Characterization

The reaction of enantiomerically pure 1-amino-2-indanol with 2-pyridinecarboxaldehyde in a 1:1 molar ratio in methanol under refluxing conditions gave the 1,3-oxazolidine-based ligand, **L**_**1R2** *S* **/1S2R**_ (Scheme 1). While primary amines reacting with aldehydes generally form a Schiff base. The presence of a suitably positioned hydroxyl group in amines with an electron-withdrawing group attached to the aldehyde favors the formation of the oxazolidine ring instead of the simple Schiff base. However, the synthesis of Schiff base compounds is facilitated by electron-donating groups such as phenol derivatives. Therefore, by introducing an electron-withdrawing group like pyridine in the aldehyde, it is feasible to synthesize 1,3-oxazolidine. Furthermore, the presence of drying agents like sodium sulfate for dehydration and high temperatures under reflux can promote the removal of water from the hydroxyl group rather than the NH group of the hemiaminal intermediate, which eventually favors the synthesis of 1,3-oxazolidine (Scheme 1).^25–27,49,50^ Elemental analyses, NMR, FT-IR, UV–vis, and HRMS spectroscopy, as shown in Figure S1–S16 (see Supporting Information), confirmed the formation of both the 1,3-oxazolidine-based ligand (**L**_**1R2** *S* **/1S2R**_). The addition of CuCl_2_ **·**2H_2_O in a methanolic solution of the ligand (**L**_**1R2** *S* **/1S2R**_) in a 1:1 ratio, followed by crystallization through slow evaporation, results in the formation of dimeric complexes **1** and **2** (as shown in Scheme 1).

**Scheme 1:**
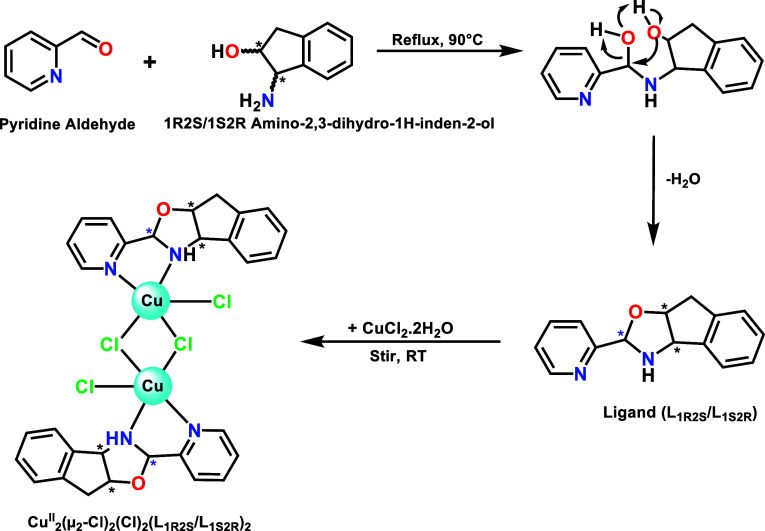
Mechanism and Synthetic Outline of the Ligand **L**_**1R2S**_ **/L**_**1S2R**_ and Complexes **1** and **2**

In the FT-IR spectrum of **L**_**1R2** *S* **/1S2R**_, the broad absorption band at 3284 cm^–1^ can be assigned to the N–H group, and the band broadening can be attributed to hydrogen bond interactions. The characteristic signals were observed at 1021–1088 cm^–1^ for the vibration of C–O and C–N bonds of the oxazolidine ring. In the FT-IR spectrum of **L1R2** *S* **_/1S2R_,** the absence of azomethine *v̅* (C=N) vibration, which is usually observed at ∼1630 cm^–1^, indicates that the product of the reaction between amine and aldehyde is not a Schiff base compound.^51^ In **L**_**1R2** *S* **/1S2R**_, the pyridine *v̅,* (C= N_py_) stretching vibration appears in the characteristic 1588–1594 cm^–1^ IR region. In complexes **1** and **2**, the *v̅* (C=N_Py_) stretching vibration appears with a strong upshift to ∼1605 cm^–1^, indicating coordination to the metal center due to the reduction resonance contribution of the nitrogen lone pair into the ring, C=N_Py_ bond becomes stronger with more double-bond character.^28^ ^1^H NMR spectra analysis of the synthesized ligand in CDCl_3_ at room temperature indicates the formation of a 1,3-oxazolidine ring with characteristic O–CH–N and HC=N_Py_ protons, as mentioned in the Experimental section (as shown in Figure S1 and S4, Supporting Information). The reaction proceeds through the nucleophilic attack on the carbonyl carbon of 2-pyridinecarboxaldehyde by the −NH_2_ group of the enantio-pure *cis*-1-amino-2-indanol leading to the formation of a hemiaminal intermediate, followed by a condensation step to form the oxazolidine ring with a mixture of two possible diastereomers (as shown in Scheme 1). The critical analysis of each proton NMR spectrum reveals the presence of both diastereomers in a ratio of 1:6 for **L**_**1R2S**_ and 1:5.6 for **L**_**1S2R**_ (see Figure S2 and S5, Supporting Information). After analyzing the ^1^³C NMR, 15 distinct carbon signals, which are consistent with its structure, along with 15 minor peaks, indicate the presence of minor diastereomeric species in both ligands (see Figure S3 and S6, Supporting Information). The high-resolution mass spectra of **L**_**1R2** *S* **/1S2R**_ were taken in methanol. The observed peak of *m*/*z* at 239.15 suggests the successful formation of **L**_**1R2** *S* **/1S2R**_ (see Figure S15 and S16, Supporting Information).

The absorption bands in the electronic spectra of both the ligands and complexes, taken in methanol solution (as shown in Figure S11–S14, Supporting Information), exhibit very similar spectral patterns for both the complexes with a five-coordinated Cu^II^ ion having a distorted square pyramidal geometry. The spectra have a broad band centered at ∼700 nm due to *d*_*xz*_,*d_yz_→ d*_*x2–y2*_ crystal field transition. The broad band centered at ∼442 nm is due to the ligand-to-metal charge transfer (*LMCT*) transitions.^58^ The other higher-energy transitions at about 259 and 292 nm are due to intraligand transitions. Again, no changes are observed when the solution of the metal complexes is monitored spectroscopically for a long time, indicating that it retains its dimeric nature. In the UV–vis spectra of both the ligands, the absence of a strong absorption band for π → π* transition further supports the formation of a 1,3-oxazolidine ring instead of an imine bond (Figure S11 and S12, Supporting Information).

The chiral copper complexes **1** and **2**, formed by the oxazolidine ligands, i.e., **L**_**1R2** *S* **/1S2R**_ are characterized with Circular Dichroism (CD) spectroscopy in methanol (2.68 × 10^–4^ M) to confirm their enantiomeric purity. CD under identical conditions at 20 °C shows a Cotton effect for each enantiomer, with opposite effects with respect to each other.^59,60^ The CD spectra confirm that complexes **1** and **2** are mirror images, where complex **1** shows a positive Cotton effect at lower wavelengths and a negative effect at higher wavelengths, and vice versa for complex **2** (as shown in Figure 2). The Cotton effect of the complexes can be valuable for further studies in asymmetric catalysis, CPL studies, and the development of optoelectronic materials.

**Figure 2:**
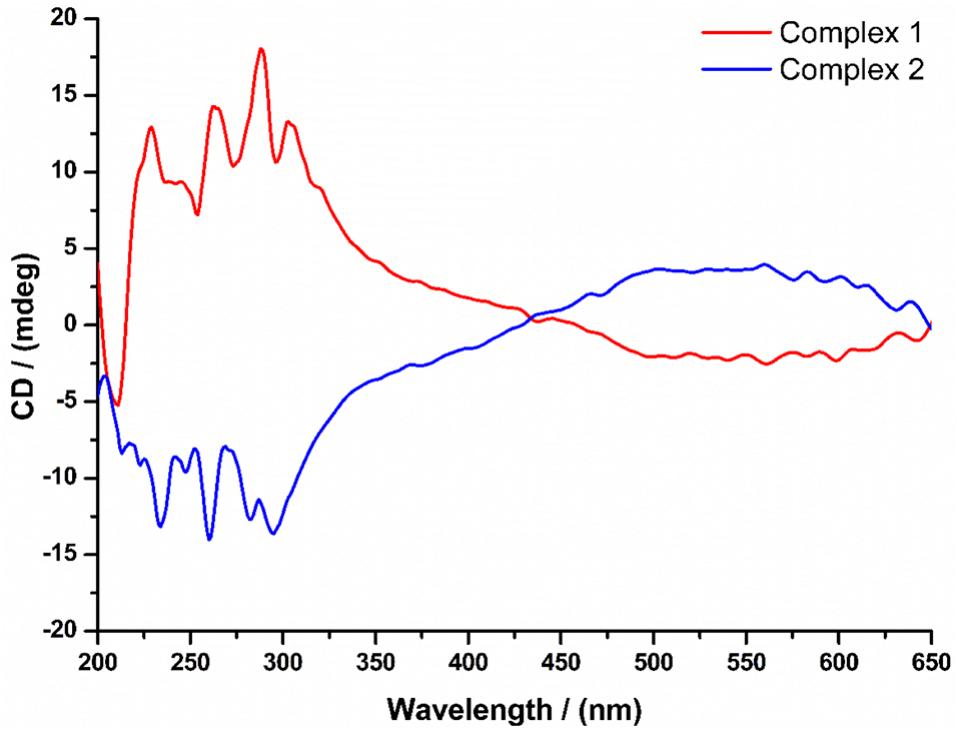
CD spectra of the methanolic solution of complex **1** (red) and **complex 2** (blue).

As shown in Figures S17 and S18 (in Supporting Information), the high-resolution mass spectra of complex **1**, taken as a representative example in methanol, confirm the preservation of the nuclear integrity and demonstrate the stability of the complex in solution. The observed *m*/*z* 743.43, 727.46, and 823.51 correspond to [Cu_2_(Cl_4_)­(L_2_) + H^+^], [Cu_2_(Cl_3_)­(L_2_) + H_2_O]^+^, and [Cu_2_(Cl_3_)­(L_2_) + 3CH_3_OH + H_2_O]^+^ (where L = **L**_**1R2S**_), respectively. Powder X-ray diffraction was performed on powdered polycrystalline complexes **1** and **2** to confirm the sample purity. It is observed that the simulated PXRD pattern correlates well with the experimental spectra, confirming the phase purity of both complexes at room temperature (see Figures S19 and S20, Supporting Information).

### 3.2 Description of the Solid-State Structures

Single-crystal X-ray diffraction reveals that complexes **1** and **2** crystallize in the monoclinic system, and according to the observed systematic extinctions, their structures were solved in the *P2*_1_ space group with *Z* = 2 as enantiomerically pure, blue, plate-like crystals with a single diastereomeric form in the crystal packing. As both complexes are structurally similar, the enantiomeric relations at the oxazolidine chiral carbon centers **C**_**6** *A* **/6B**_, **C**_**7** *A* **/7B**_, and **C**_**15** *A* **/15B**_ are (***S,S,R***) and (***R,R,S***) for complexes **1** and **2**, respectively. The crystal structure of **1** is described in detail as a representative example.

Complexation of Cu­(II) with **L**_**1R2S**_ leads to the formation of a five-coordinated dinuclear Cu­(II) complex, as depicted in Figure 3a. Both ligands in the asymmetric unit share the same diastereomeric form, with minor conformational variations. Their stereocenters, C_7*A*/7B_ and C_15*A*/15B_ retain identical chiral configurations, S and R, respectively. The substituents at positions 2 and 4 of the oxazolidine rings (C_6*A*/6B_ and C_15*A*/15A_) adopt an antirelationship, positioning them on opposite sides after the formation of the oxazolidine ring. Each of the ligands bears three potential donation sites, out of which only the pyridine and oxazolidine nitrogen atoms take part in chelation. The asymmetric unit of the complex shows that Cu­(II) coordinates to the N_1*A*/1B_ of the pyridine ring and N_2*A*/2B_ of the oxazolidine ring, along with three chloride anions in a *cis-*arrangement. Two independent monomeric units, each coordinated to Copper­(II), dimerize via μ^2^-chloride (Cl_2A_ and Cl_2B_) bridging anions, forming a Cu_2_Cl_2_ rectangular core.

**Figure 3:**
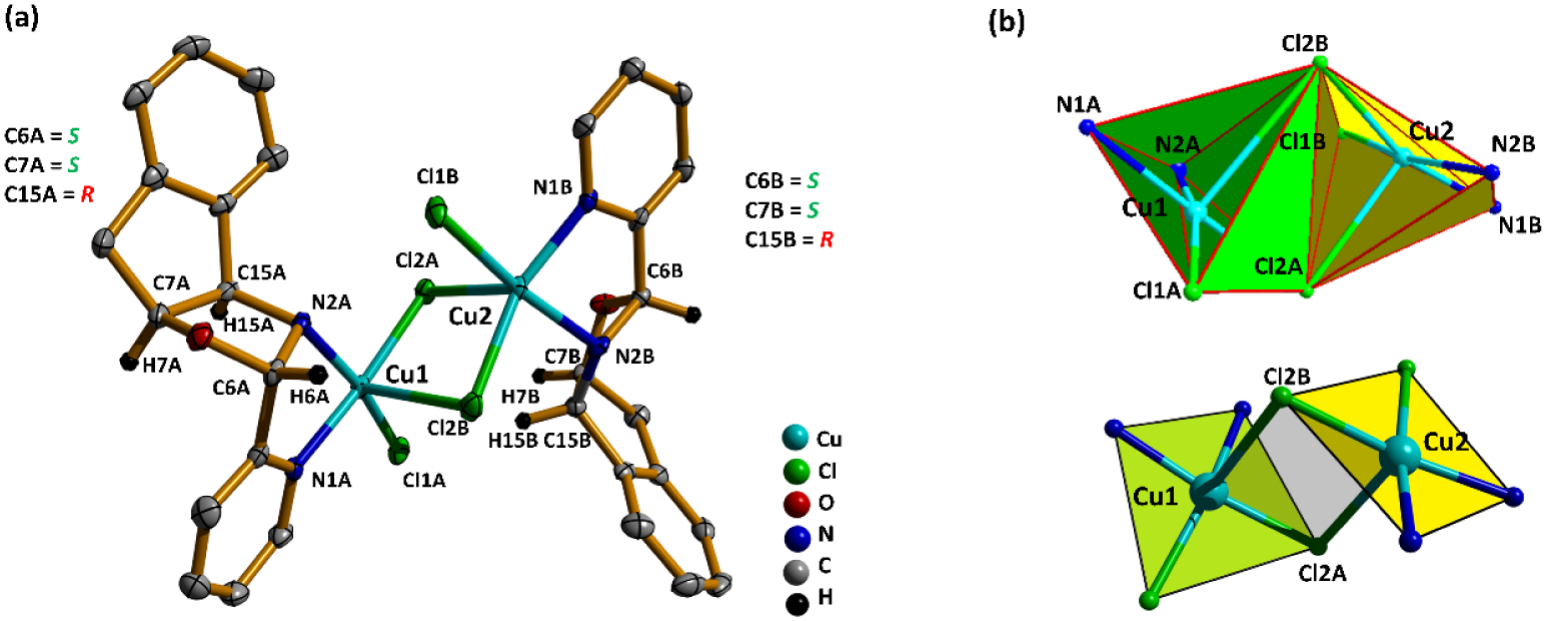
(a) Crystal structure of complex **1**. All the hydrogen atoms, except those attached to chiral carbons, are removed for clarity. (b) View showing the arrangement (above) and base-to-apex edge sharing (below) of distorted square pyramidal geometry around Cu^II^ centers’ polyhedra in complex **1**.

The average bond lengths between the metal and donor atoms are 2.0235 Å for C*u*_1/2_–N_1*A*/1B_ of the pyridine ring, 2.0285 Å for C*u*_1/2_–N_2*A*/2B_ of the oxazolidine ring, 2.262 Å for C*u*_1/2_–Cl_1*A*/1B_ and in the Cu_2_(μ^2^-Cl)_2_ rectangular core, 2.163 Å for C*u*_1/2_–Cl_2*A*/2B_ and 2.470 Å for axial C*u*_1/2_–Cl_2*B*/2A_. By using the formula τ = (β – α)/60° for the calculation of the Addison Distortion Index, the values obtained are 0.0705 for Cu_1_ and 0.132 for Cu_2_, which are best ascribed as close to penta-coordinated square-pyramidal geometry.^61^ In the chromophore group CuN_2_Cl_3_, each copper (i.e., C*u*_1/2_) with N_1*A*/1B_, N_2*A*/2B_, Cl_1*A*/1B_, Cl_2*A*/2B_ forms the base, and the longer Cl_2B_ for Cu_1_ and Cl_2A_ for Cu_2_ holds the apical position, giving the base-to-apex edge-sharing polyhedral with a 20.01° basal plane angle (Figure 3b). The copper centers, Cu_1_ and Cu_2_, are lifted toward the apical atoms Cl_2B_ and Cl_2A_ by 0.096 and 0.232 Å, respectively. Continuous SHAPE measurement analyses reveal that the actual polyhedral shape for copper centers (Cu_1_ and Cu_2_) appears to be distorted square-pyramidal geometry (SPY-5) with considerable deviations of 2.286 and 1.067, respectively, from the ideal *C*_*4v*_ symmetry (see Table S6, Supporting Information).^62^ The greater distortion in the Cu_1_ than Cu_2_ centered square-pyramidal geometry is due to a more elongated axial Cu–Cl bond. The average bridging angle of Cu_1_–Cl_2*A*/2B_–Cu_2_ [89.14°] is close to the average 89.40° angle found in the literature, while the chelating Cl_2A_–C*u*_1/2_–Cl_2B_ angle takes the value of 90.22°.^63^ This large deviation in the average bond angle of N_1*A*/1B_–C*u*_1/2_–N_2*A*/2B_ (81.04°) from the optimal 90° is due to the limited flexibility between the two coordinating nitrogen of the ligand.

The critical analysis of the crystal structure of complex **1** reveals that the overall 3D crystal packing is stabilized by networks of strong hydrogen-bonding interactions together with multiple weak noncovalent supramolecular interactions. The individual 1D chain forms along the crystallographic *a* axis due to the presence of intermolecular hydrogen bonding (N_2B_–H_1_···**Cl**_**1A**_^**b**^ = 2.50 Å and C_15A_–H_15A_···**Cl**_**1B**_^**a**^ = 2.72 Å) between the adjacent dimeric units (see Figure 4, Table S3 in Supporting Information). These 1D chains are interthreaded with each other through weak intermolecular C_2B_–H_2B_···**O**_**1B**_^**e**^ (2.50 Å) hydrogen bonds, C–H···π (C_2A_–H_2A_···**Cg11** ^**d**^ = 2.79 Å), as well as π···π slipped stacking interactions of the arene vs pyridine rings with a slip angle of 25.4° and a vertical displacement between the ring centroids of 3.951 Å (centroid separation within **Cg9···Cg10** ^**c**^ = 4.01 Å) (as shown in Figure 5, Table S4 and S5 in Supporting Information), eventually giving the overall 3D crystal packing.

**Figure 4:**
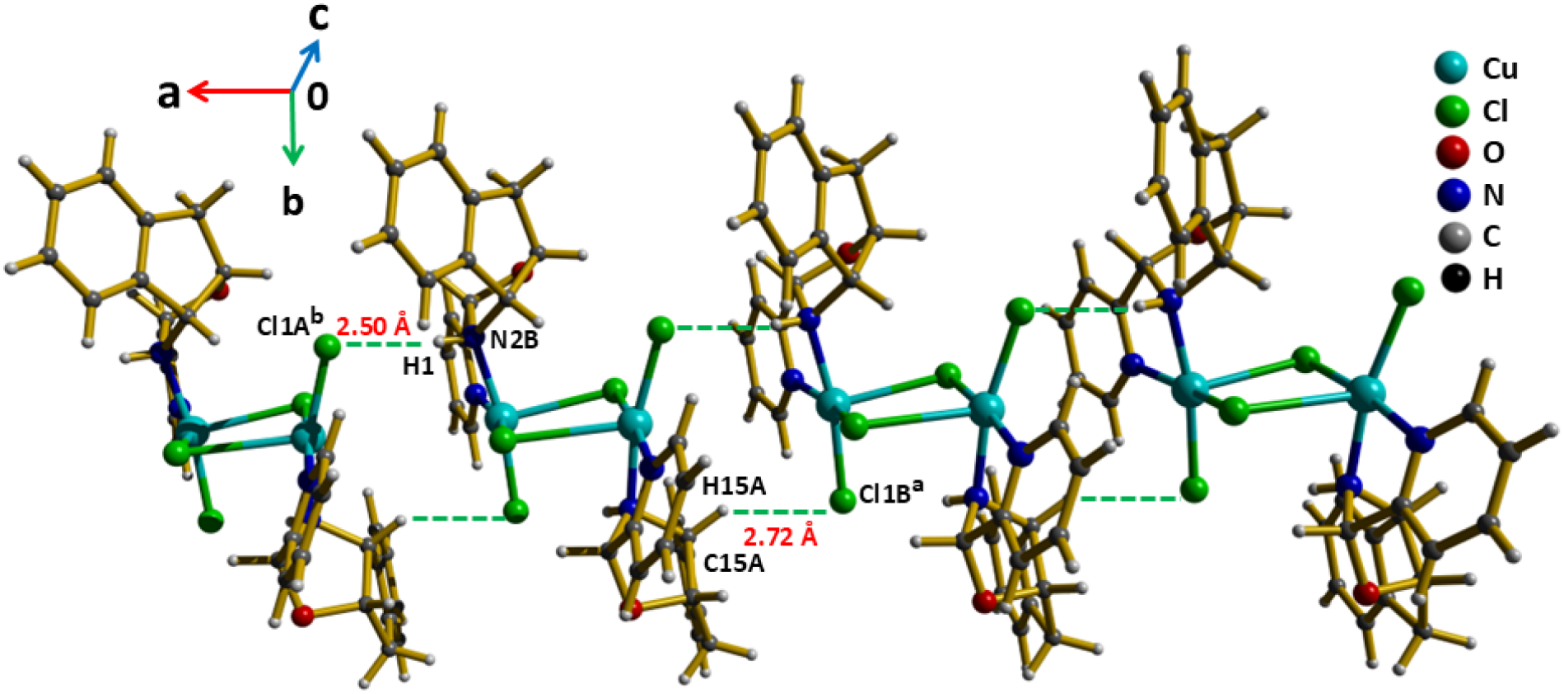
Formation of a one-dimensional polymer through intermolecular N–H···Cl and C–H···Cl interactions in complex **1,** mediated by axial chlorides. Symmetry transformations used to generate equivalent atoms: (^a^) = −1 + x, y, z and (^b^) = 1 + x, y, z.

**Figure 5:**
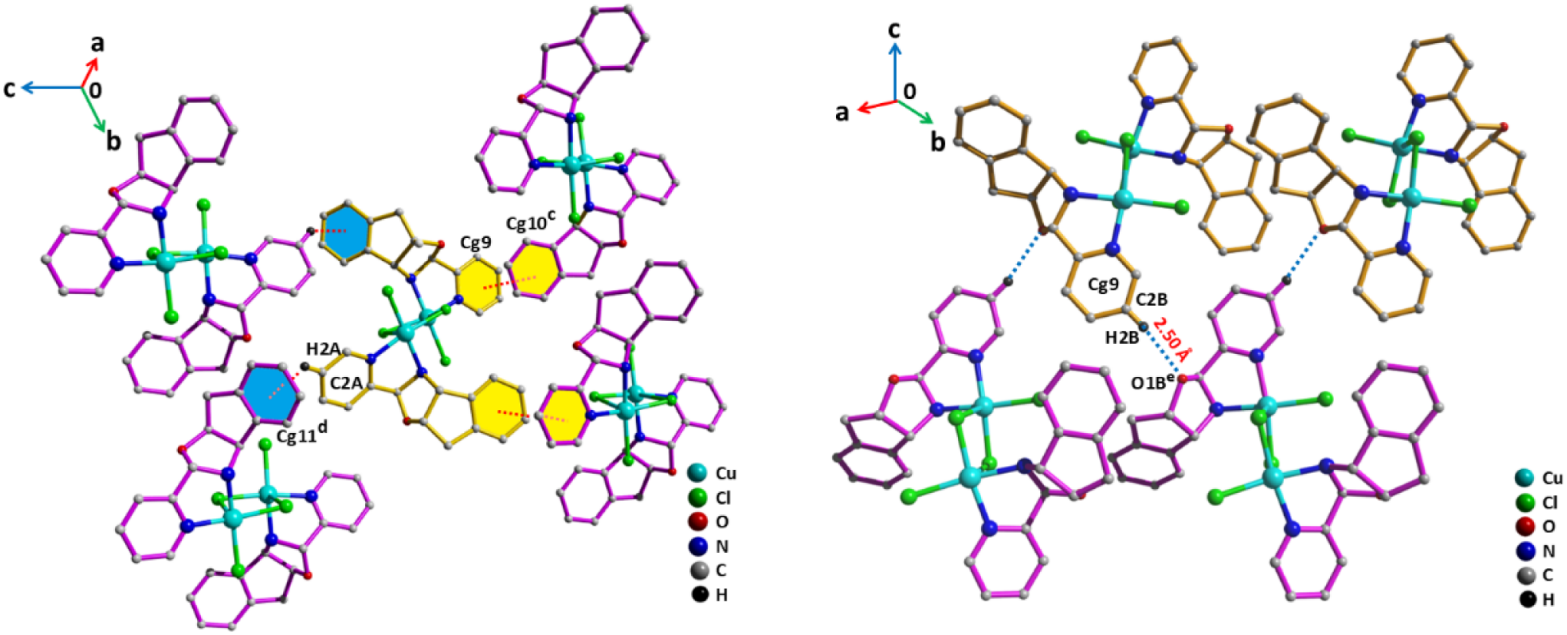
Formation of crystal packing through intermolecular (a) π···π stacking (shown as dotted red line) and C–H···π interactions and (b) hydrogen-bonding interactions (shown as dotted blue lines). Symmetry transformations for equivalent atoms: (c) = 1 – x, −1/2 + y, −z; (d) = 1 – x, 1/2 + y, 1 – z; and (e) = 2 – x, 1/2 + y, −z.

### 3.3 Catalytic Activity and Kinetic Studies of Complex 1

#### 3.3.1 Electrochemical Oxygen Evolution Reaction (OER) Activity

The cyclic voltammogram (CV) of 1 mM complex **1** (in Figure 6) at a scan rate of 100 mV s^–1^ in methanol (with 0.1 M supporting electrolyte) under a nitrogen atmosphere shows two redox couples, consistent with the previous literature.^64,65^ The *E*_1/2_ corresponding to couples 1 and 2 lies at −0.26 V and −0.01 V vs Fc^+/0^, respectively. The nature of both redox couples was checked individually using scan-rate dependence analysis (in Figure S21 and S22, Supporting Information). The peak-to-peak separation (Δ*E*_p_) between the anodic and the cathodic peak currents is found to be 0.16 and 0.07 V for couples 1 and 2, respectively. The redox events 1 and 2 are quasi-reversible.^64^ However, the linear dependence of the peak current on the square root of the scan rate indicates that both processes are diffusion-controlled, with the electrochemically active species freely diffusing in the solution.^66–69^

**Figure 6:**
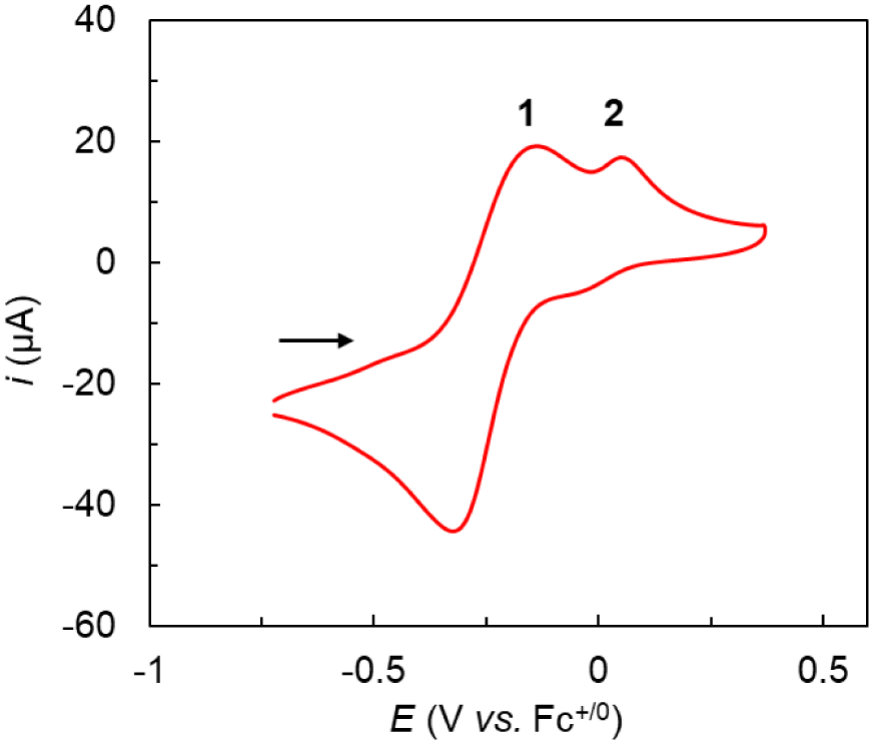
Cyclic voltammogram (CV) of complex **1** (1 mM) in methanol at a scan rate of 100 mV s^–1^. Experimental conditions: 100 mM TBAF as supporting electrolyte in methanol; glassy carbon, Pt wire, and Ag/AgCl were used as the working, counter, and reference electrodes, respectively; 298 K. The potential is adjusted with respect to Fc^+/0^.

The electrochemical OER activity of complex **1** was subsequently assessed under different pH conditions ranging from pH 12 to pH 9 in a fully aqueous medium using 0.1 M phosphate buffer solution (PBS) with 0.1 M Na_2_SO_4_ as the supporting electrolyte, as shown in Figure 7, adjusted to different pHs. Before electrochemical analysis, the UV–visible spectra of complex **1** were recorded for all the pHs. The spectra show slight variations in the characteristic color of the catalyst for pH ranges 7–9 and 10–12, as shown in Figure S23 (Supporting Information). For electrochemical OER in the presence of complex **1** at pH 12, a notable oxidation wave was observed, with the onset appearing around 1.56 V vs RHE, corresponding to an onset overpotential of 330 mV, as depicted in Figure 7a. At pH 12, the current achieved a significantly enhanced value of 65 μA, surpassing the blank OER (see Figure S24a, Supporting Information). A similar observation was encountered for the pH 9 OER activity while performing the control experiment comparing the catalytic and the blank systems (as shown in Figure S24b, Supporting Information). Complex **1** displays a pH-dependent OER activity, with a slope of 96 pH mV dec^–1^ in the pH range of 7–12 (Figure 7b), suggesting a proton-coupled electron transfer process.^70^ A control experiment using CuCl_2_ (1 mM) under identical conditions (pH 12) exhibited negligible anodic current compared to complex **1** (Figure S25, Supporting Information), confirming that the observed OER activity originates from the molecular catalyst rather than the Cu­(II) precursor and highlighting the superior catalytic efficiency of complex **1**. To rule out any heterogeneous contributions, a rinse test for OER was performed at pH 12. The catalytic current disappeared and reached the level of blank OER after rinsing the electrode with water (without mechanical cleaning), ensuring the true homogeneity of the system (Figure S26, Supporting Information).

**Figure 7:**
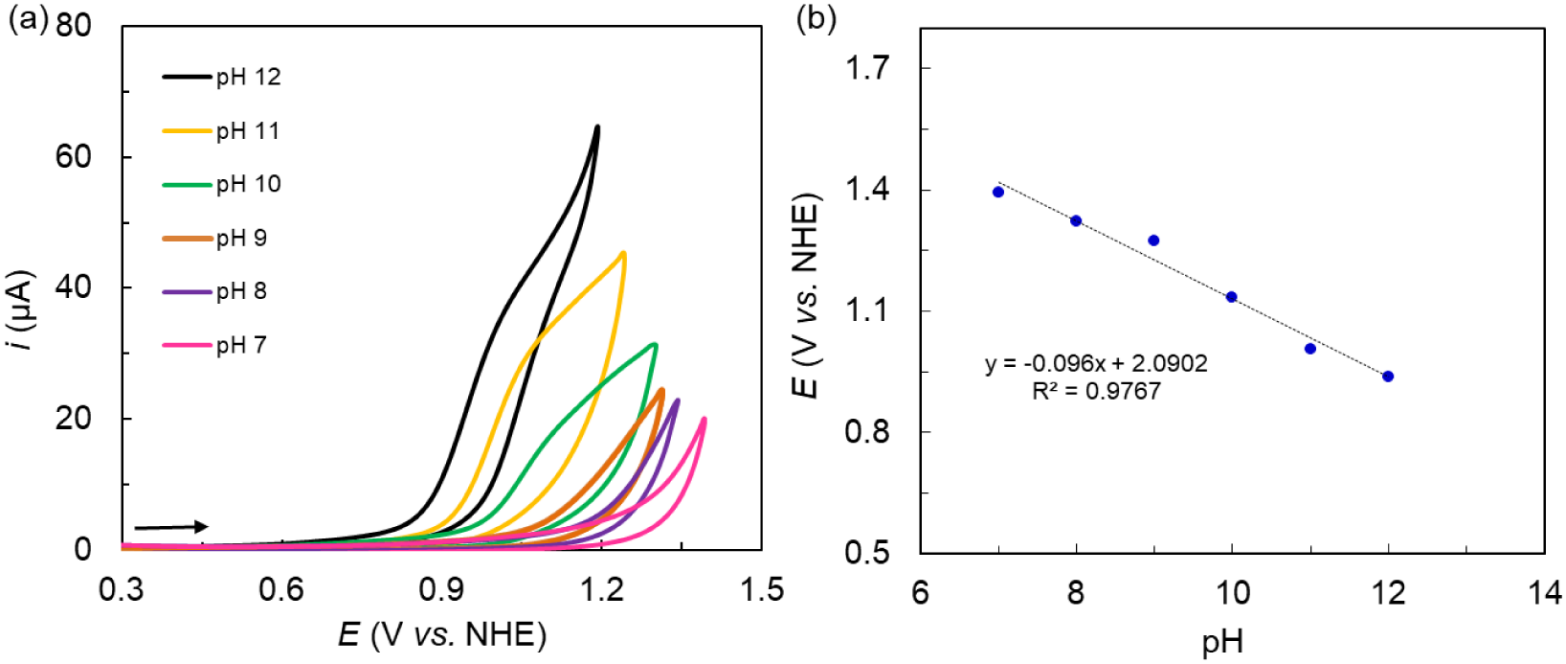
(a) Cyclic voltammograms of OER using complex **1** (1 mM) at different pHs ranging from 12 to 7. (b) Corresponding Pourbaix diagram (*E vs* pH plot). Experimental conditions: 0.1 M PBS with 0.1 M Na_2_SO_4_ as supporting electrolyte (with adjusted pH); glassy carbon, Pt wire, and Ag/AgCl were used as the working, counter, and reference electrodes, respectively; 298 K. The potential is adjusted with respect to NHE.

The controlled-potential electrolysis (CPE) experiment, using a large surface area carbon cloth electrode (1 cm^2^) as the working electrode, was carried out to explore the long-term stability of the catalyst and product characterization under test conditions. Figure 8a shows the catalytic current and total charge passed (10 C) during the CPE experiment in pH 12 at 1.8 V (vs RHE) over 2 h. After electrolysis, the gaseous products formed at the anode (O_2_) and the cathode (H_2_).

**Figure 8:**
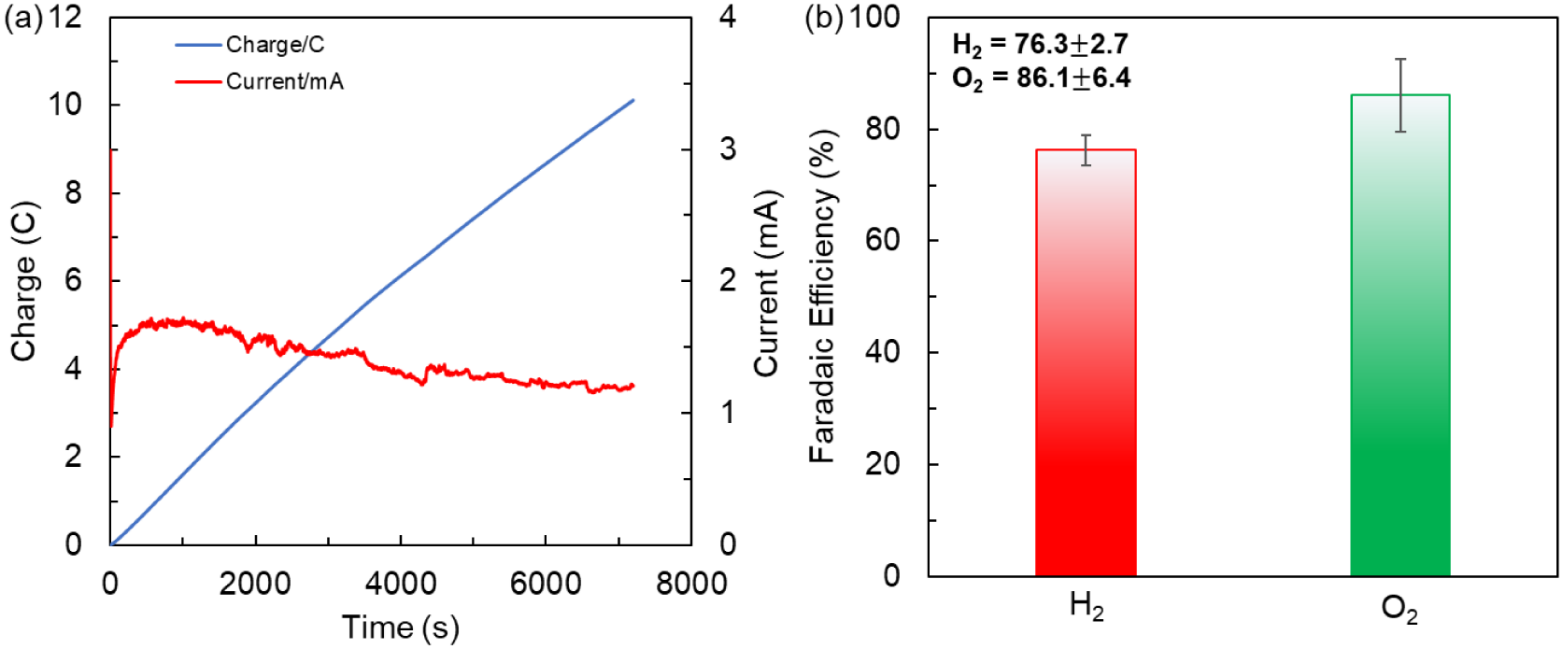
Controlled potential electrolysis (CPE) profile at 1.8 V (vs RHE) for 2 h in pH 12; (b) corresponding Faradaic efficiencies (FEs) of evolved hydrogen and oxygen. Experimental conditions: 0.1 M PBS with 0.1 M Na_2_SO_4_ as supporting electrolyte (adjusted to pH 12); 1 × 1 cm^2^ carbon cloth, Pt wire, and Ag/AgCl were used as the working, counter, and reference electrodes, respectively; 298 K.

(H_2_) were quantified using gas chromatography (GC). According to the area under the GC signal, the moles of O_2_ and H_2_ were calculated, and using the total consumed charge (10 C) during the CPE experiment, the Faradaic efficiencies (FEs) were calculated as 86 ± 6% for O_2_ and 76 ± 3% for H_2_ (Figure 8b). The pre- and postelectrolysis analysis of the electrolyte using UV–vis spectra (Figure S27, Supporting Information) displays a slight decrease in the intensity of the UV absorption after the CPE experiment, suggesting catalyst decomposition.

The literature comparison (Table S7, Supporting Information) indicates that homogeneous dinuclear Cu-based OER catalysts typically operate at relatively high overpotentials (∼630–830 mV, in near-neutral medium) with Faradaic efficiencies (FEs) in the range of ∼90–98%. In contrast, our catalyst [Cu^II^_2_(μ_2_-Cl)_2_(Cl)_2_(L_1R2S_)_2_] exhibits a significantly lower overpotential (330 mV) under alkaline conditions with an FE for O_2_ (86.1 ± 6.4%), placing it among the most efficient homogeneous Cu-based systems and approaching heterogeneous benchmarks. Overall, the combination of substantially reduced overpotential and competitive FE highlights a favorable activity-efficiency balance, supporting the superior catalytic performance of our system relative to previously reported homogeneous dinuclear Cu catalysts.

#### 3.3.2 Catechol Oxidase (CO)- and Phenoxazinone Synthase (PHS)- Mimicking Activities

##### 3.3.2.1 Oxidation of 3,5-Di-*tert*-butylcatechol (3,5-DTBC)

The catecholase activity of the synthesized complex **1** was evaluated by studying the oxidation of 3,5-di-*tert*-butylcatechol (3,5-DTBC) to 3,5-di-*tert*-butylquinone (3,5-DTBQ) (see Scheme 2). The bulky butyl group on the tertiary position allows for the easier oxidation process of DTBC. A 1 × 10^–4^ M methanolic solution of complex **1** was mixed with the methanolic solution of substrate 3,5-DTBC, varying its concentration from 1 × 10^–3^ M to 1 × 10^–2^ M, and the reaction was monitored spectrophotometrically between 300 and 550 nm under aerobic conditions at room temperature (25 °C). A progressive increase in absorbance at 395 nm, characteristic of 3,5-DTBQ, confirmed catecholase-like activity, while the decrease in the electronic band at 550 nm suggested the depletion of metal-bound semiquinone species in solution (see Figure 9).^71,72^ An isosbestic band at 475 nm was observed during the oxidation process of 3,5-DTBC, which is considered asan intermediate species.^73,74^ To maintain pseudo-first-order conditions, the substrate concentration was kept at least 10 times higher than the complex **1** concentration. All experiments were performed at room temperature. Kinetic studies were performed by varying substrate concentrations, and absorbance changes were recorded. The slopes were determined from the plot of log [*A*_α_/(*A*_α_ – *A_t_*)] vs *t* (time curve) (Figure 10a).^75^ The reaction exhibited first-order dependence on complex concentration and followed Michaelis–Menten kinetics, with saturation observed at higher 3,5-DTBC concentrations. A Lineweaver–Burk plot (Figure 10b) was used to determine kinetic parameters like *V*_max_, i.e., maximum reaction velocity; *K_m_,* i.e., Michael constant; and *k*_cat_, i.e., turnover number (listed in Table S8, Supporting Information).^76^ The catalytic efficiency, i.e., *k*_cat_ = 1.9 ± 0.4 × 10^3^ h^–1^ showed that complex **1** exhibited moderate catecholase activity compared to some previously recorded values given in Table S8 (Supporting Information).^77–86^

**Scheme 2:**
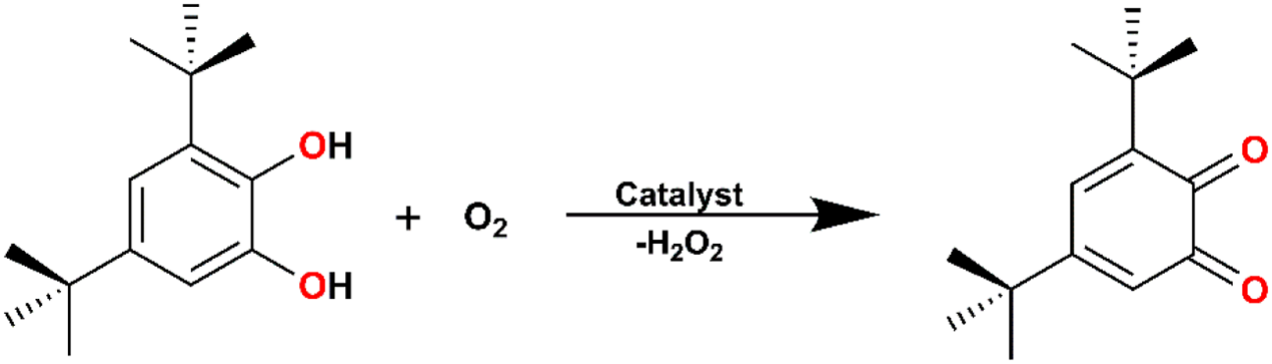
Oxidative Conversion of 3,5-DTBC to 3,5-DTBQ Catalyzed under O_2_ Atmosphere

**Figure 9:**
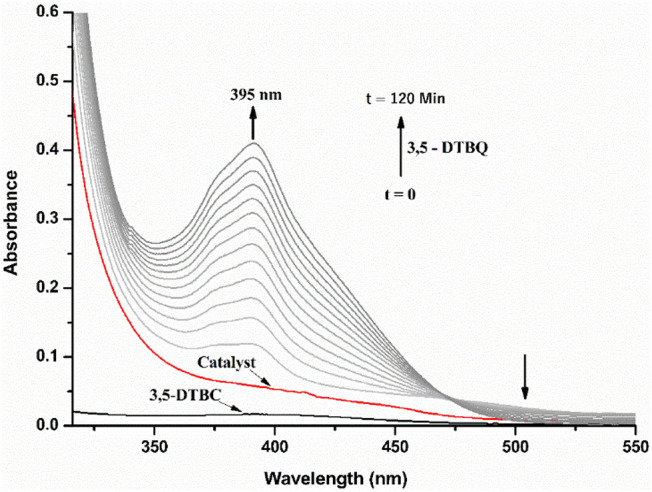
Oxidation of 3,5-DTBC by complex **1** observed in MeOH by UV/vis spectroscopy over time.

**Figure 10:**
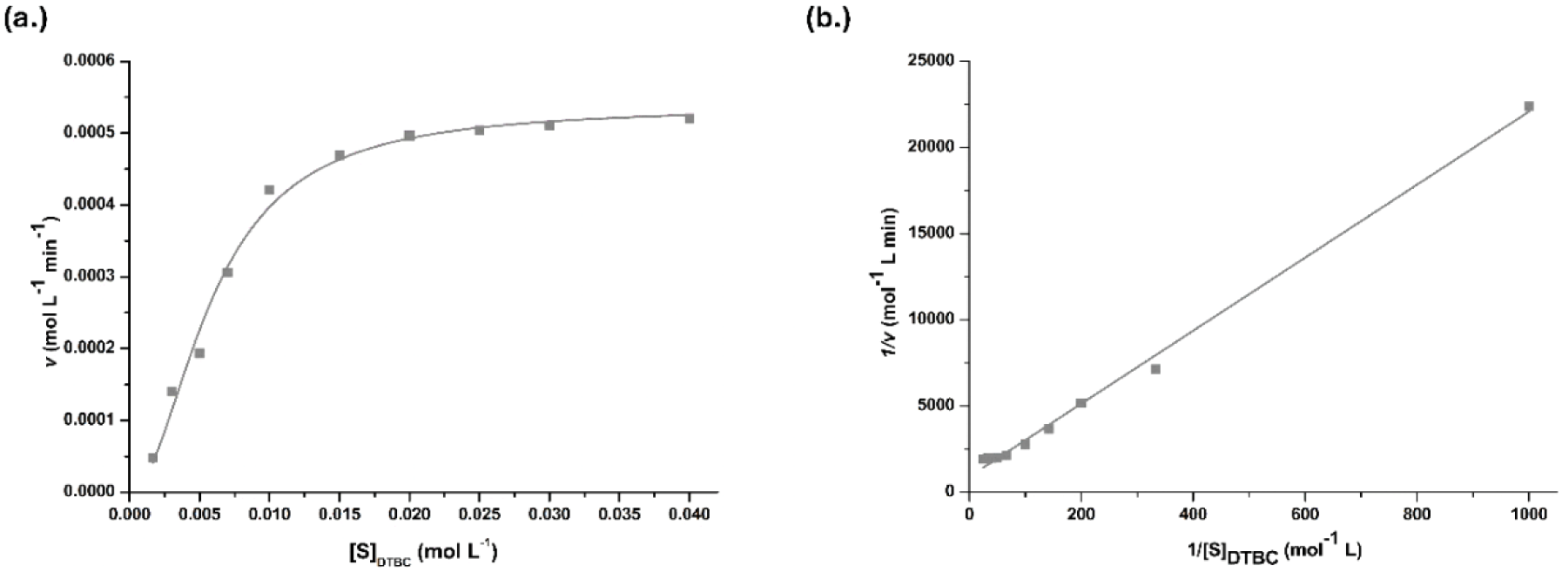
(a) Dependence of the rate of reaction on substrate concentration for complex **1** in methanol for oxidation of catechol. (b) Lineweaver–Burk plot for complex **1** in MeOH medium for oxidation of catechol.

The interaction between the catalyst and the substrate influences the catalytic activity. The *k*_cat_ value increases as the substrate–catalyst interaction increases. To get an insight into the mechanistic cycles, HRMS analysis in positive ion mode was performed on the reaction mixture after mixing the copper catalyst with substrates (3,5-DTBC) in a 1:50 ratio after 10 min in MeOH (see Figure S28, Supporting Information) because it provides important information regarding intermediates and final products formed during the reaction. The base peak at *m*/*z* = 540.20 for catecholase-like activity corresponds to [Cu­(H_2_O)­(L)­(3,5-DTBC)]^+^. Another prominent peak at *m*/*z* = 336, 521.20, and 301.04 can be assigned to [Cu_2_(Cl_2_)­(L_2_)]^2+^, [Cu­(L)­(3,5DTBC)]^+^, and [Cu­(L)]^+^, respectively, along with the assigned peak for the quinone sodium aggregate (i.e., [(3,5DTBQ) + Na^+^]) at *m*/*z* = 243.13, which confirms the formation of the final product. Under catalytic conditions, the minor peak at *m*/*z* = 708.98 is consistent with the dimeric species [Cu_2_(Cl_3_)­(L_2_)]^+^, indicating that such species with binuclear integrity persist during catalysis (see inset Figure S28, Supporting Information). It is evident that during the oxidation of 3,5-DTBC to 3,5-DTBQ, aerial oxygen can be reduced to H_2_O_2_ or H_2_O. To clarify this occurrence, the generation of H_2_O_2_ during the catalytic reaction was demonstrated by observing the emergence of the characteristic band for $I_{3}^{-}$ by spectrophotometry (λ_max_ = 353 nm; ε = 26,000 M^–1^ cm^–1^), upon reaction of I^–^ with the reaction mixture (Figure S30, Supporting Information). Based on a previous study of the mechanism in different published reports, a plausible mechanism of the catalytic activity of catechol oxidase has been given in Scheme 3.^87^

**Scheme 3:**
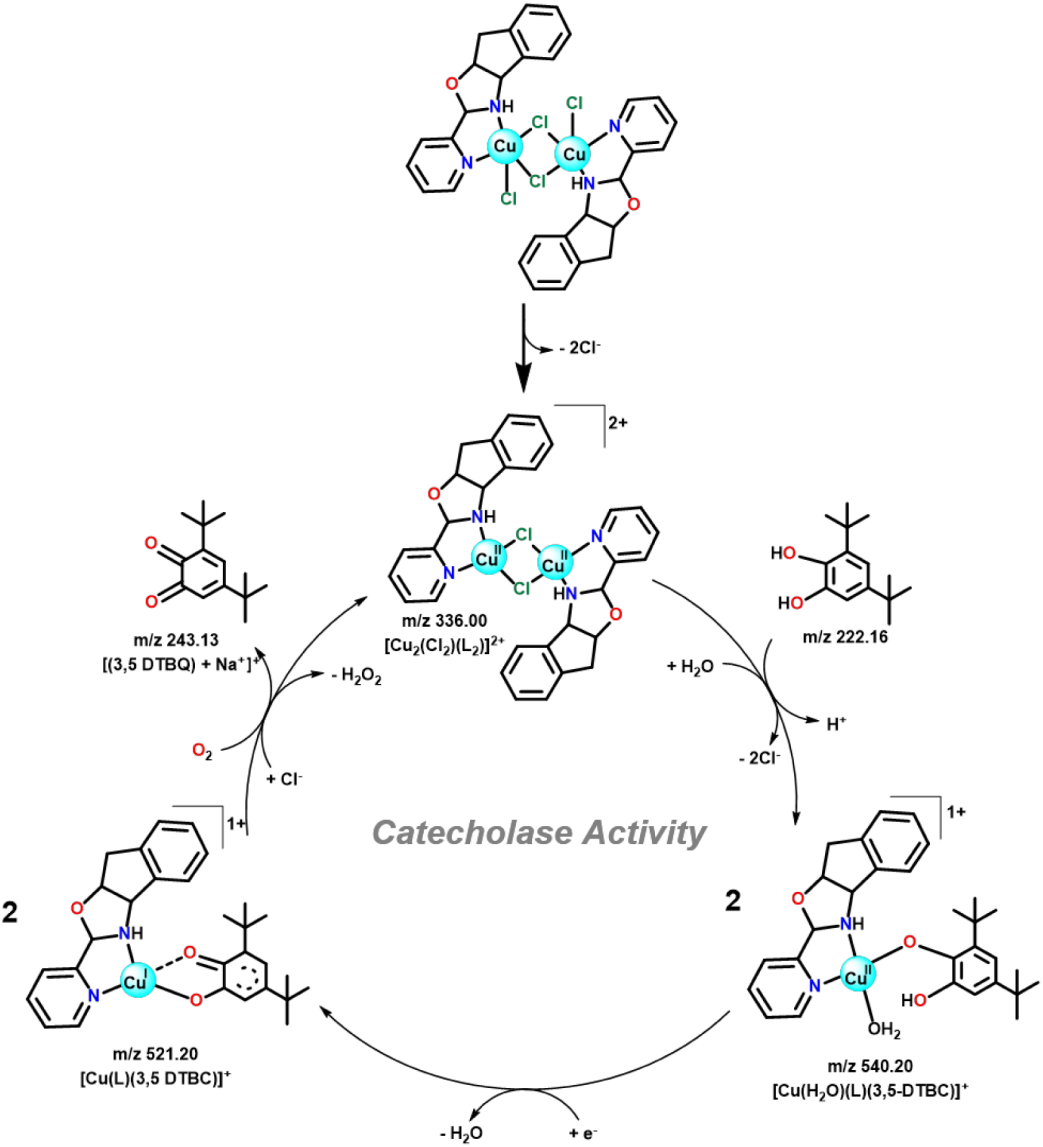
Probable Mechanistic Pathway for the Oxidation of 3,5-DTBC to 3,5-DTBQ by Complex **1**

##### 3.3.2.2 Oxidation of *o*-Aminophenol (OAPH)

The PHS-like activity of complex **1** was studied by treating different concentrations (5 × 10^–4^ M to 5 × 10^–3^ M) of the substrate, i.e., 2-aminophenol, with a 5 × 10^–5^ M concentration of the catalyst, i.e., complex **1,** in methanol at room temperature under aerobic conditions (see Scheme 4). The reaction was monitored spectrophotometrically between 300 and 650 nm at regular intervals. The characteristic peak at 437 nm confirms the PHS-like activity (see Figure 11).^88,89^ To maintain a pseudo first-order reaction, the catalyst-to-substrate ratio was always kept at least 1:10. Kinetic parameters were determined by applying the similar method that was applied in the analysis of the oxidation of 3,5-di-*tert*-butylcatechol (Figure 12a and 11b). The kinetic parameters show *k*_cat_ of 5.5 ± 0.6 × 10^3^ h^–1^ for the PHS activity catalyzed by complex **1**. Other parameters are summarized in Table S9. The PHS activity of complex **1** shows moderately high activity as compared to some reported values given in Table S9 in Supporting Information.^77,82,83,85,86,90–104^

**Scheme 4:**
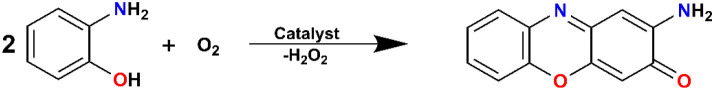
In the Presence of O_2_, Catalyst Oxidizes 2-Aminophenol into 2-Amino-phenoxazine-3-one

**Figure 11:**
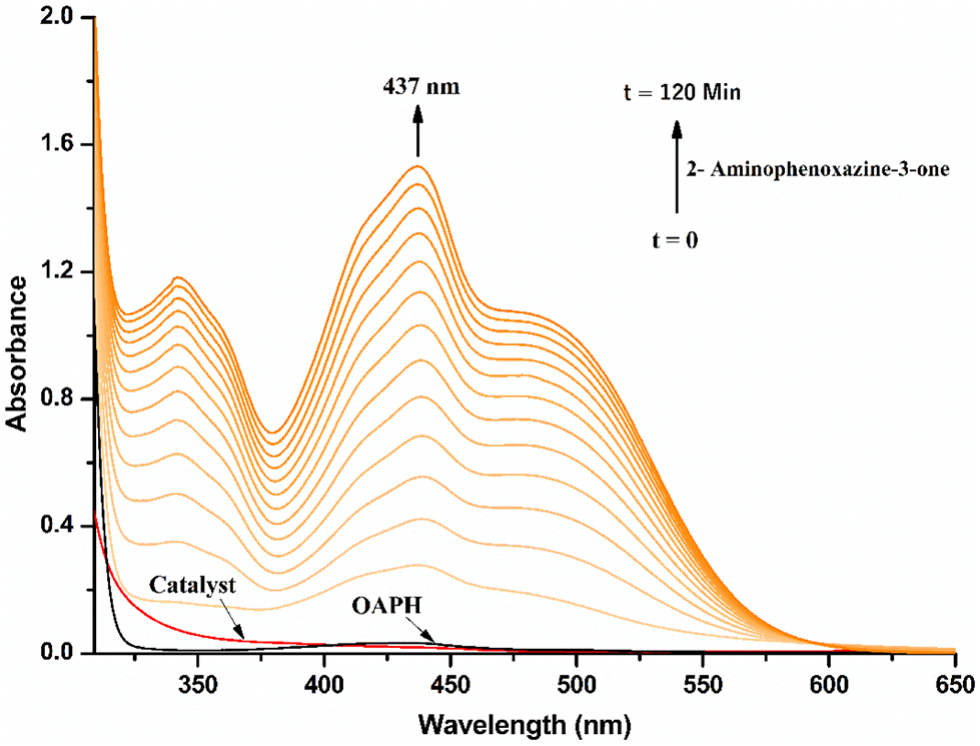
Oxidation of 2-aminophenol by complex **1** observed in MeOH by UV/vis spectroscopy over time.

**Figure 12:**
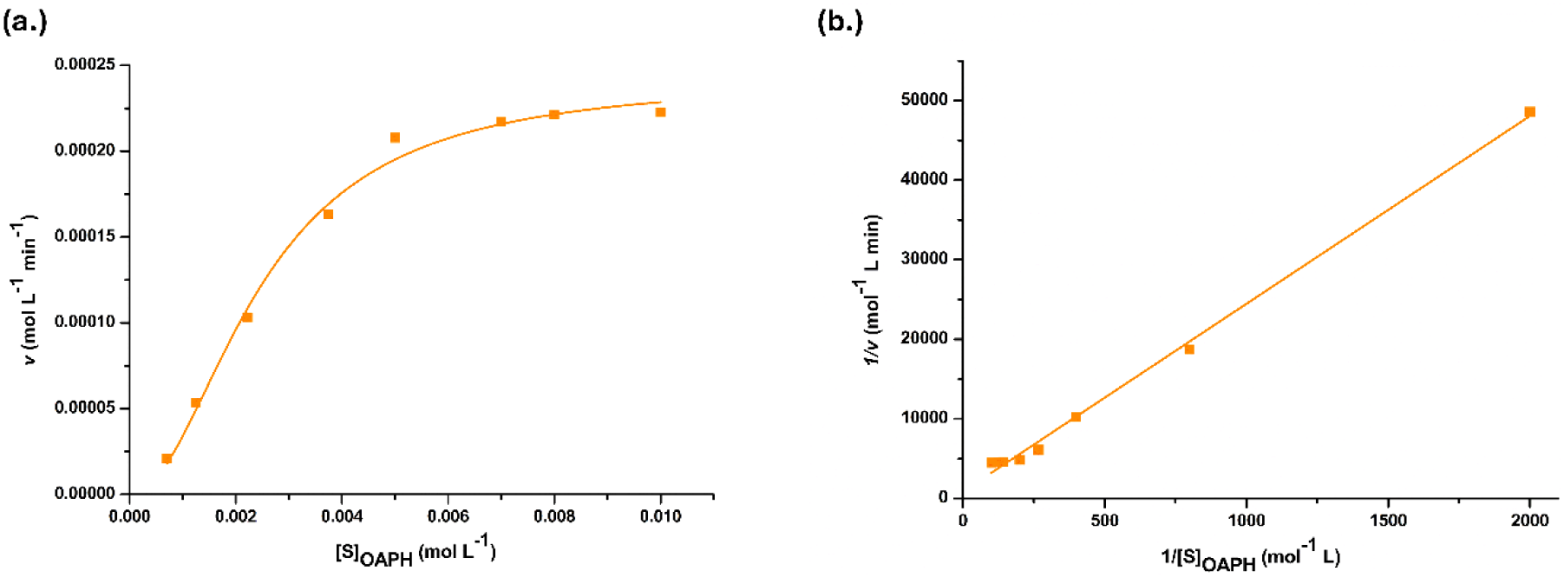
(a) Dependence of the rate of reaction on substrate concentration for complex **1** in methanol for oxidation of 2-aminophenol. (b) Lineweaver–Burk plot for complex **1** in MeOH medium for oxidation of 2-aminophenol.

The possible catalyst–substrate complex was elucidated by measuring the HRMS spectra of a 1:50 mixture of complex **1** with aminophenol in MeOH (Figure S29, Supporting Information). The base peak at *m*/*z* = 540.11 can be assigned to [(2-aminophenolate)_2_ Cu­(L)­Na^+^], and other prominent peaks at *m*/*z* = 336 and *m*/*z* = 499.07 correspond to intermediate-generated species, i.e., [Cu_2_(Cl_2_)­(L_2_)]^2+^ and [Cu­(Cl)­(2-aminophenol) (CH_3_OH) + Na^+^], respectively. The *m*/*z* = 301.04 and 110.06 are assigned due to the formation of the [Cu­(L)]^+^ fragment and [(2-aminophenol) + H^+^]. The *m*/*z* = 213.06 corresponds to the formation of the final product, i.e., [(2-aminophenoxazine-3-one) + H^+^]. A similar dimeric species, [Cu_2_Cl_3_(L)_2_]^+^, was also observed during the catalytic process, as evidenced by a minor peak at *m*/*z* = 708.98. To detect whether H_2_O or H_2_O_2_ is generated during the oxidative reaction of OAPH, a potassium iodide assay for $I_{3}^{-}$ measured spectrophotometrically at 353 nm confirms that H_2_O_2_ is generated (Figure S31, Supporting Information). Based on previously published mechanistic studies,^104^ a plausible mechanism for phenoxazinone synthase activity is given below in Scheme 5.

**Scheme 5:**
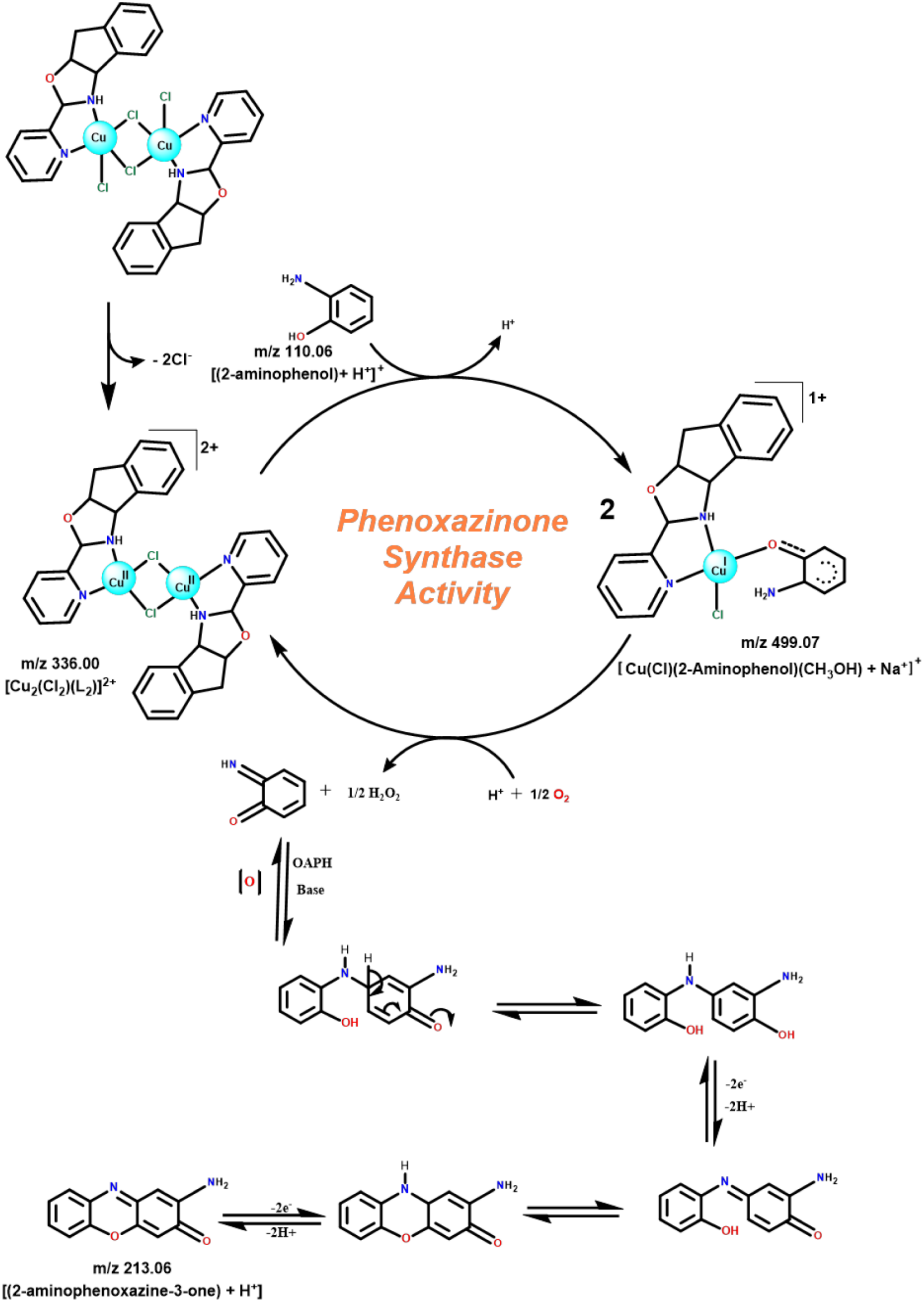
Probable Mechanistic Pathway for the Oxidation of *o*-Aminophenol to 2-Aminophenoxazine-3-one by Complex **1**

One important aspect of the current study is the bifunctionality of the Cu catalyst (Scheme 6). The bifunctionality of the Cu complex in this study arises from its reactivity in two independent reactions: a) in one case, it is used for hydroquinone and phenoxazinone derivative oxidation, where O_2_ acts as a terminal oxidant. Here, the efficacy of the Cu complex relies on its effective oxygen reduction reaction (ORR). b) On the other hand, the same catalyst has been used for the oxygen evolution reaction (OER). Thus, the catalyst can mediate both OER and ORR, making it a potential bifunctional catalyst.

**Scheme 6:**
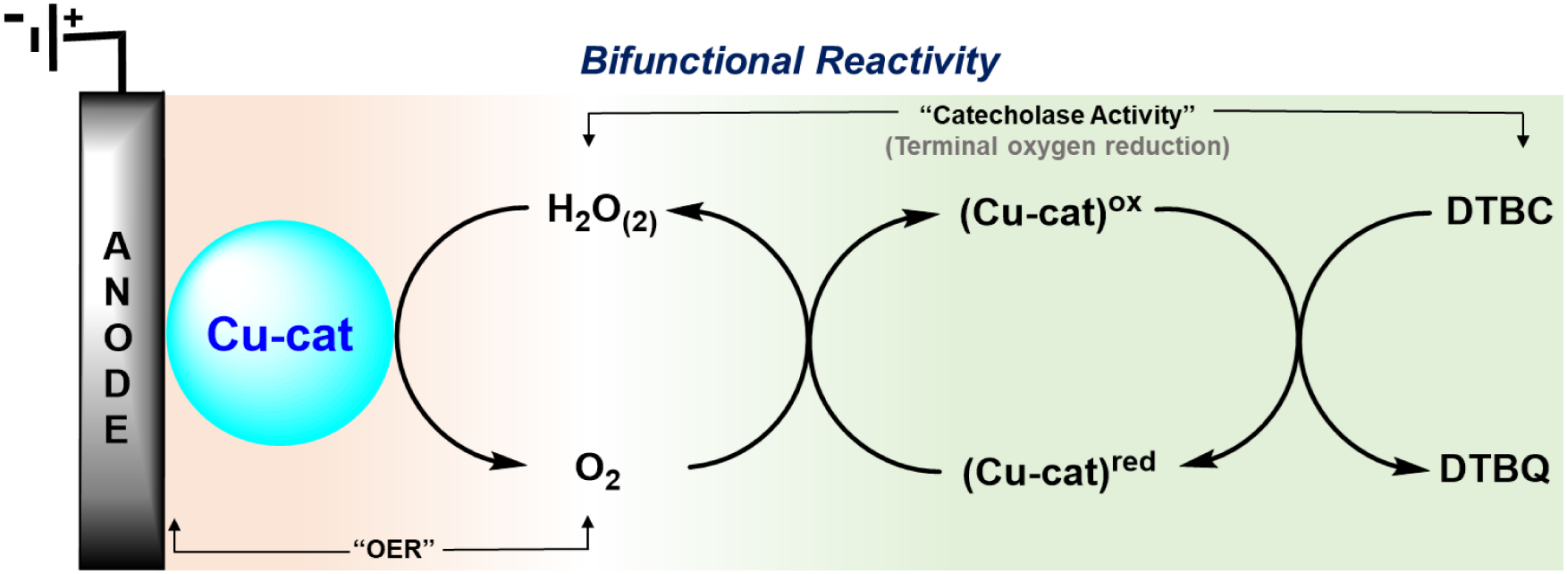
Bifunctionality of Chloro-Bridged Binuclear Cu Catalyst [Cu^II^_2_(μ_2_-Cl)_2_(Cl)_2_(L_1R2S_)_2_] for OER and Substrate Oxidation Using O_2_ as the Terminal Oxidant

## 4 Conclusion

This study successfully synthesized and characterized an oxazolidine-based copper complex through single-crystal X-ray diffraction (SCXRD). SCXRD analysis provides the structures of the complexes, revealing a distorted penta-coordinated square-pyramidal geometry. Through combining electrochemical analysis, kinetic study, and mechanistic investigation, our aim was to establish a structure–function relationship. According to our findings, the Faradaic efficiencies (FEs) for OER were calculated as 86 ± 6% for O_2_ and 76 ± 3% for H_2_. In addition, the high catalytic activity of the catalyst toward catecholase and phenoxazinone synthase activities can be a direct result of the two labile copper sites in the complex. These enzyme-mimicking model complexes help us to understand the mechanism of natural enzymes and rationally design catalysts with more efficient properties. In the future, the potential investigation of chirality-driven effects on asymmetric catalysis constitutes a promising domain. Furthermore, the chirality of the oxazolidine ring may open new areas in bioinspired applications, including enantioselective antimicrobial activity. Although this requires more in-depth study, our findings can provide valuable insight for the rational design of earth-abundant metal catalysts, which can tackle challenges in the emerging field of sustainable energy and oxidative chemistry.

## Supplementary Material







## Data Availability

The data are available throughout the manuscript and Supporting Files.

## Notes

Crystallographic data of Complex **1**(CIF)
Crystallographic data of Complex **2**(CIF)
Characterization results of Ligands (L_1R2S/1S2R_) and Complex 1/2 (^1^H NMR, ^13^C NMR, FTIR, UV–vis, HRMS and PXRD spectrum), structure refinement parameters, selected bond angles and bond lengths, continuous shape measurement (CShM) results, electrochemical measurements, HRMS of catalyst with substrate and comparative table for catalytic activity. The formula for the quantification of the electrochemical and kinetic processes (PDF)
