# Supplementary material for: Oxazolidine-Based Chloro-Bridged Dimeric Copper(II) Complex: A Bifunctional Catalyst for Enzyme-Mimetic Aerobic Oxidations and the Oxygen Evolution Reaction
Source: ACS Omega. 2026 Jul 22;11(30):44942–57. doi: 10.1021/acsomega.6c02828 (PMC13448911; doi:10.1021/acsomega.6c02828)
Supplement: Supplementary file 1 [file ao6c02828_si_003.pdf]

Oxazolidine-Based Chloro-Bridged Dimeric Copper(II) Complex: A Bifunctional Catalyst for Enzyme-Mimetic Aerobic Oxidations and the Oxygen Evolution Reaction

Pravesh Kumar [a], Vinod J [a], Tarisha Gupta [b], Ray J. Butcher [c], Biswajit Mondal [b]*, and Ritwik Modak [a]*

[a]Manipal Institute of Technology Bengaluru, Manipal Academy of Higher Education, Manipal 576104, India

[b]Department of Chemistry, IIT Gandhinagar, Palaj, Gujarat-382355, India

[c]Department of Chemistry, Howard University, WA, D.C. 20059, USA

*Email: ritwik.modak@manipal.edu

*Email: mondal.biswajit@iitgn.ac.in

SUPPORTING INFORMATION

1. ***Characterization, Structure and Crystallographic Data.***

NMR Spectroscopy: $\mathbf{L_{1R2S}}$ and $\mathbf{L_{1S2R}}$ were dissolved in $CDCl_3$ and recorded with TMS as internal standard on Bruker Biospin (400MHz) NMR system.

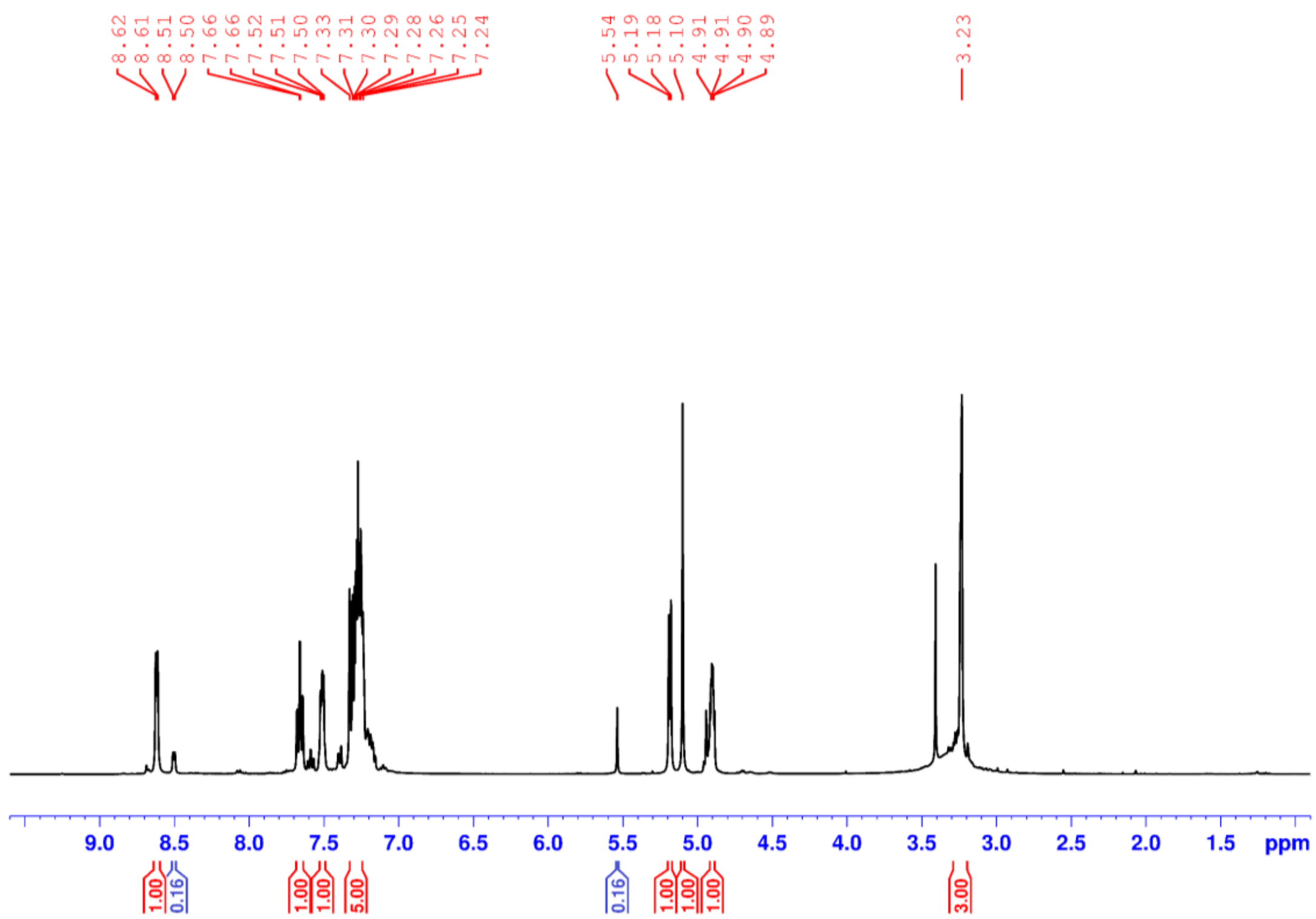

Figure S1. ^{1}H NMR (400 MHz, $CDCl_3$) spectrum of $\mathbf{L_{1R2S}}$. (Integration of the characteristic diastereotopic protons N–CH–O and HC=N_{Py} in other diastereomeric form are indicated with blue colour.)

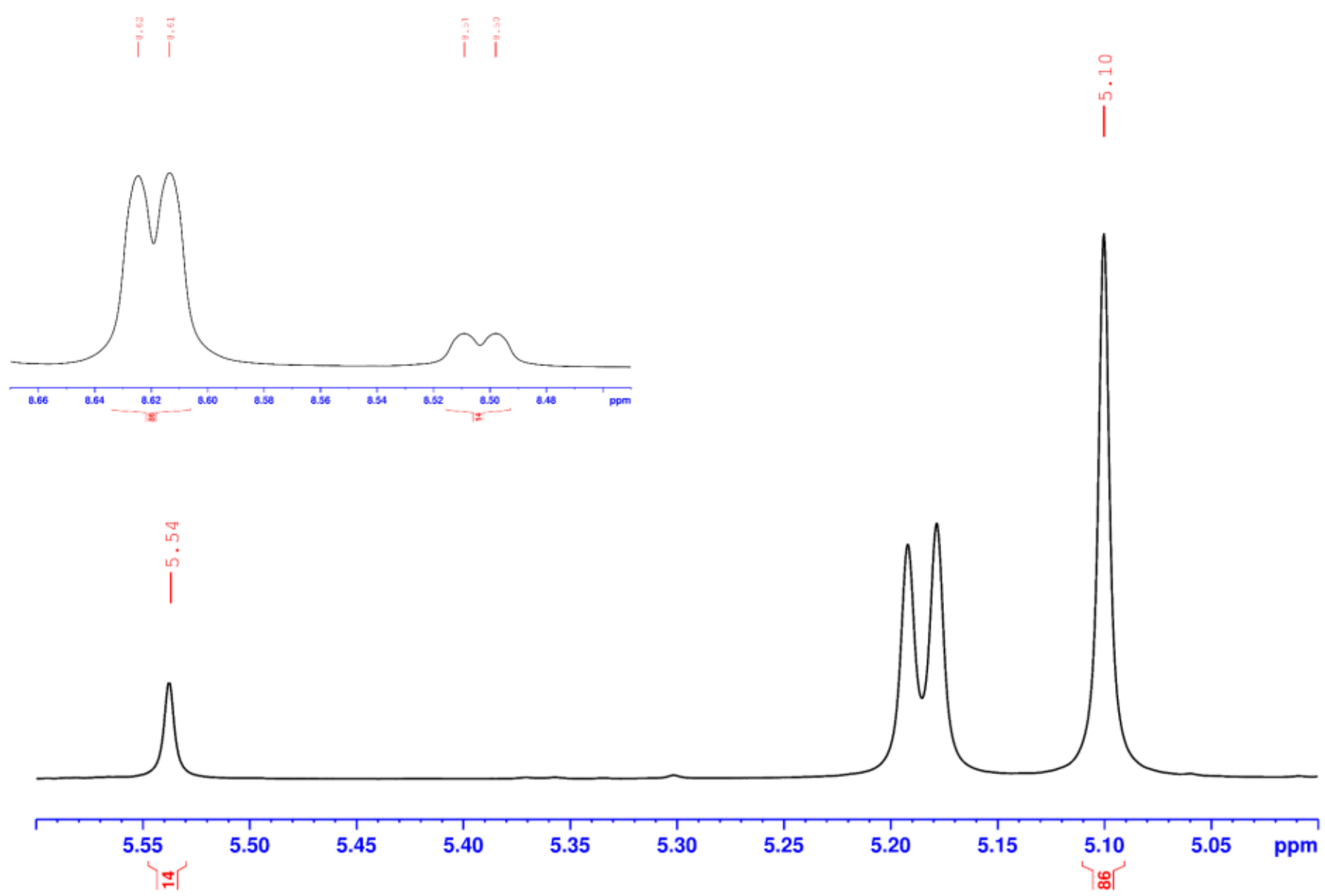

Figure S2. Part of ^{1}H NMR (400 MHz, $CDCl_3$) spectrum of $\mathbf{L_{1R2S}}$. Chemical shifts of the characteristic diastereotopic protons N–CH–O in the two diastereomeric forms are indicated, together with the amounts of two forms present (inset shows diastereotopic protons HC=N_{Py} in two forms).

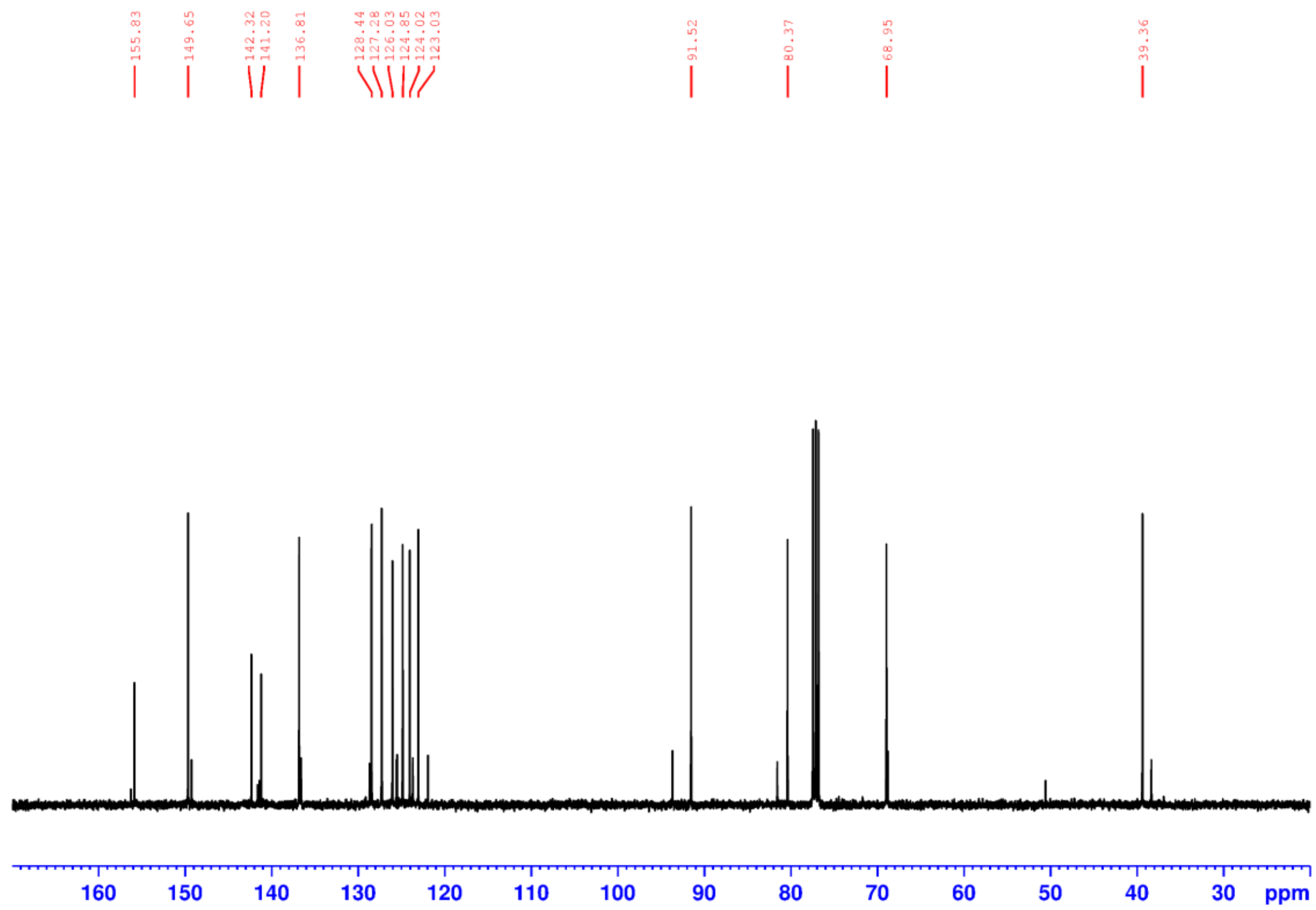

Figure S3. ^{13}C NMR (400 MHz, $CDCl_3$) spectrum of **L_{1R2S}**.

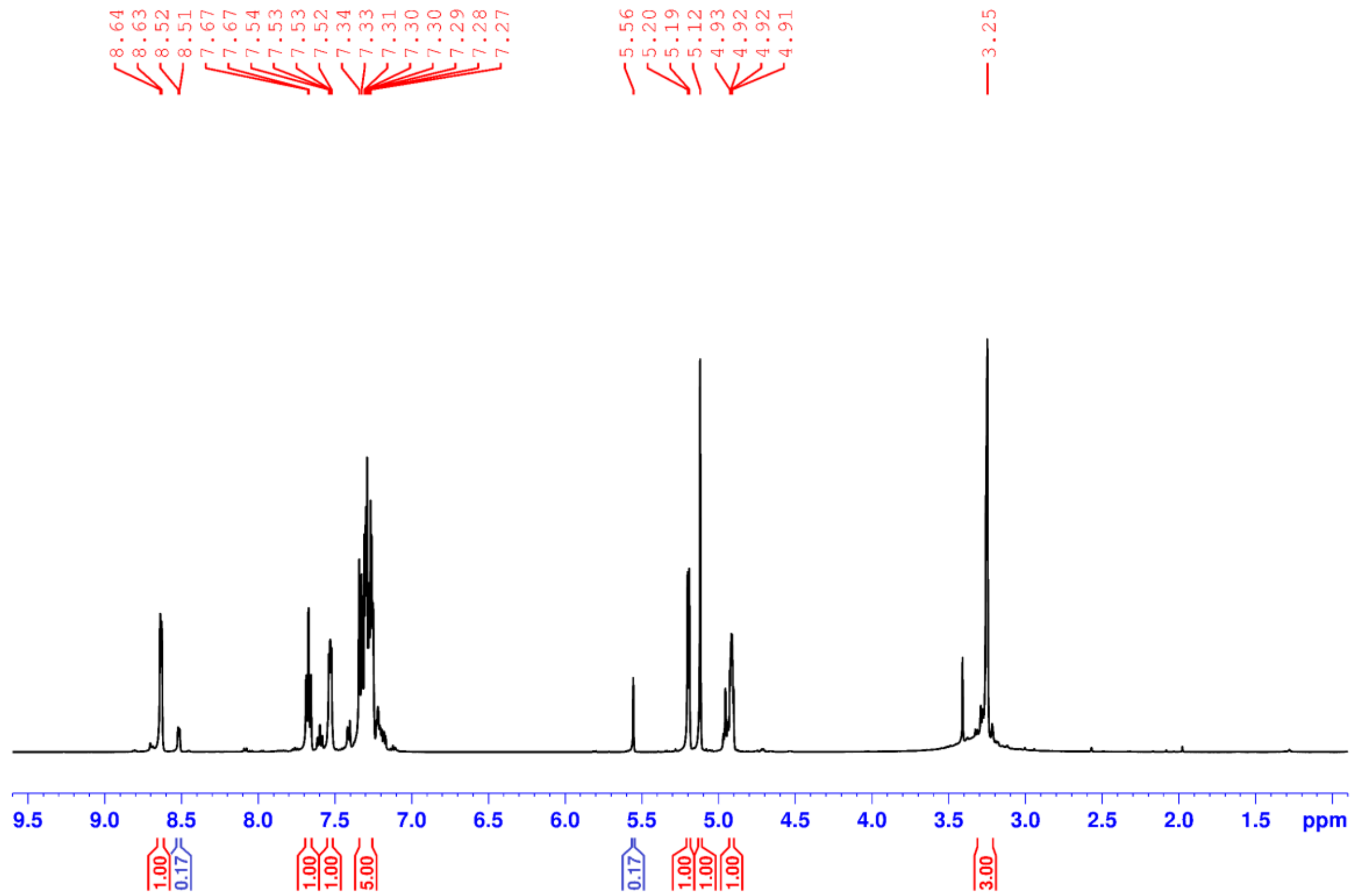

Figure S4. ^{1}H NMR (400 MHz, $CDCl_3$) spectrum of $\mathbf{L_{1S2R}}$. (Integration of the characteristic diastereotopic protons N–CH–O and HC=N_{Py} in other diastereomeric form are indicated with blue colour.)

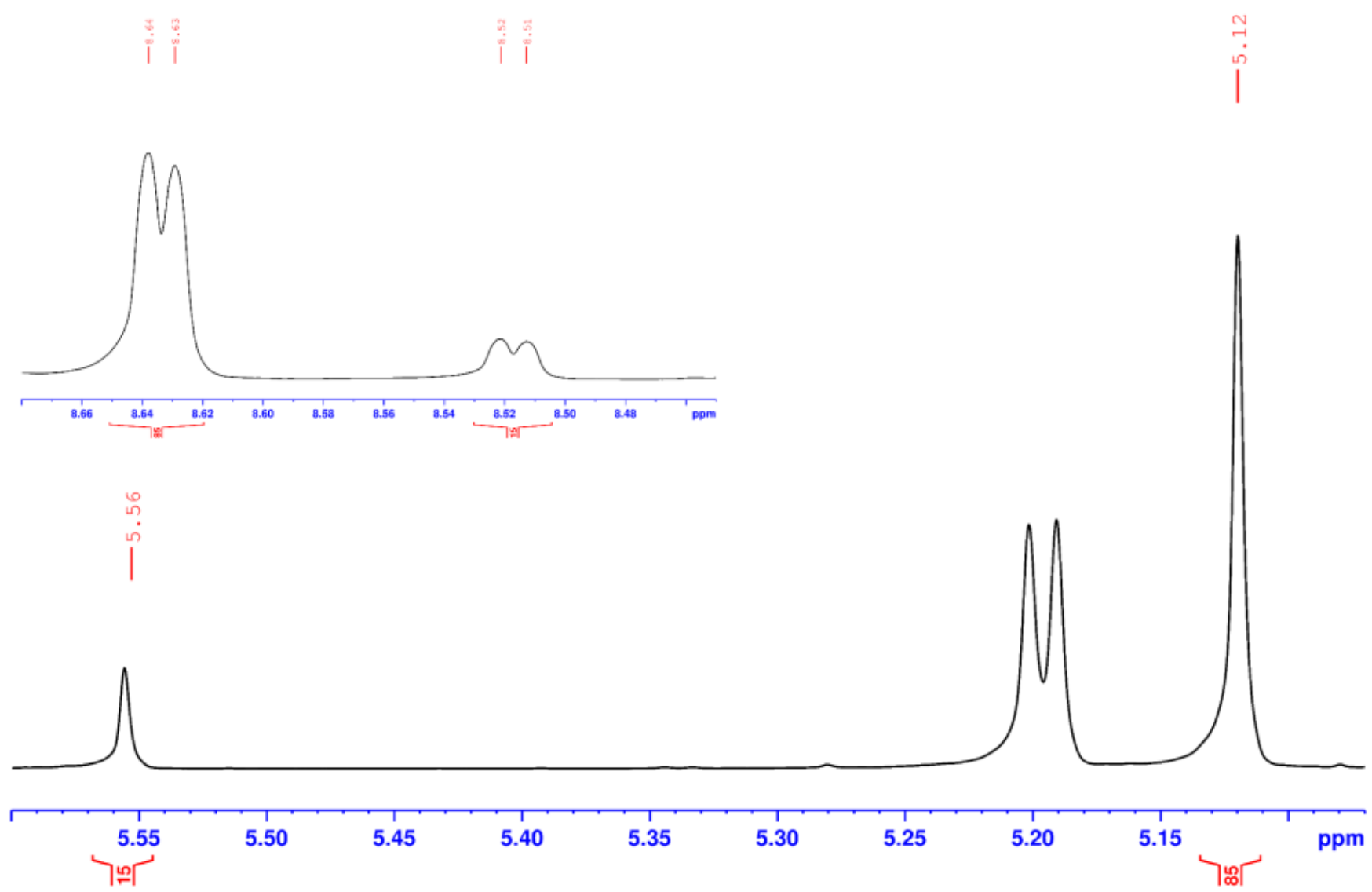

Figure S5. Part of ^{1}H NMR (400 MHz, $CDCl_3$) spectrum of $\mathbf{L_{1S2R}}$. Chemical shifts of the characteristic diastereotopic protons N–CH–O in the two diastereomeric are indicated, together with the amounts of two forms present (inset shows diastereotopic protons $HC{=}N_{Py}$ in two forms).

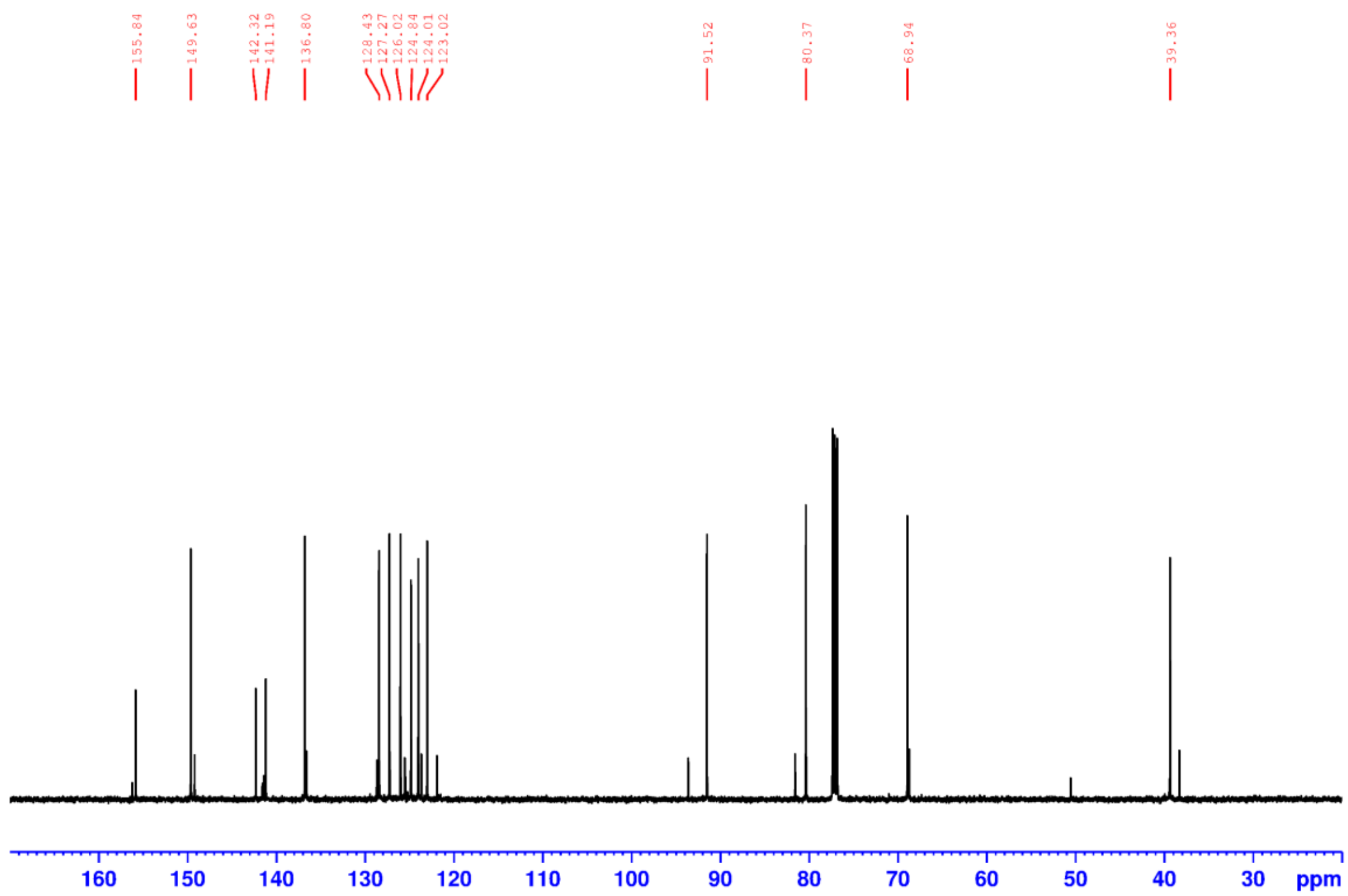

Figure S6. ^{13}C NMR (400 MHz, $CDCl_3$) spectrum of $\mathbf{L_{1S2R}}$.

FT−IR Spectroscopy: Fourier transform infrared (FT−IR) spectra were recorded with a Bruker ALPHA II Compact FT-IR Spectrometer in 4000-400 cm $^{-1}$ range.

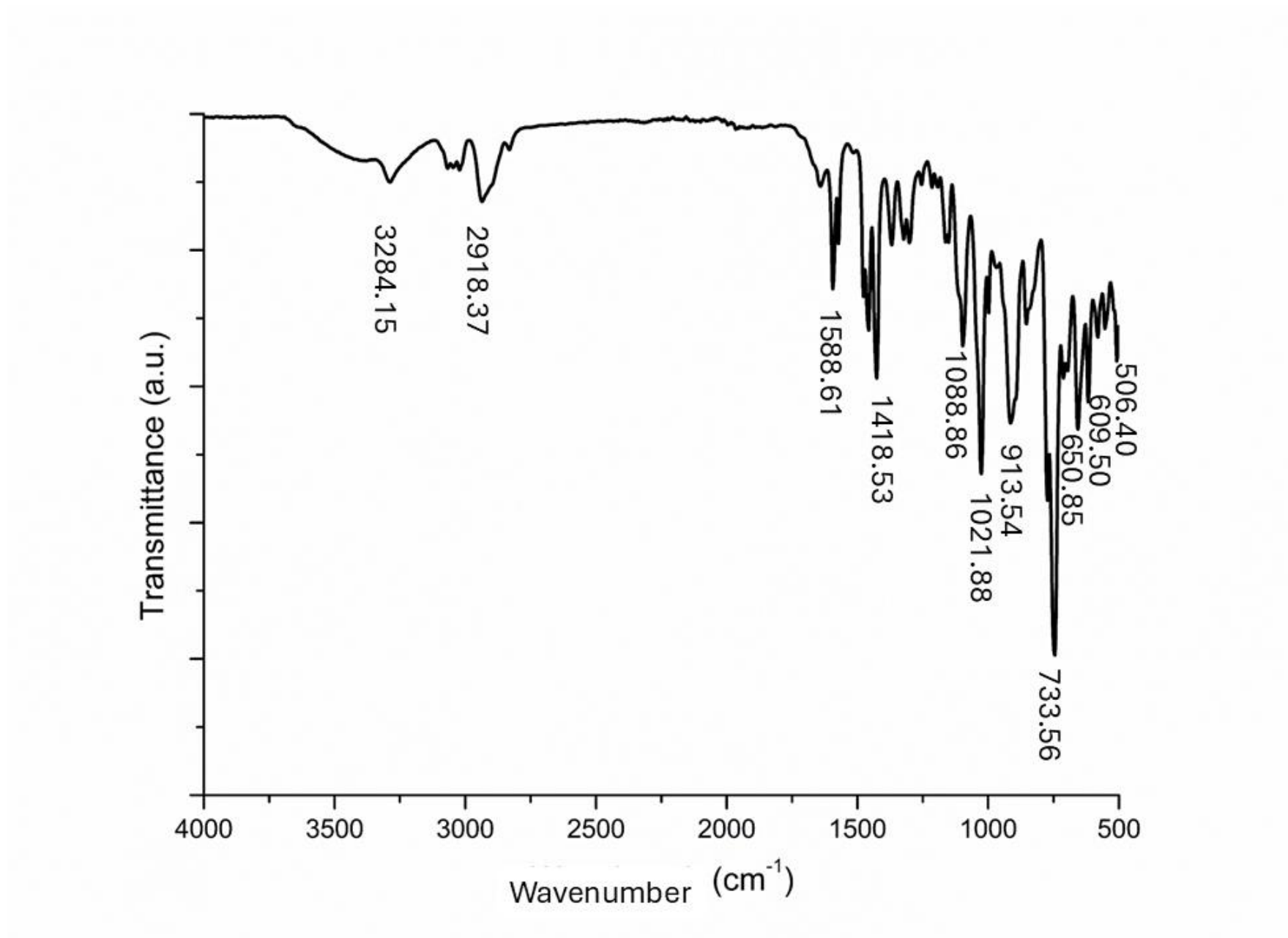

Figure S7. FT-IR spectrum of $\mathbf{L_{1R2S}}$.

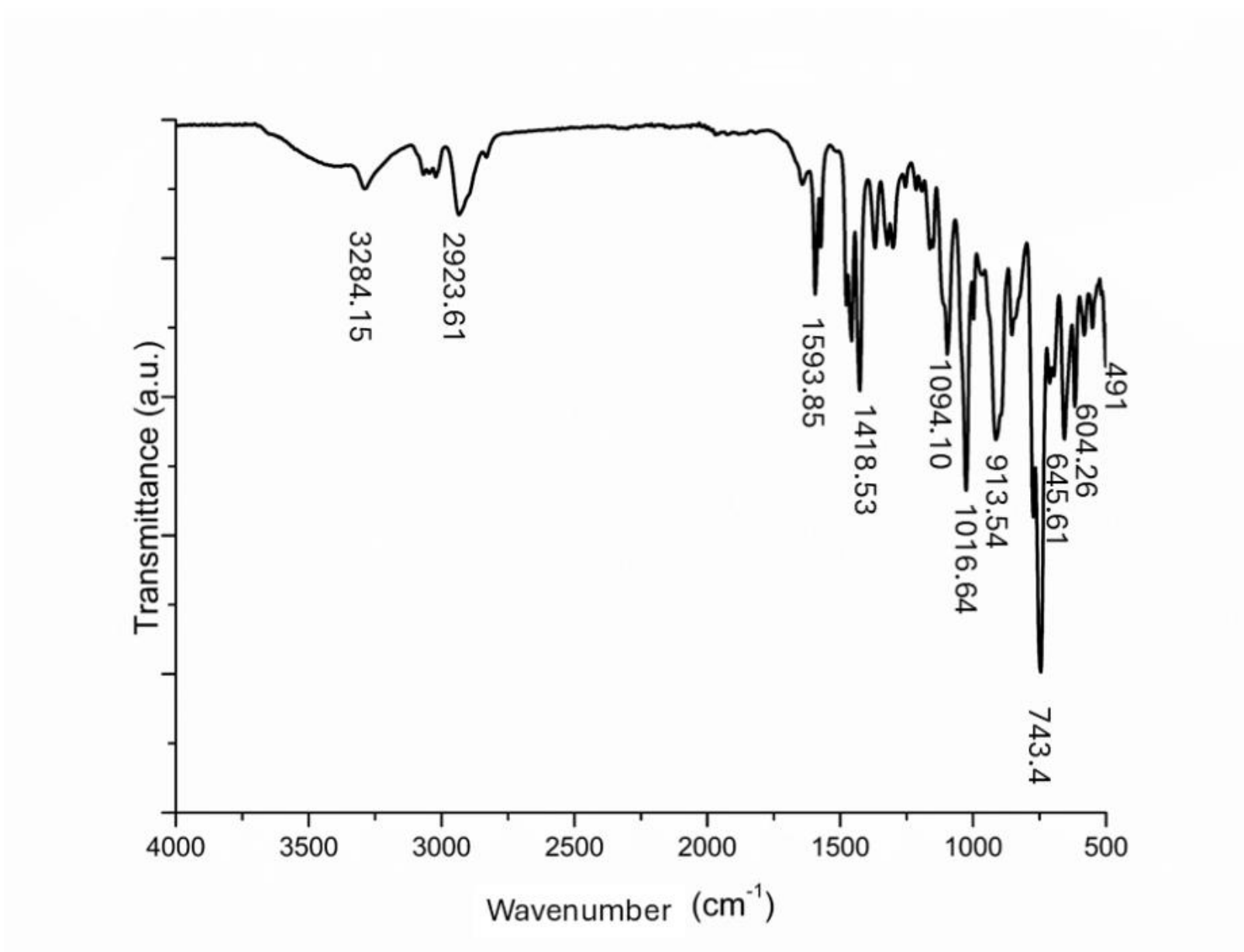

Figure S8. FT-IR spectrum of $\mathbf{L_{1S2R}}$.

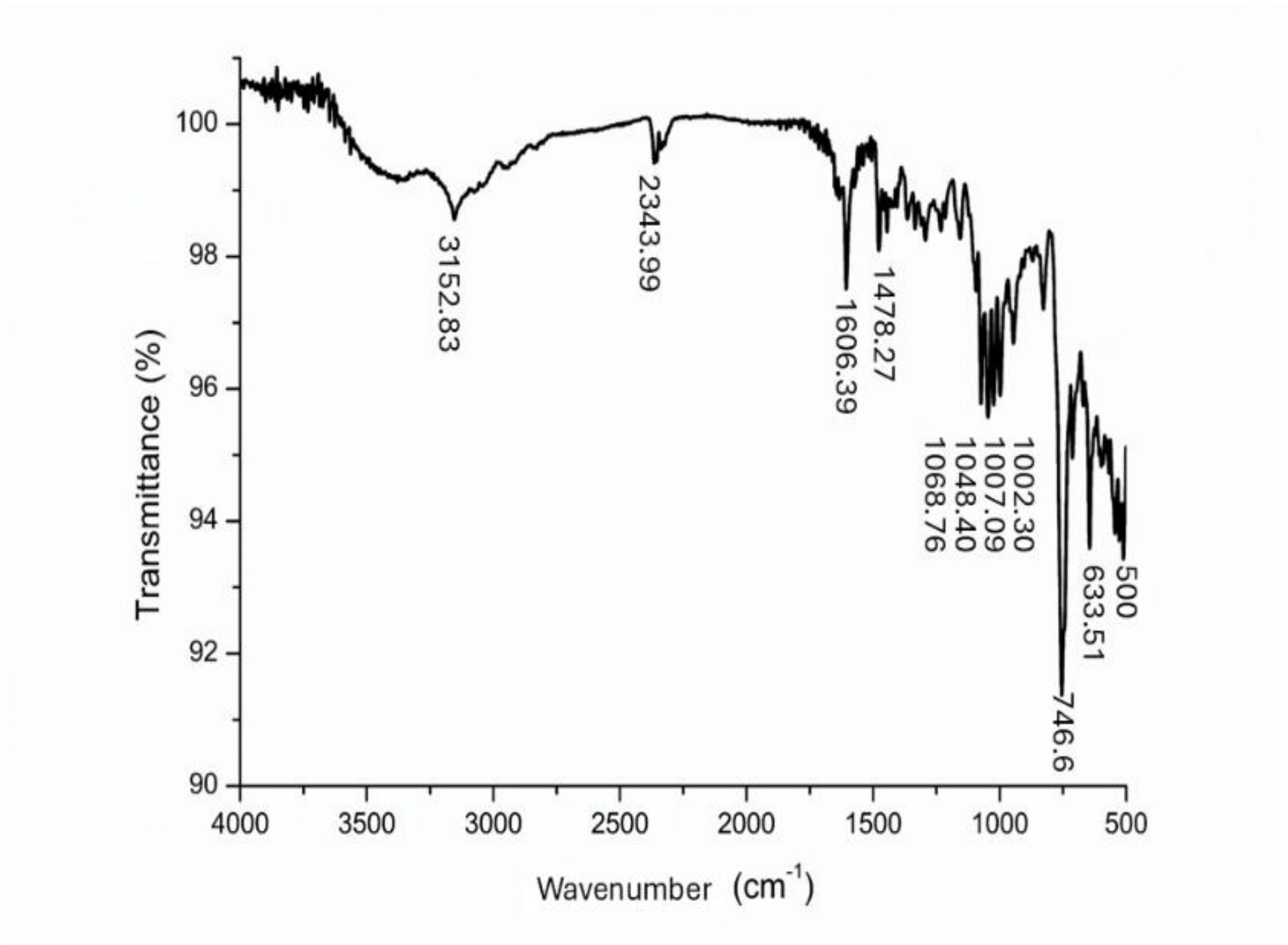

Figure S9. FT-IR spectrum of complex **1**.

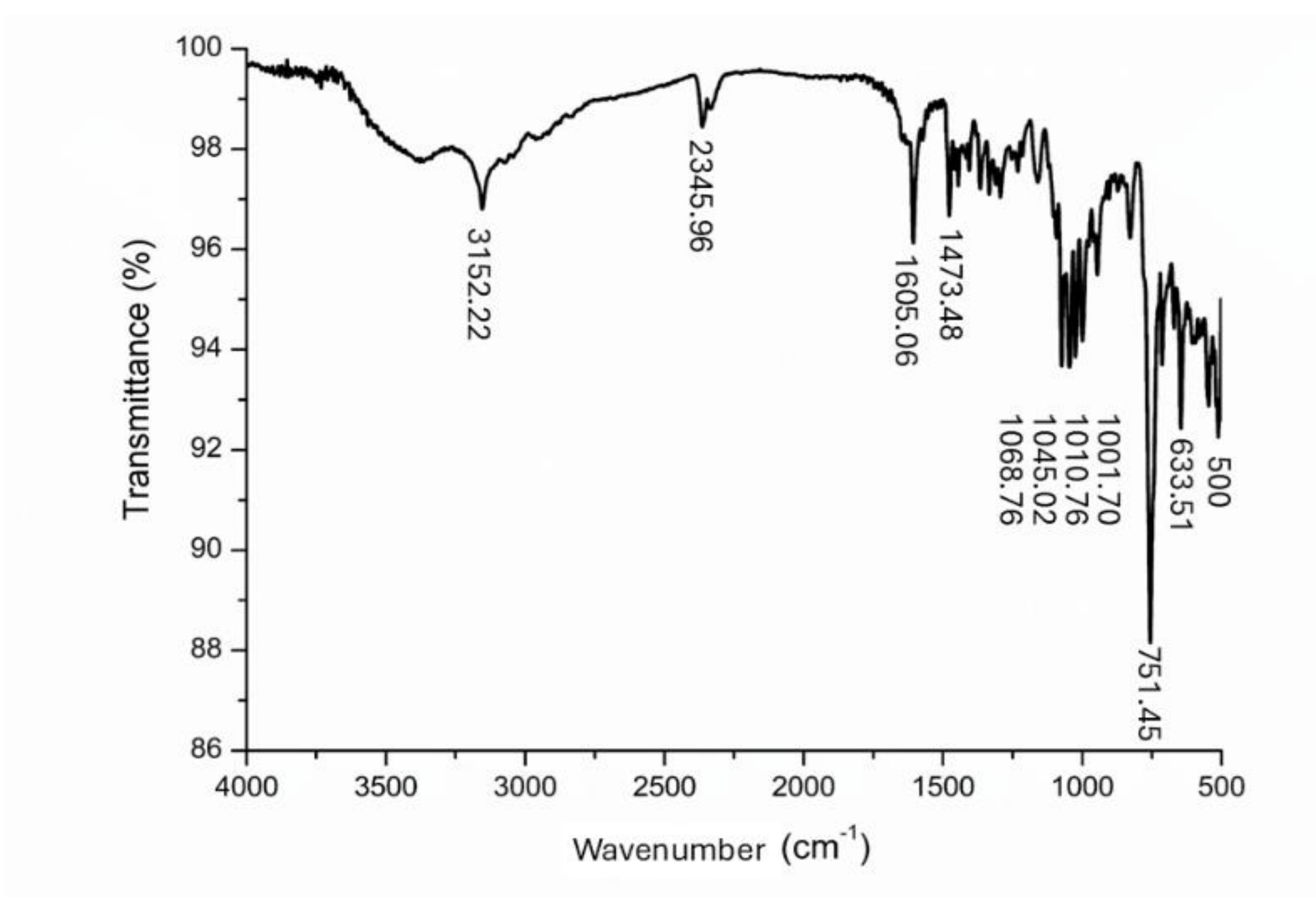

Figure S10. FT-IR spectrum of complex **2**.

UV-Vis Spectroscopy: Absorption spectra of methanolic solution (10^{-5} M) of **$L_{1R2S/1S2R}$** and complex **1/2** were recorded on the Shimadzu UV-3600 I Plus at room temperature with quartz cuvette of 1 cm path length cuvette.

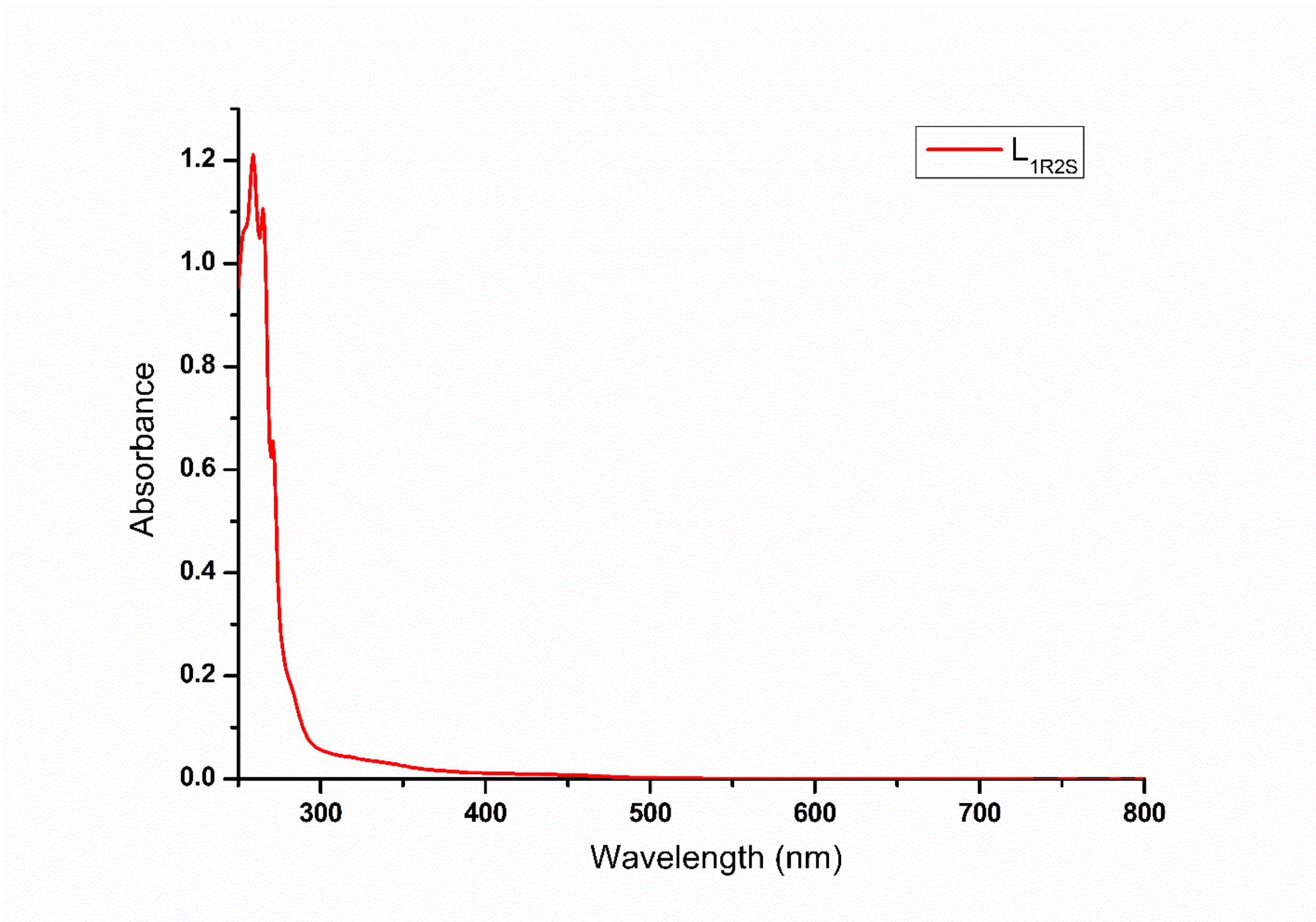

Figure S11. UV-Vis spectra of **L_{1R2S}** (10^{-5} M) in Methanol.

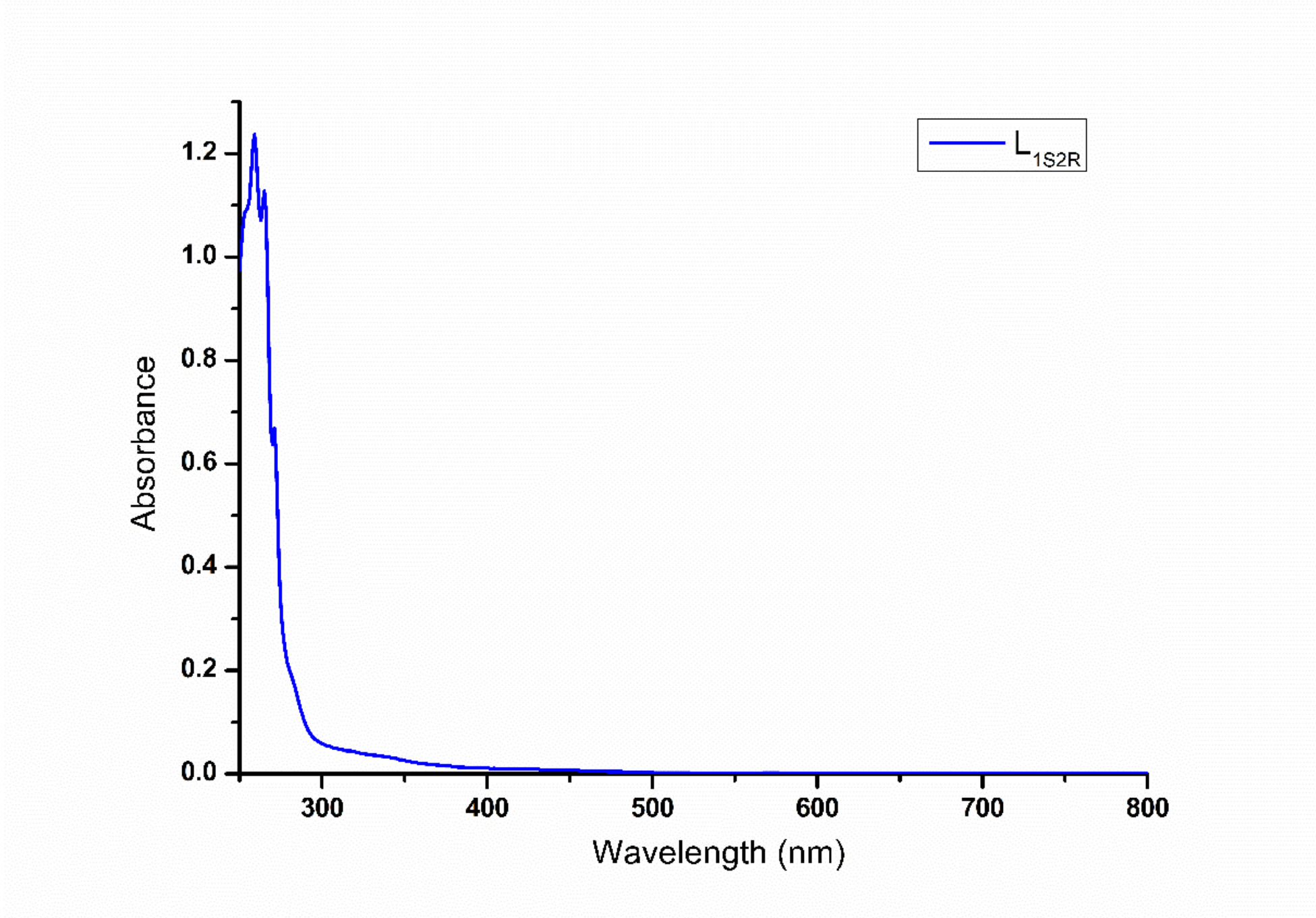

Figure S12. UV-Vis spectra of $\mathbf{L_{1S2R}}$ (10^{-5} M) in Methanol.

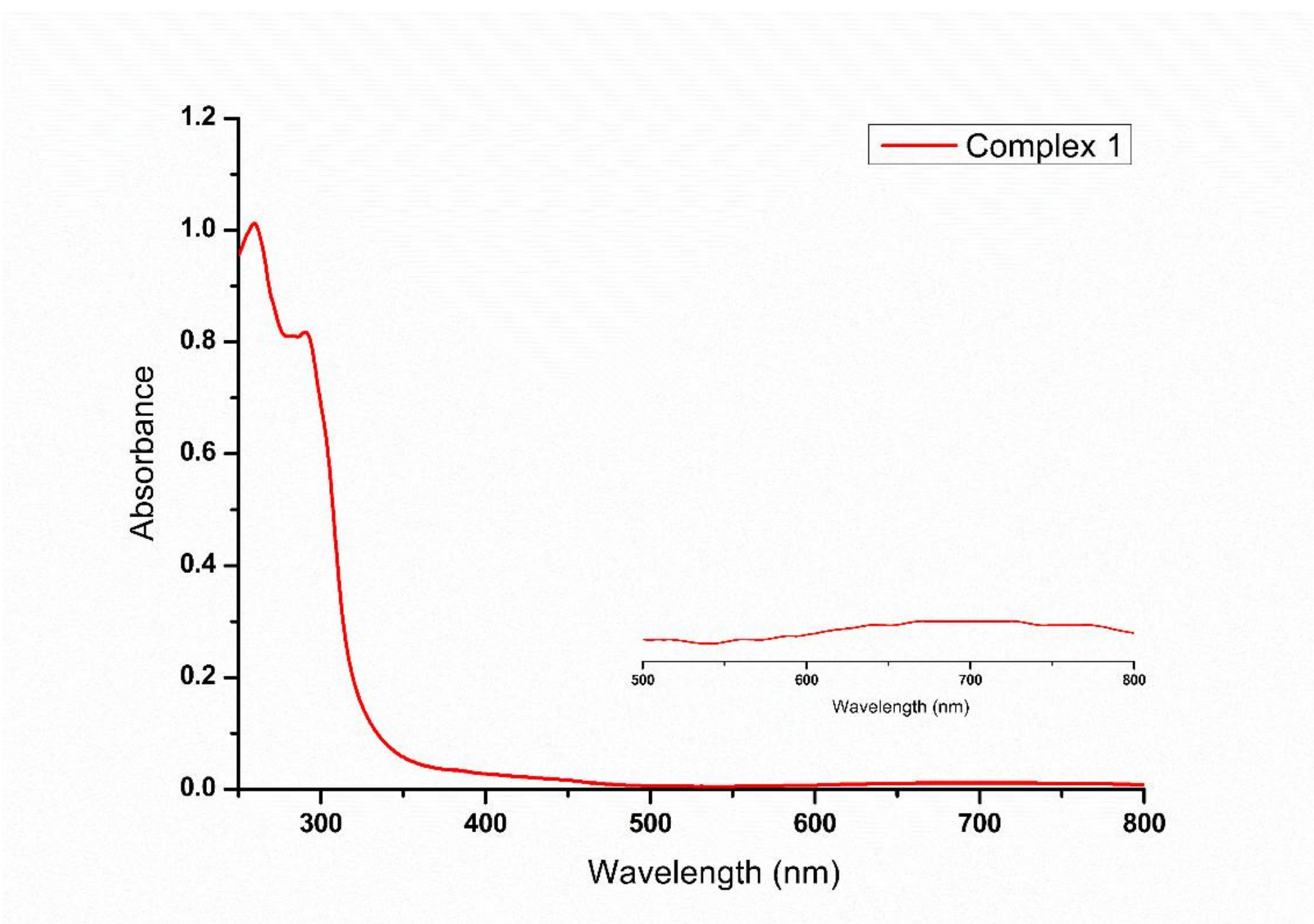

Figure S13. UV-Vis spectra of complex **1**(10^{-5} M) in Methanol (inset : magnified view of the 500-800 nm region).

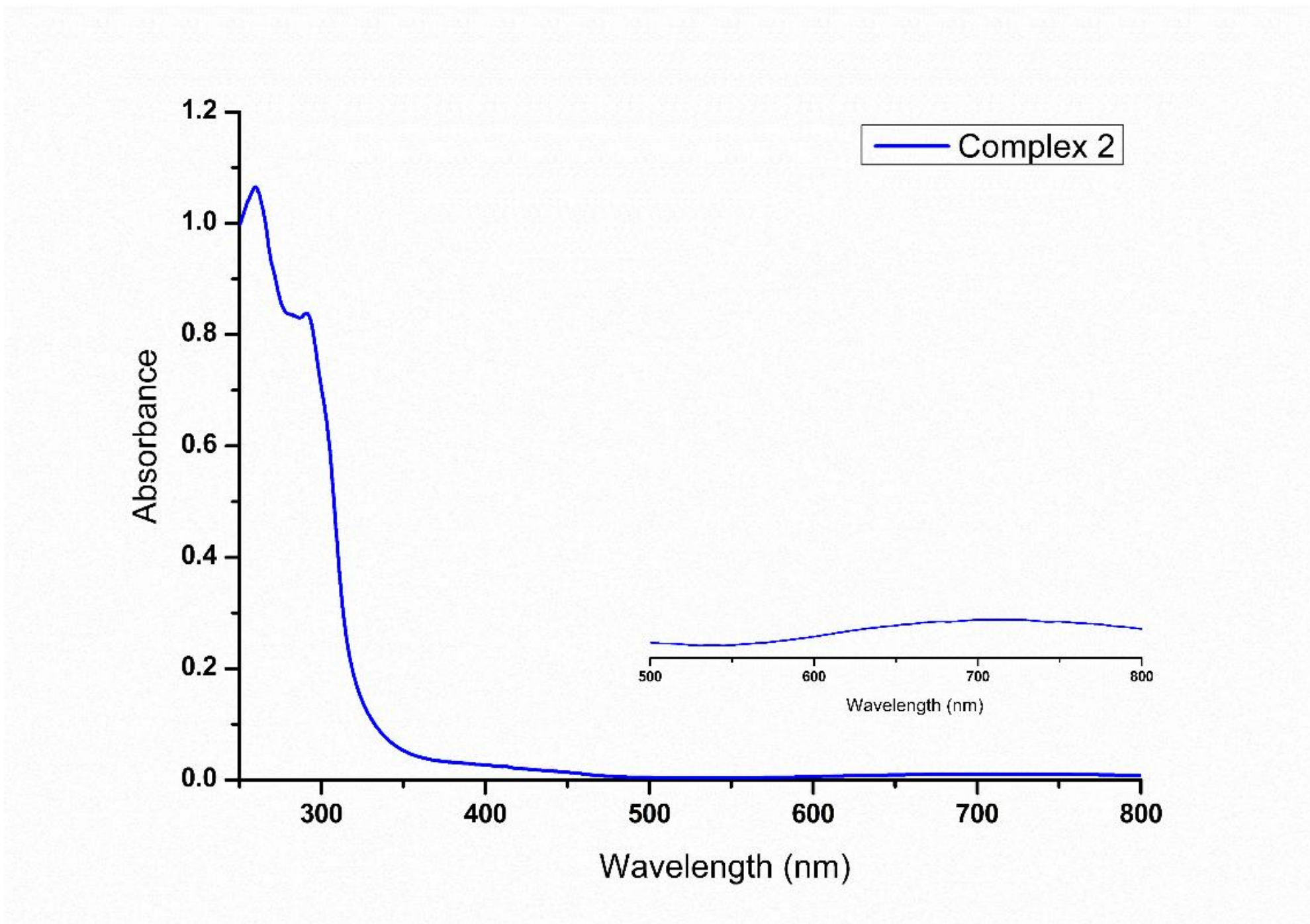

Figure S14. UV-Vis spectra of complex **2** (10^{-5}) M in Methanol (inset : magnified view of the 500-800 nm region).

ESI-HRMS: Electrospray Ionization High-Resolution Mass Spectra (ESI-HRMS) recorded in Methanol with Waters Synapt-G2S ESI-Q-TOF Mass instrument operating in positive ion mode.

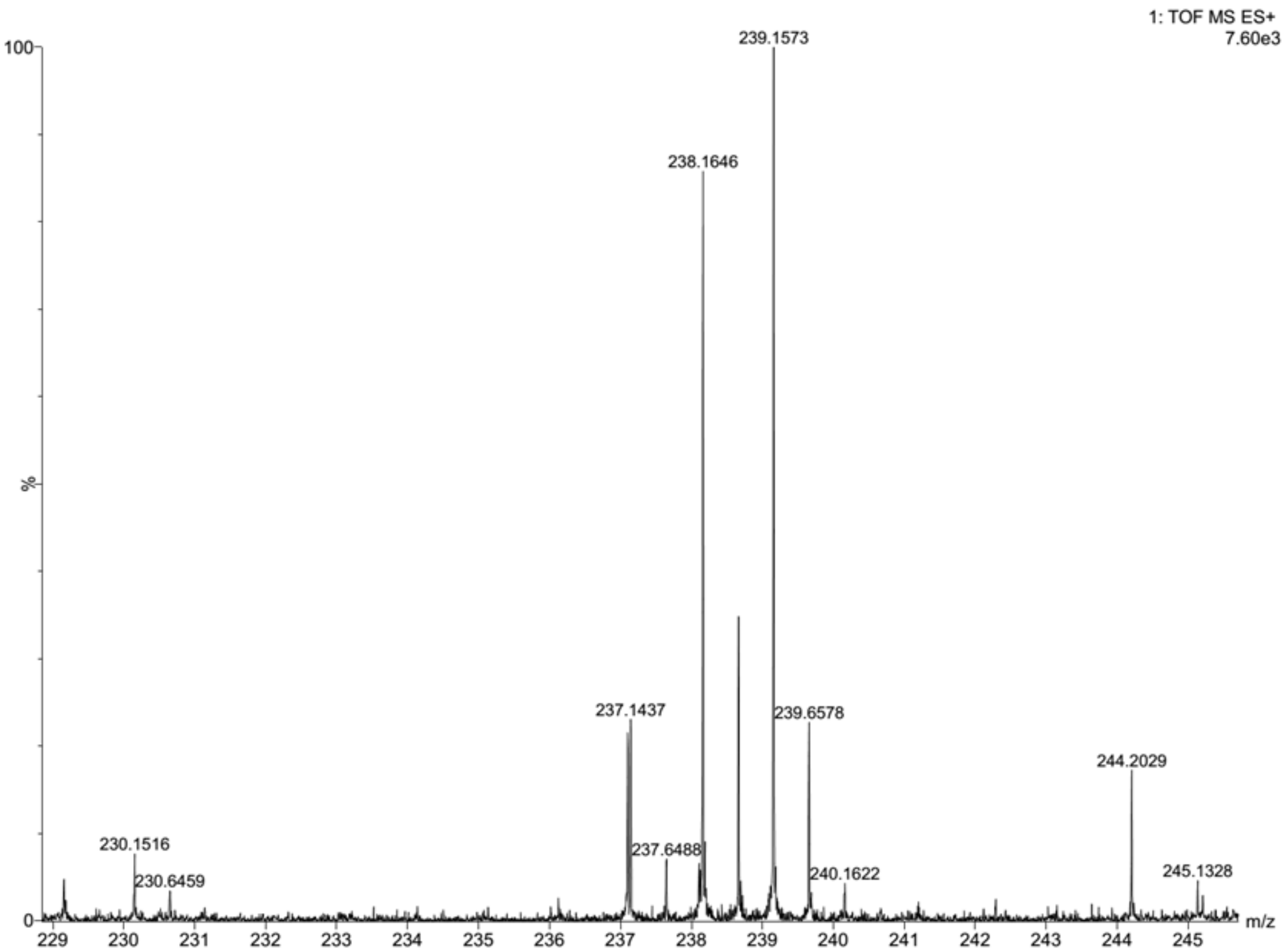

Figure S15. HRMS spectrum of $\mathbf{L_{1R2S}}$ recorded in Methanol.

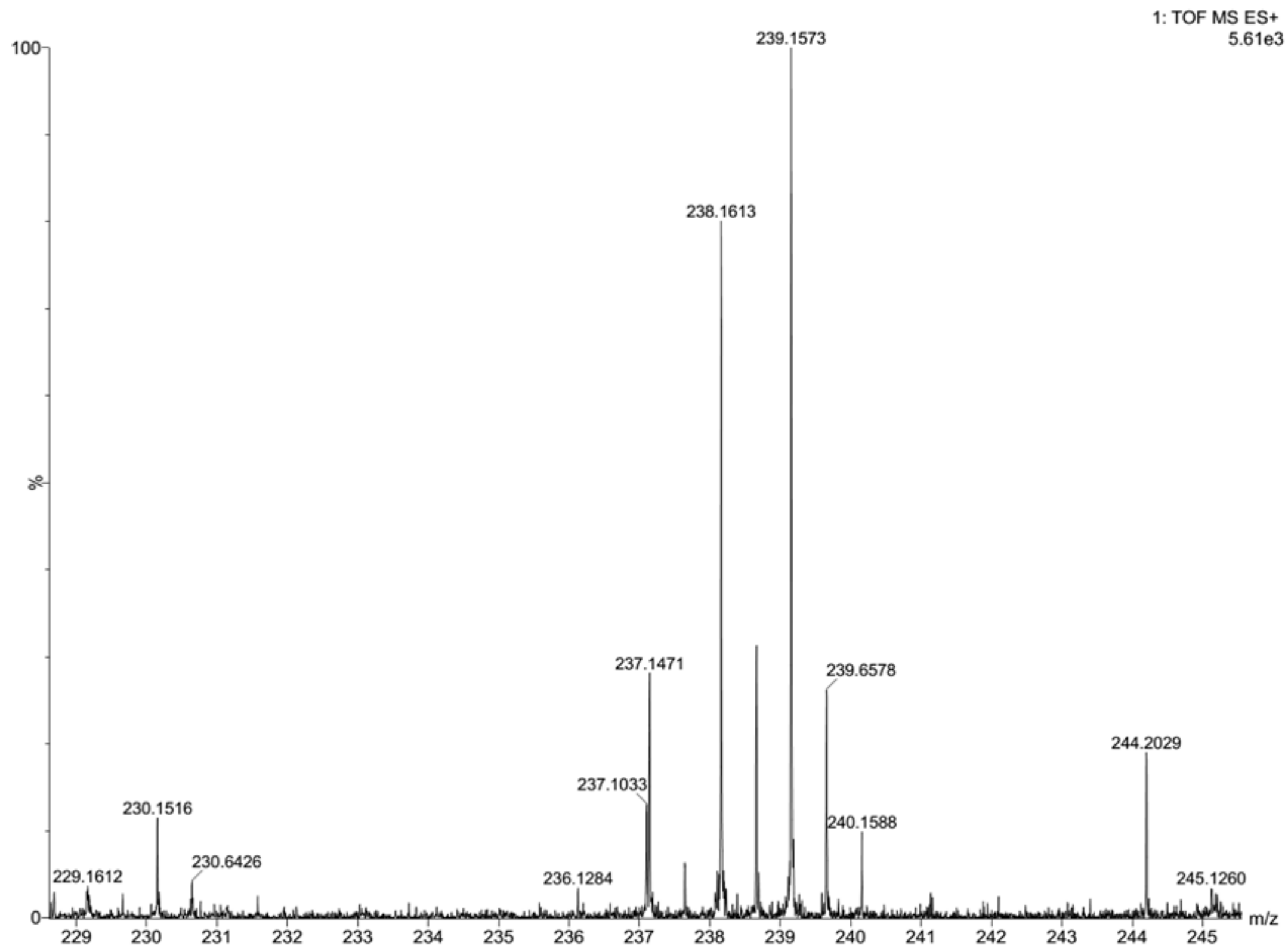

Figure S16. HRMS spectrum of $\mathbf{L_{1S2R}}$. recorded in Methanol.

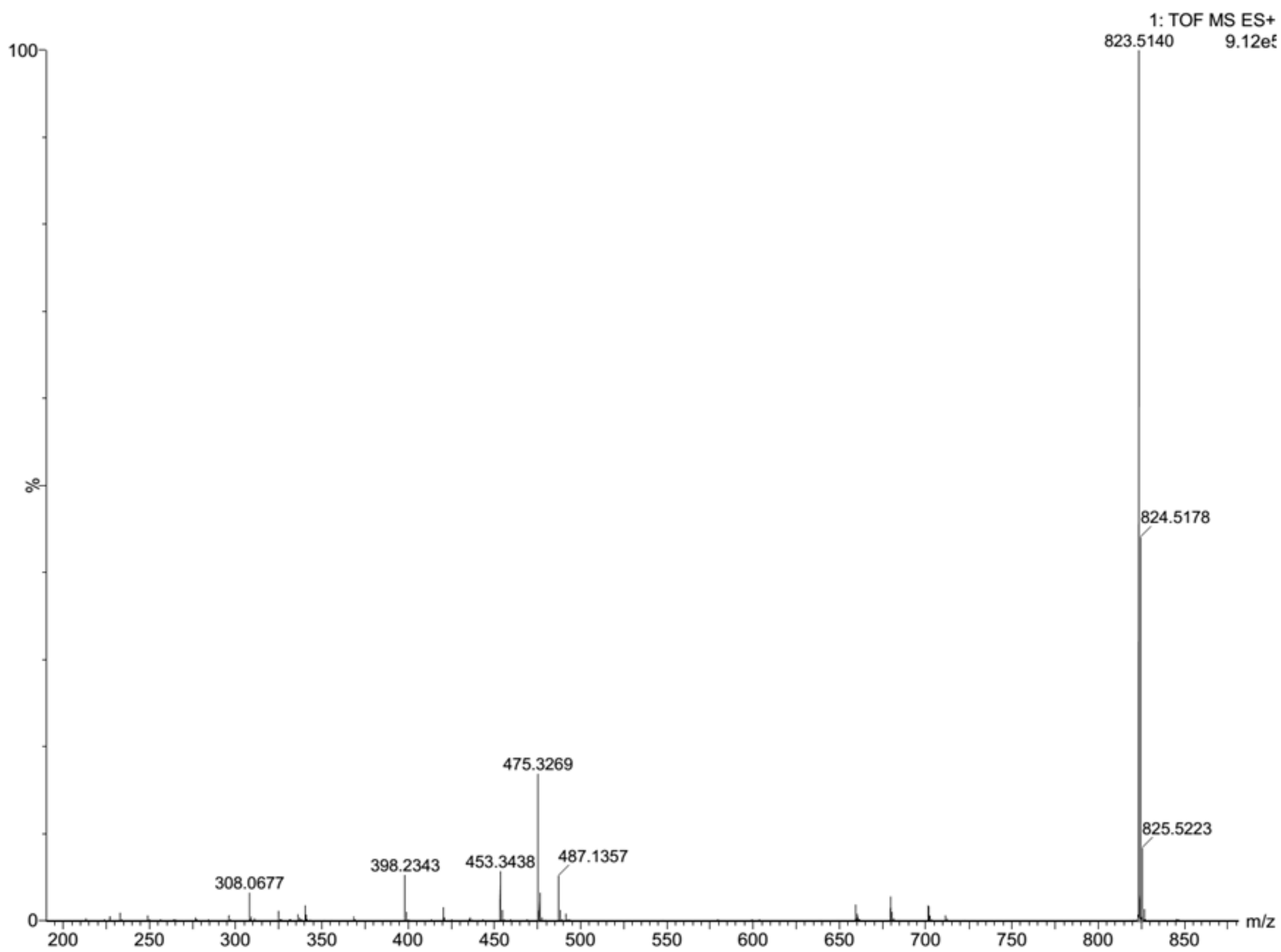

Figure S17. HRMS spectrum of complex **1** recorded in Methanol.

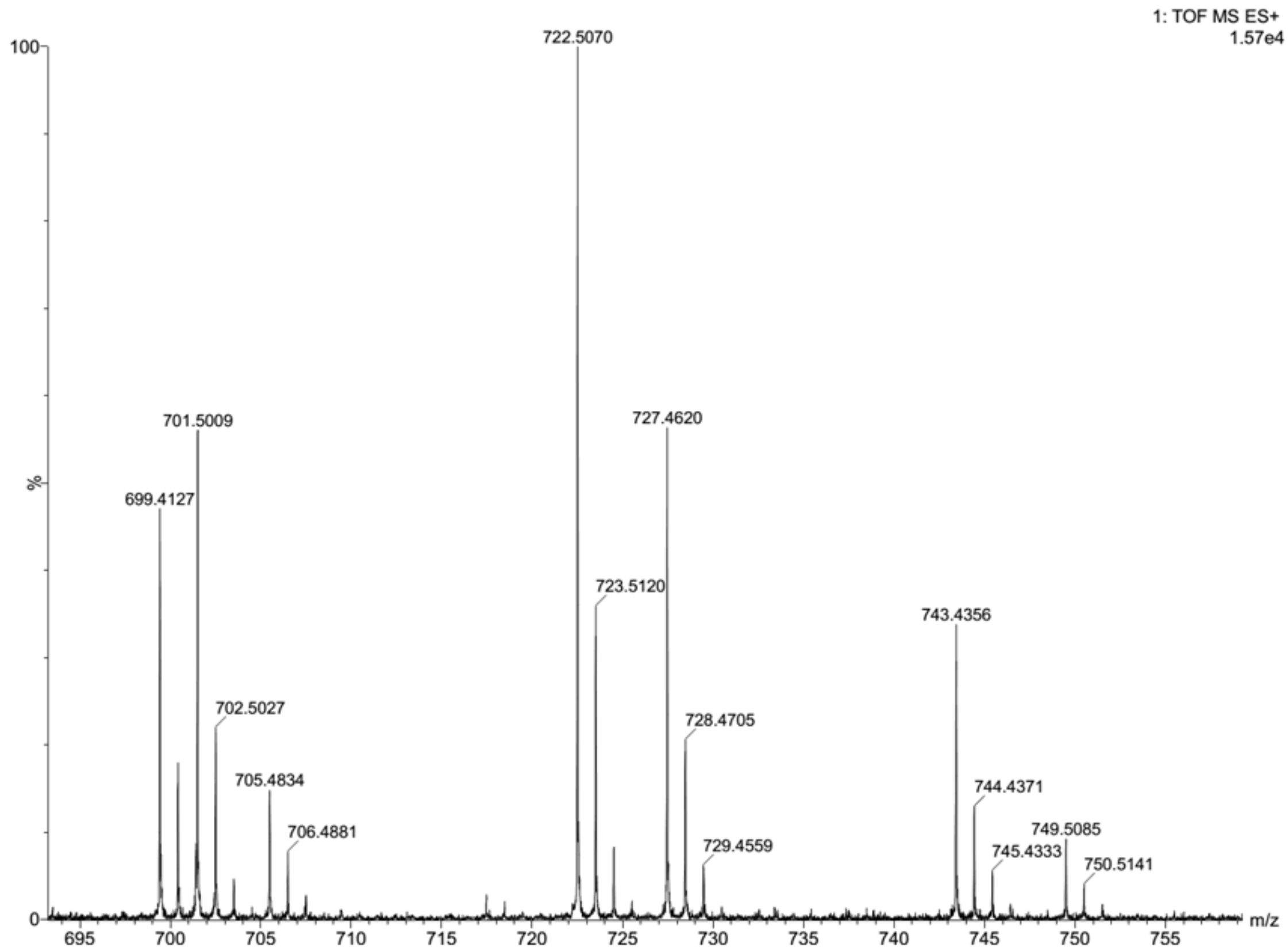

Figure S18. HRMS spectrum of complex **1** with close range recorded in Methanol.

Powder X-ray diffraction: (PXRD) patterns were recorded on Rigaku SmartLab X-ray Diffractometer instrument, with data recorded within 2θ range of 5 to 60°. The simulated patterns were calculated from the single crystal structural data (cif file) using the CCDC Mercury 3.1 software.

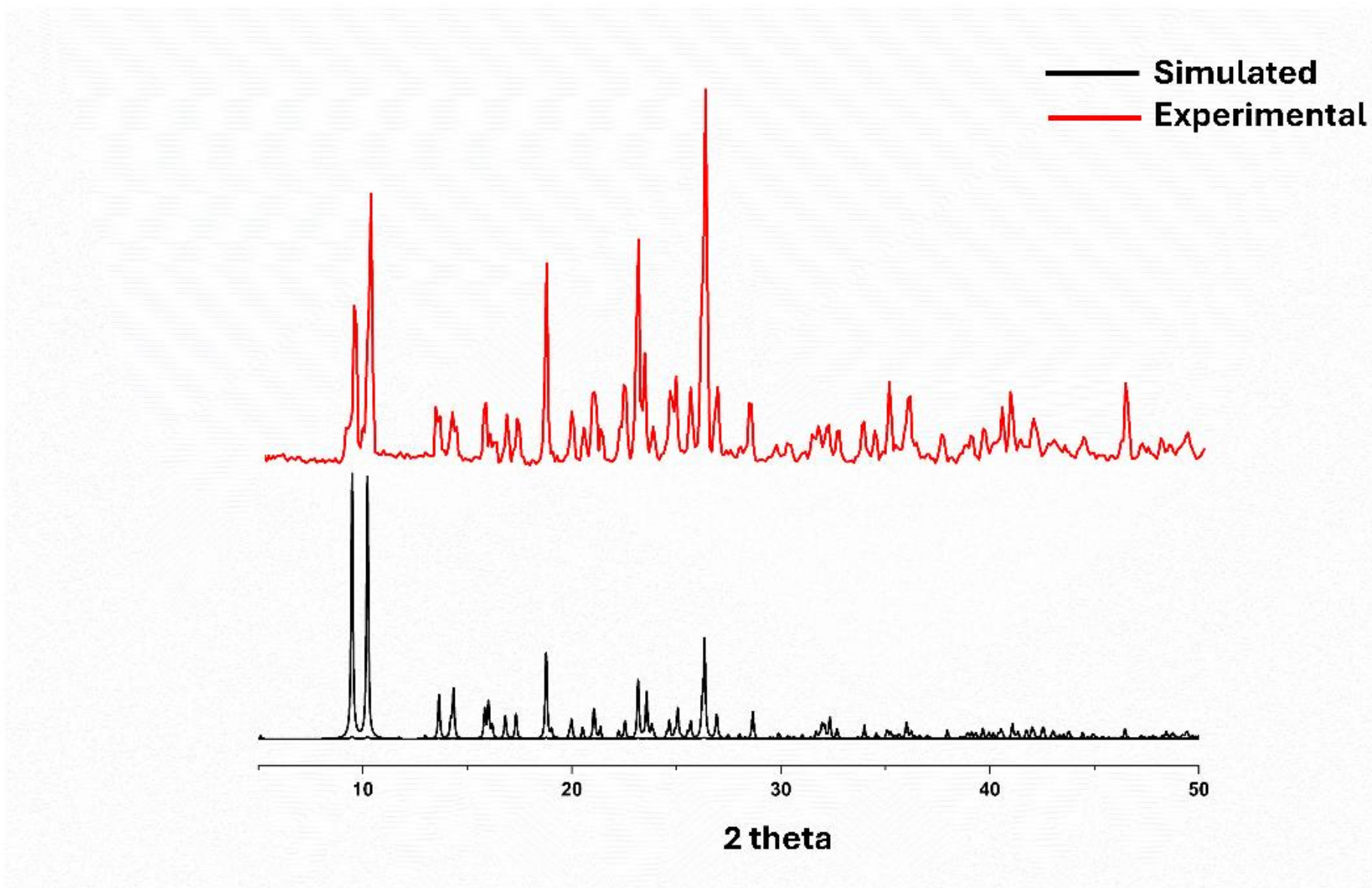

Figure S19. Experimental and simulated X-ray powder diffractograms for complex **1**.

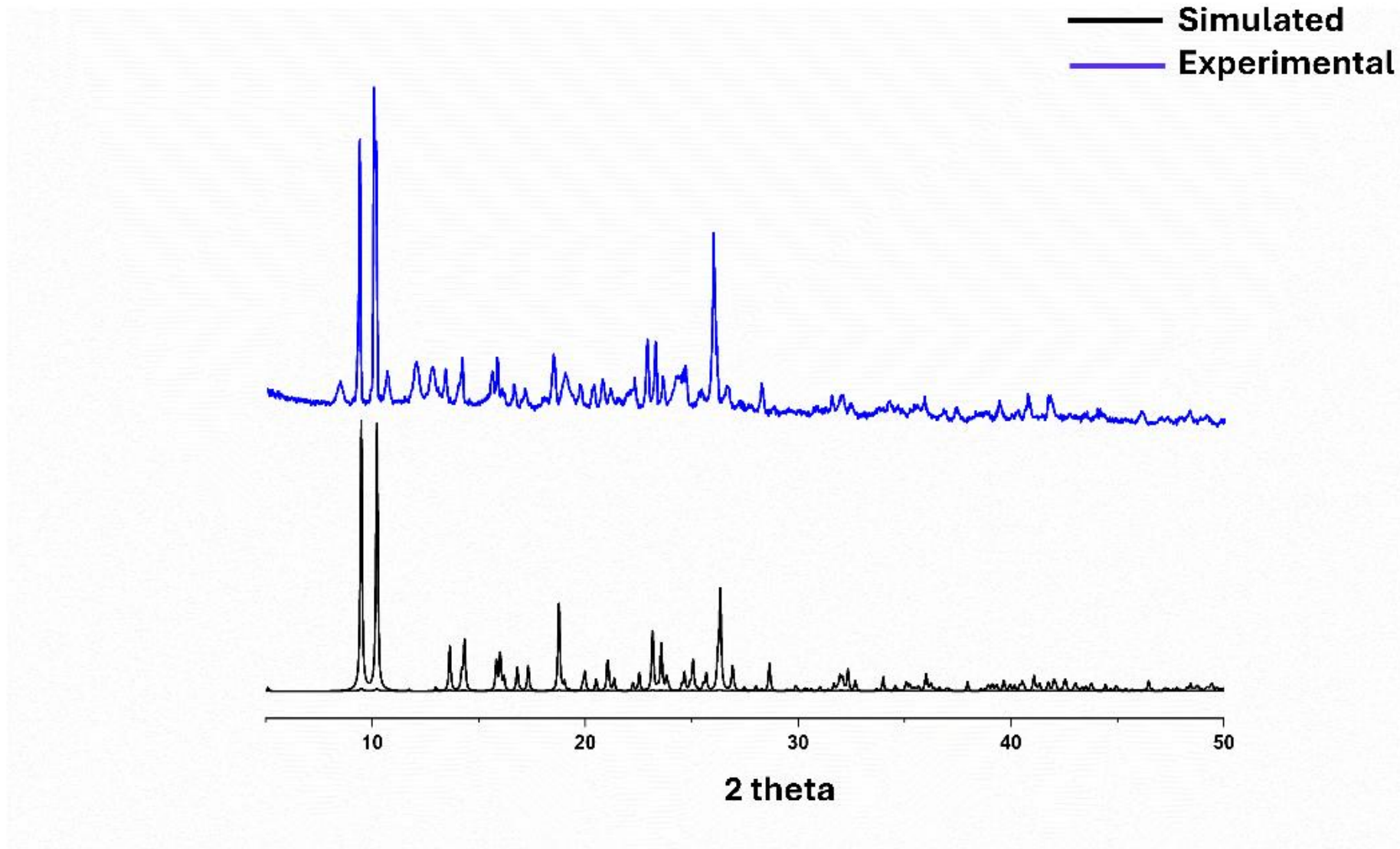

Figure S20. Experimental and simulated X-ray powder diffractograms for complex **2**.

Table S1. Crystal data and structure refinement for complex **1** and **2**.

	**Complex 1**	**Complex 2**
Empirical formula	$C_{30}H_{28}Cl_4Cu_2N_4O_2$	$C_{30}H_{28}Cl_4Cu_2N_4O_2$
Formula weight	745.46	745.46
Crystal system	Monoclinic	Monoclinic
Space group	$P2_1$	$P2_1$
a (Å)	7.67	7.68
b (Å)	11.06	11.07
c (Å)	17.61	17.61
α (°)	90	90
β (°)	100.72	100.72
γ (°)	90	90
Volume (Å^3)	1469.90	1472.50
Z	2	2
Density (calculated)	1.684 Mg/m^3	1.681 Mg/m^3
Absorption coefficient	5.431 mm^{-1}	5.421 mm^{-1}
F (000)	756	756
Theta range for data collection	5.111 to 76.116°	2.553 to 76.261°
Reflections collected	29114	106079
Independent reflections	5546 [R(int) = 0.1088]	6046 [R(int) = 0.0370]
Completeness to theta = 67.684°	99.5 %	99.9 %
Goodness-of-fit on F^2	1.092	1.037
Final R indices ($I > 2\sigma(I)$)	R1 = 0.0371, wR2 = 0.1047	R1 = 0.0251, wR2 = 0.0691
R indices (all data)	R1 = 0.0372, wR2 = 0.1048	R1 = 0.0252, wR2 = 0.0692
Absolute structure parameter	0.000(16)	0.003(10)
Largest diff. peak and hole	0.668 and -1.027 e.Å^{-3}	0.463 and -0.449 e.Å^{-3}

Table S2. Selected Bond Length (Å) and Bond angle (°) of complex **1** and **2.**

Bond Length(Å)				Bond angle (°)			
**Complex 1**		**Complex 2**		**Complex 1**		**Complex 2**	
Cu(1) -N(1A)	2.014	Cu(1) -N(1A)	2.020	N1A-Cu1-N2A	81.03	N1A-Cu1-N2A	81.22
Cu(1)-N(2A)	2.023	Cu(1)-N(2A)	2.028	N1A-Cu1-Cl2B	90.13	N1A-Cu1-Cl2B	90.20
Cu(1)-Cl(1A)	2.254	Cu(1)-Cl(1A)	2.253	N2A-Cu1-Cl2A	91.54	N2A-Cu1-Cl2A	91.41
Cu(1)-Cl(2A)	2.258	Cu(1)-Cl(2A)	2.258	Cl2A-Cu1-Cl2B	87. 72	Cl2A-Cu1-Cl2B	87.75
Cu(1)-Cl(2B)	2.909	Cu(1)-Cl(2B)	2.905	N1B-Cu2-N2B	81.06	N1B-Cu2-N2B	81.13
Cu(2) -N(1B)	2.033	Cu(2) -N(1B)	2.037	N2B-Cu2-Cl2B	88.13	N2B-Cu2-Cl2B	88.15
Cu(2)-N(2B)	2.034	Cu(2)-N(2B)	2.037	N1B-Cu1-Cl2A	102.40	N1B-Cu1-Cl2A	102.47
Cu(2)-Cl(1B)	2.270	Cu(2)-Cl(1B)	2.271	Cl2A-Cu2-Cl2B	92.72	Cl2A-Cu2-Cl2B	92.65
Cu(2)-Cl(2A)	2.682	Cu(2)-Cl(2A)	2.684				
Cu(2)-Cl(2B)	2.293	Cu(2)-Cl(2B)	2.292				

Table S3. Hydrogen bond distance and angles (°) for complex **1**.

Type	Donor	H···Acceptor	D–H	H···A	D···A	∠D–H···A
**Inter**	N2B–H1	Cl1A[b]	0.81	2.50	3.246	153
**Inter**	C2B–H2B	O1B[e]	0.95	2.50	3.432	165
**Inter**	C15A–H15A	Cl1B[a]	1.00	2.72	3.598	147

Table S4. C-H··· π bond distance and angles (°) for complex **1**.

	X···H	Cg	H···Cg	X···Cg	∠X-H···Cg
**C-H···Cg[d]**	C2A -H2A	Cg11	2.79	3.544	136

Table S5. π …π bond distance and angles (°) for complex **1**.

Type	Cg(I) ···Cg(I)	Cg···Cg	α	β	Γ
**π···π[c]**	Cg(9)…Cg(10)	4.014	15.6	10	25.5

Symmetry code: (a)-1+x,y,z (b)1+x,y,z (c)1-x,-1/2+y,-z (d)1-x,1/2+y,1-z (e)2-x,1/2+y,-z, Centroids, Cg(11) = C_{9B}–> C_{10B}–> C_{11B} –> C_{12B} –> C_{13B} –> C_{14B} ring, Cg(9) = N1B-->C1B-->C2B-->C3B-->C4B-->C5B ring and Cg(10) = C9A-->C10A-->C11A-->C12A-->C13A-->C14A ring

Table S6. Results of Continuous Shape Measurement Analysis of complex **1** for the Cu^{II} Coordination Spheres[a]._The lowest CShMs value which corresponds to closest geometry is highlighted.

5-vertex polyhedral for complex **1**				
	**Cu1**	**Cu2**	**Symmetry**	**Ideal Shape**
PP-5	29.40287	31.57231	D_{5h}	Pentagon
vOC-5	3.22818	2.20042	C_{4v}	Vacant octahedron (Johnson square pyramid, J1)
TBPY-5	5.38473	5.38473	D_{3h}	Trigonal bipyramid
**SPY-5**	**2.28607**	**1.06699**	$\mathbf{C_{4v}}$	**Square pyramid**
JTBPY-5	8.99572	8.71546	D_{3h}	Johnson trigonal bipyramid (J12)

[a]CShM values between 0.1 and 3 usually correspond to a not negligible but still small distortion from ideal geometry

2. *Catalytic activity and Kinetic studies*

Electrochemical Measurements: All electrochemical measurements were carried out using a three-electrode system using a *CHI 7044E* potentiostat. A glassy carbon electrode (GCE) with a geometrical area of 0.07 cm^2 served as the working electrode, with a platinum wire as the counter electrode and an Ag/AgCl (sat. KCl) electrode as the reference. For CV taken in methanol, 0.1 M tetrabutylammonium hexafluorophosphate (TBAF) was used as a supporting electrolyte. For OER studies, a 0.1 M phosphate buffer solution (PBS) with adjusted pH was used, with 0.1 M Na_2SO_4 as a supporting electrolyte. The Shimadzu UV-3600 I Plus spectrophotometer was used for UV-Vis spectral studies.

All the potentials were converted to the normal hydrogen electrode (NHE) scale:

$$E_{NHE}\ (V) = E_{Ag/AgCl} + 0.197 \qquad (Equation\ S1)$$

or to a reversible hydrogen electrode (RHE) scale using the Nernst equation at room temperature:

$$E_{RHE}\ (V) = E_{Ag/AgCl} + 0.0591 \times pH + 0.197 \qquad (Equation\ S2)$$

Constant potential electrolysis was performed at 1.8 V (*vs.* RHE) at room temperature and constant stirring in an undivided cell with a 1×1 cm^2 carbon-cloth as the working electrode, Pt as counter electrode and Ag/AgCl as reference.

% Faradaic yield (FY) was determined from the following equations[1, 2]:

$$FE\ (\%) = \frac{\text{moles of product formed} \times 96{,}485\ (C\ mol^{-1}) \times n}{\text{total charge passed (C)}} \times 100\ \% \qquad (Equation\ S3)$$

where, n = is the number of moles of electrons transferred per mole of product (n = 4 for OER)

The amount of produced hydrogen and oxygen in the reactor headspace was analyzed using a gas chromatograph (CIC-Dhruva) equipped with a thermal conductivity detector (TCD).[3-4] The moles were calculated according to the method reported in the literature.[5-6]

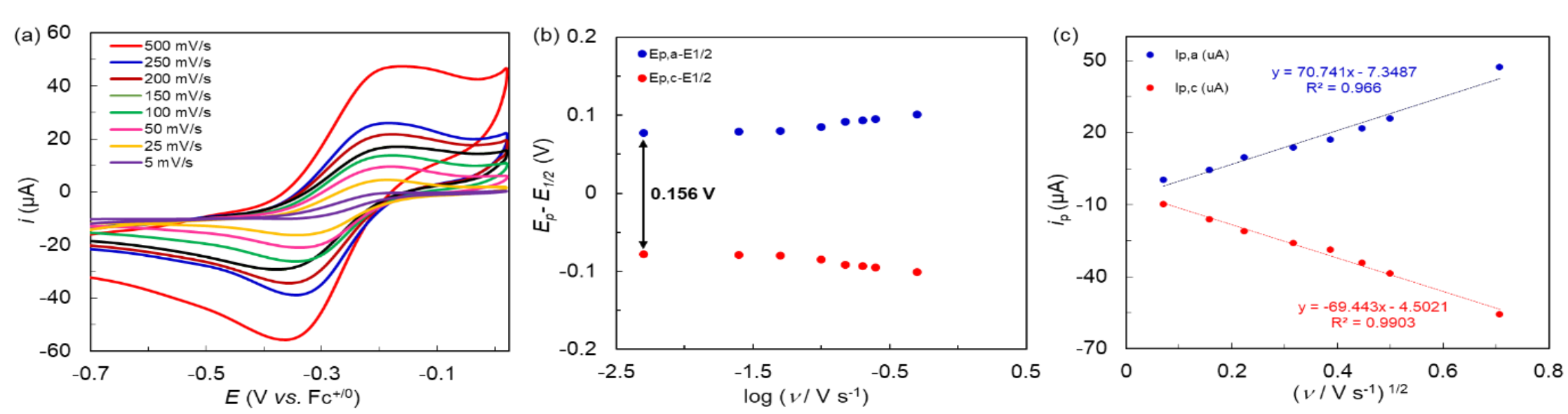

Figure S21. (a) Scan rate dependent CV of redox couple 1 of complex **1** (1 mM) in methanol; (b) corresponding $E_p - E_{1/2}$ *vs.* log (scan rate) plot. (c) corresponding i_p *vs.* (scan rate)$^{1/2}$ plot. Experimental conditions: 100 mM TBAF as supporting electrolyte in methanol; glassy carbon, Pt

wire, and Ag/AgCl were used as the working, counter, and reference electrodes, respectively; 298 K; the potential is adjusted with respect to $Fc^{+/0}$.

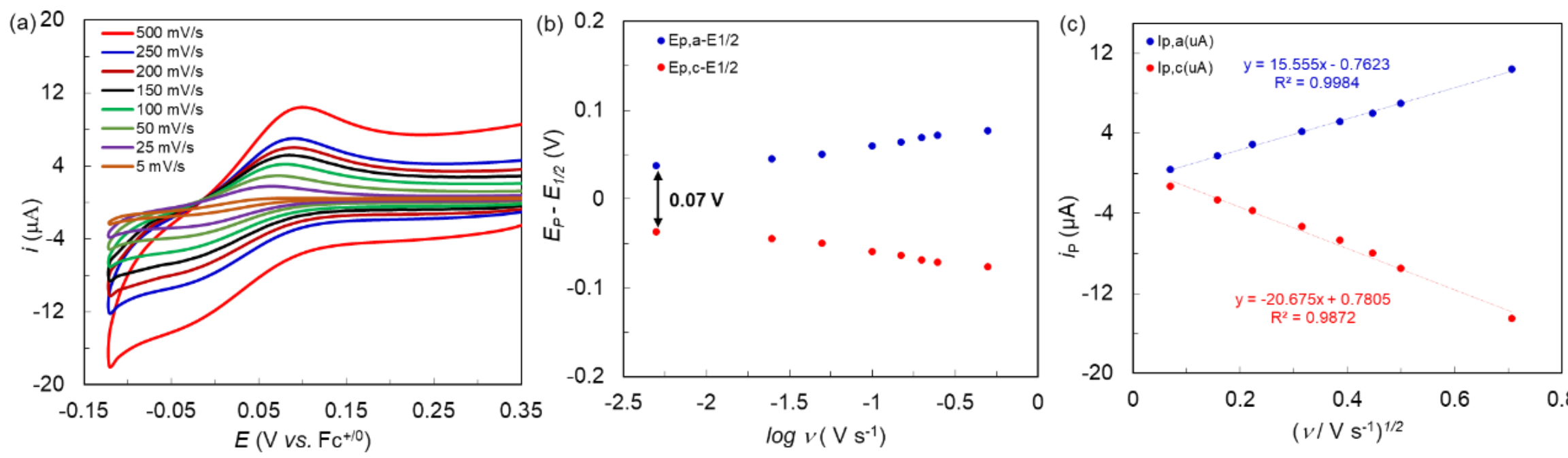

Figure S22. (a) Scan rate dependent CV of redox couple 2 of complex **1** (1 mM) in methanol; (b) corresponding $E_p - E_{1/2}$ *vs.* log (scan rate) plot. (c) corresponding i_p *vs.* (scan rate)$^{1/2}$ plot. Experimental conditions: 100 mM TBAF as supporting electrolyte in methanol; glassy carbon, Pt wire, and Ag/AgCl were used as the working, counter, and reference electrodes, respectively; 298 K; the potential is adjusted with respect to $Fc^{+/0}$.

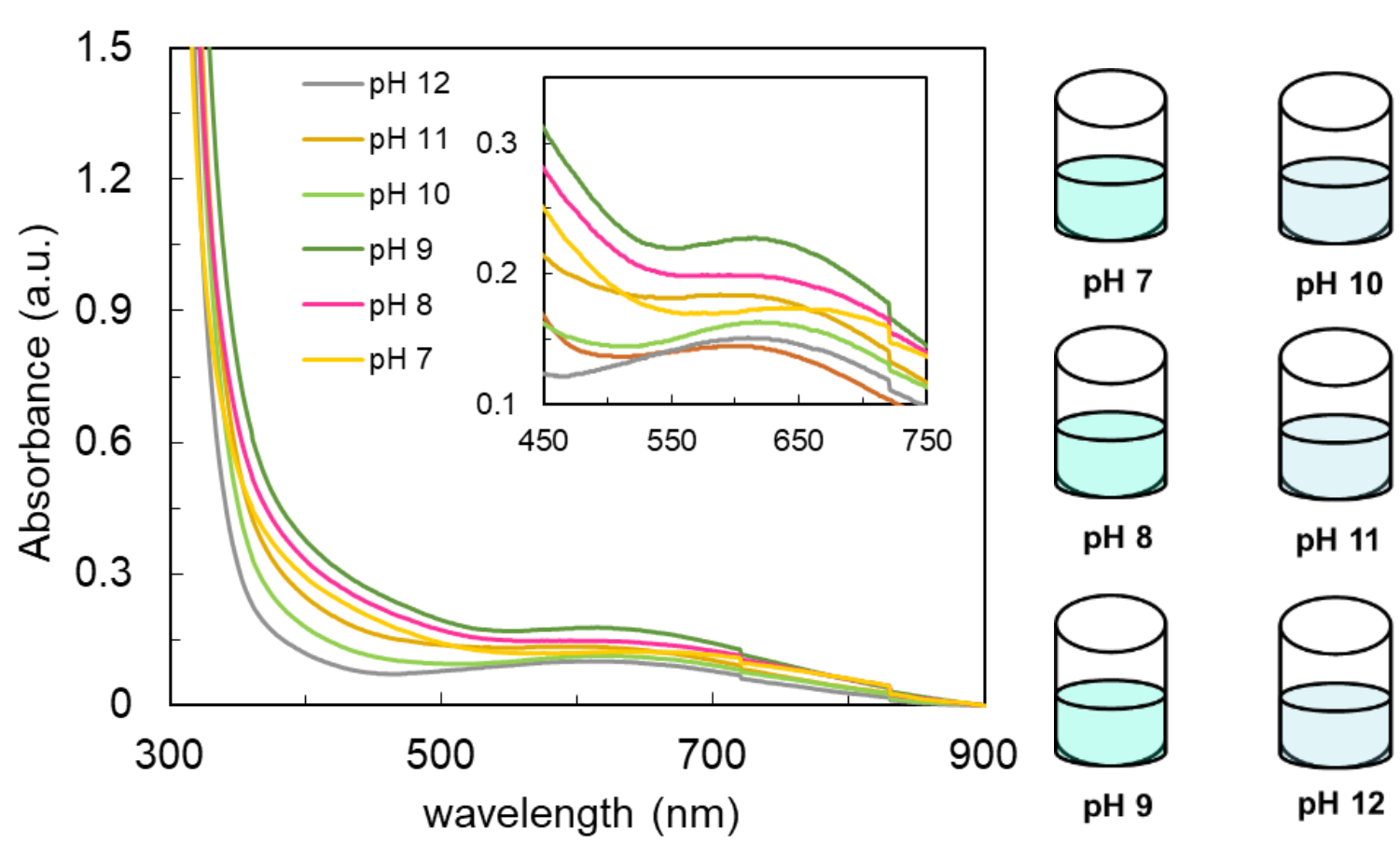

Figure S23. UV-vis spectra of complex **1** (1 mM) in 0.1 M PBS at varied pH values of 7 to 12.

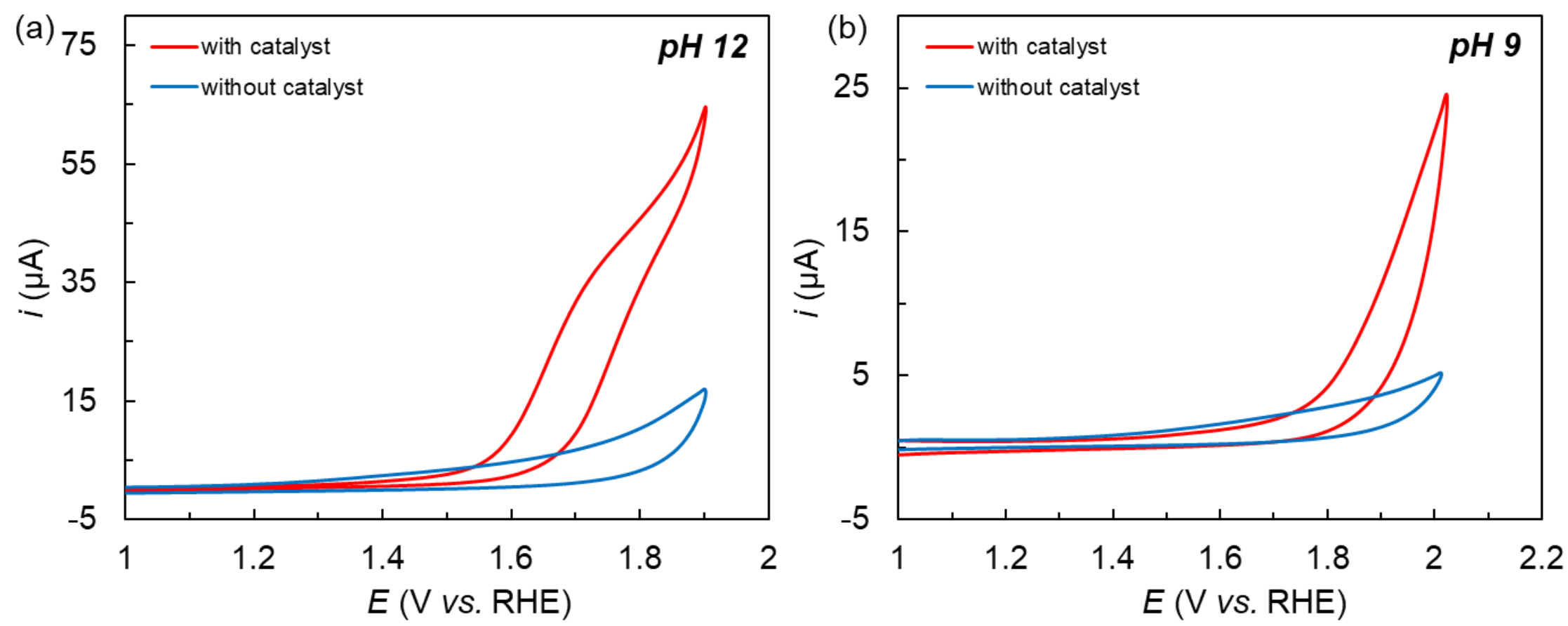

Figure S24. Comparison CVs of OER with and without catalyst (1 mM complex **1**) in (a) pH 12 and (b) pH 9. Experimental conditions: 0.1 M PBS with 0.1 M Na_2SO_4 as supporting electrolyte; glassy carbon, Pt wire, and Ag/AgCl were used as the working, counter, and reference electrodes, respectively; 298 K; the potential is adjusted with respect to RHE.

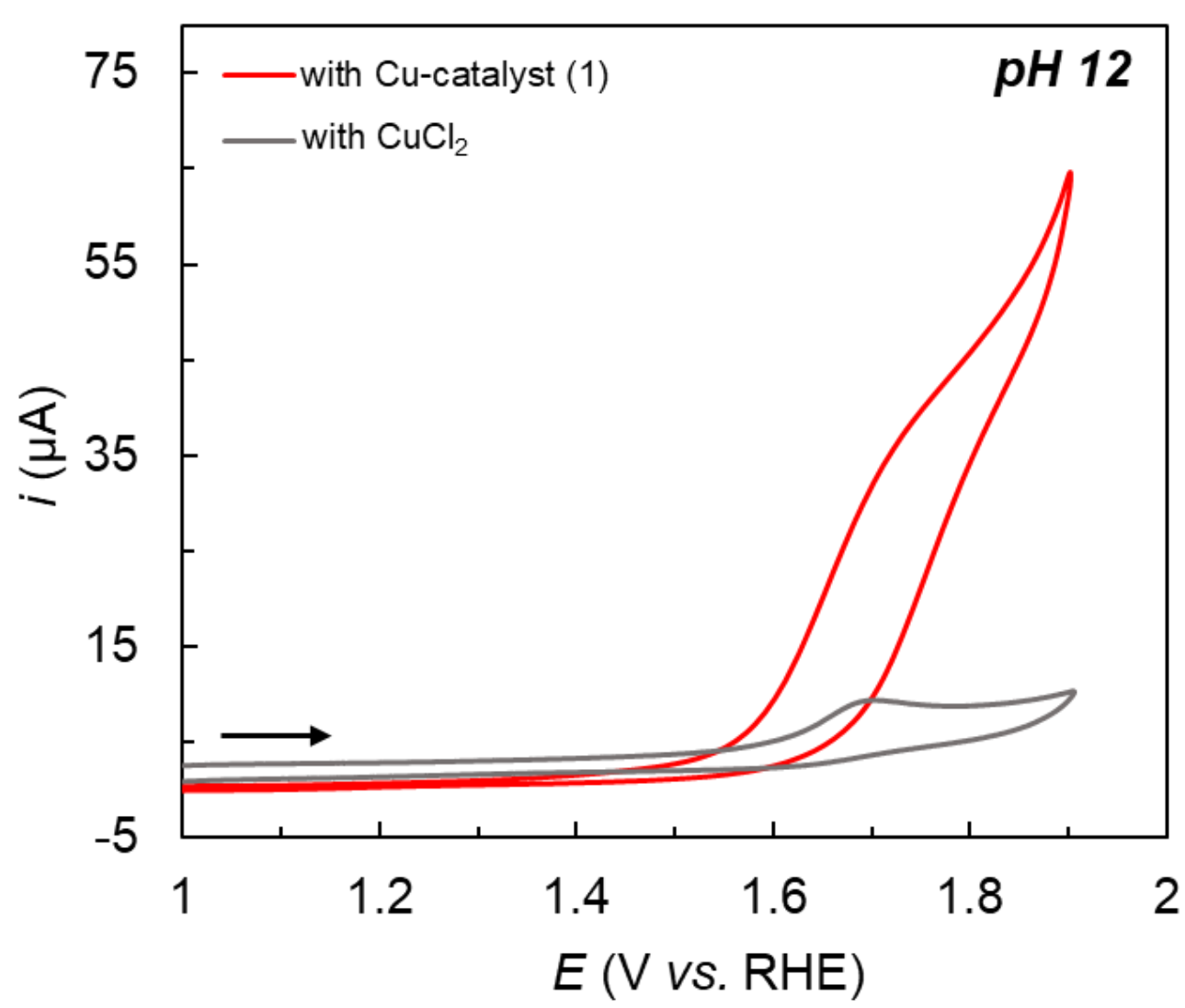

Figure S25. Comparison of CVs of OER with catalyst (1 mM complex **1**) and $CuCl_2$ (1 mM) in pH 12. Experimental conditions: 0.1 M PBS (adjusted to pH 12) with 0.1 M Na_2SO_4 as supporting electrolyte; glassy carbon, Pt wire, and Ag/AgCl were used as the working, counter, and reference electrodes, respectively; 298 K; the potential is adjusted with respect to RHE.

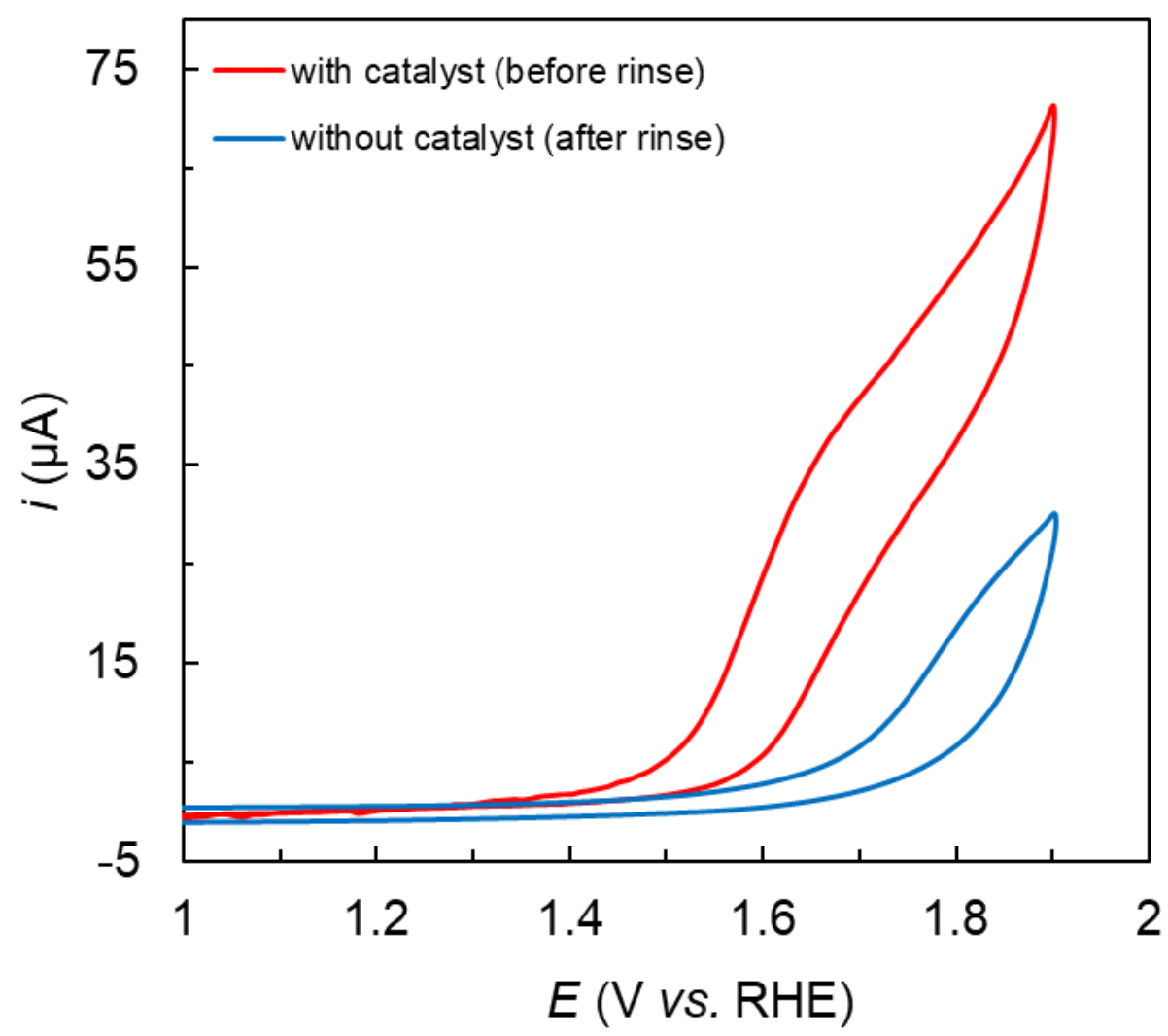

Figure S26. Electrocatalytic OER performance after rinse test in pH 12: (red) CV with catalyst (1 mM complex **1**) before rinse test and (blue) without catalyst using the same glassy carbon after rinsing with water. Experimental conditions: 0.1 M PBS with 0.1 M Na_2SO_4 as supporting electrolyte; glassy carbon, Pt wire, and Ag/AgCl were used as the working, counter, and reference electrodes, respectively; 298 K; the potential is adjusted with respect to RHE.

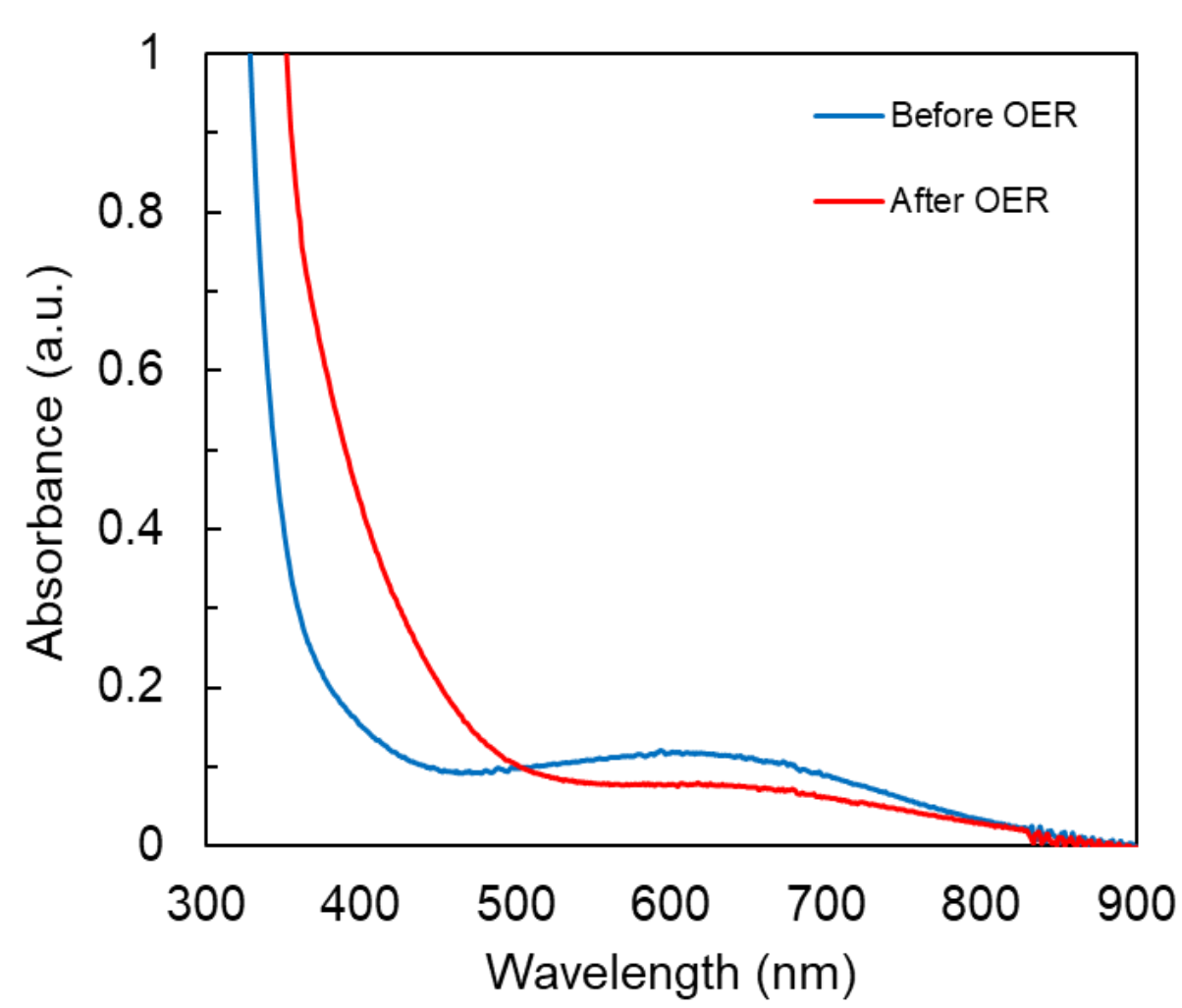

Figure S27. Pre- and post- OER electrolysis characterization of electrolyte using UV-vis spectra before electrolysis (blue) and after electrolysis (red).

Table S7. Summary of reported dinuclear copper catalysts for OER in comparison with our catalyst. *(Entries 2 and 8 are heterogeneous systems)*

S. No.	Catalyst	[Catalyst]	*η*	CPE condition (V *vs.* RHE)	Electrolyte	Faradaic Yield (%)	Ref.
1	$\{[(Me_2TMPA)Cu(II)]_2(\mu\text{-}OH)_2\}^{2+}$	1.0 mM	710 mV	2.36	0.1 M NaOTf (pH 12.5)	> 90	7
2	$[C_{32}H_{24}Cu_2F_4N_4O_4]$	1.5 mg/cm^2 (*Hetero*)	340 mV (10mA/cm^2)	-	1.0 M KOH (pH 13.5)	-	8
3	$Cu(Me_2oxpn)Cu(OH)_2$	5.7 mM	636 mV	2.04	0.25 M PBS (pH 10.4)	90	9
4	$[L1Cu_2(\mu\text{-}OH)](BF_4)_3$ (L1=N,N′-dimethyl-N,N′-bis{2-[bis(2-pyridinylmethyl)amino]ethyl}ethane-1,2-diamine)	1.0 mM	770 mV (1mA/cm^2)	2.21	0.1 M PBS (pH 12)	97	10
5	$[Cu_2(BPMAN)(\mu\text{-}OH)]^{3+}$	1.0 mM	800 mV	2.28	0.1 M PBS (pH 7.0)	98	11
6	$[Cu_2(6\text{-}HPA)(\mu\text{-}OH)]^{3+}$	1.0 mM	834 mV	2.28	0.1 M PBS (pH 7.0)	93	12
7	$[Cu(oxpn)Cu(OH)_2]$	2.2 µM	289 mV	1.99	0.25 M buffer (pH 10.1)	96.5	13
8	$CuPz\text{-}H_2O.H_2O$	0.05 mg/cm^2 (*Hetero*)	228 mV (10 mA/cm^2)	-	1.0 M KOH (pH 14)	-	14
9	$([Cu_2(TPMAN)(\mu\text{-}OH)(H_2O)]^{3+}$	1.0 mM	780 mV	2.28	0.1 M PBS (pH 7.0)	-	15
10	$[Cu^{II}_2(\mu_2\text{-}Cl)_2(Cl)_2(L_{1R2S})_2]$	1.0 mM	330 mV	1.80	0.1 M PBS (pH 12)	86.1±6.4	***This work***

Rationalization of Kinetic Parameters

According to Briggs -Haldane scheme, the formation and dissociation of enzyme substate complex ES is shown by: -

$$S + E \underset{-k_1}{\overset{k_1}{\rightleftharpoons}} ES \xrightarrow{k_2} P + E$$

By applying this scheme, the Michael Menten equation for the initial rection rate for the concentration measurement was obtained

$$V = V_{max}[S]/([S] + K_m) \qquad (\textit{Equation S4})$$

Plot of this equation show hyperbolic nature which ultimately saturated at higher concentration. This is also the general rule for the first order kinetics. The initial rate constants are obtained from the slope of log $[A_\alpha/(A_\alpha - A_t)]$ vs. t (time curve) which shows a straight line.

The above method is used to determine the kinetic parameters like V_{max} i.e. Maximum Reaction Velocity, K_m i.e. Michael Constant and k_{cat} i.e. Turnover Number.

Electrospray Ionization High-Resolution Mass Spectra: ESI-HRMS were recorded on Waters XEVO G3 – QTOF-HRMS instrument, dissolving the complex **1** and substrate in LC-MS quality MeOH with 1 : 50 ratio.

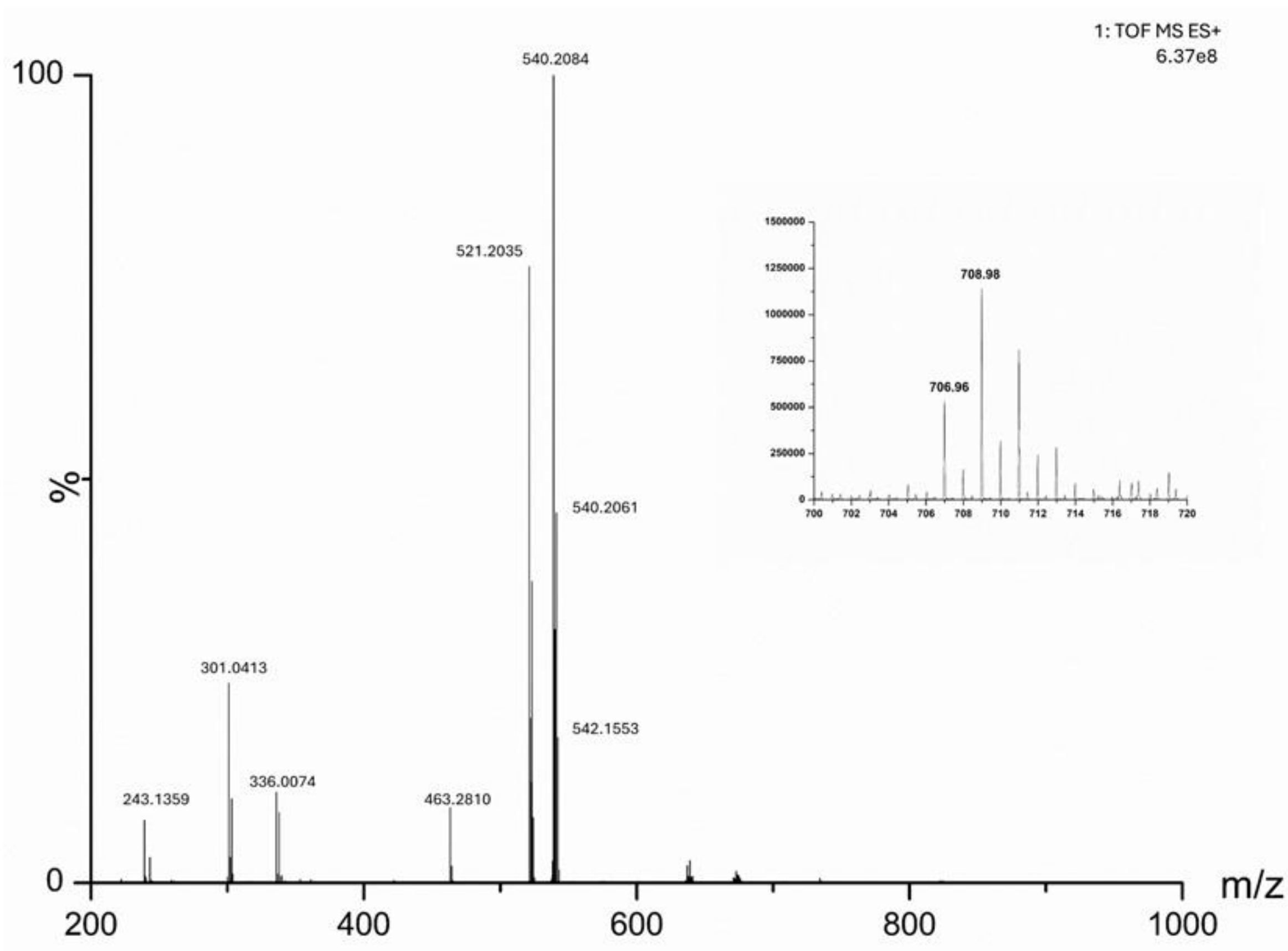

Figure S28. HRMS of complex **1** in the presence of substrate (3,5 DTBC) in MeOH (inset : magnified view of m/z 700-720 showing binuclear species $[Cu_2(Cl_3)(L_2)]^+$) (Catalyst Conc. = 1×10^{-4} M, Substrate Conc. = 5×10^{-3} M).

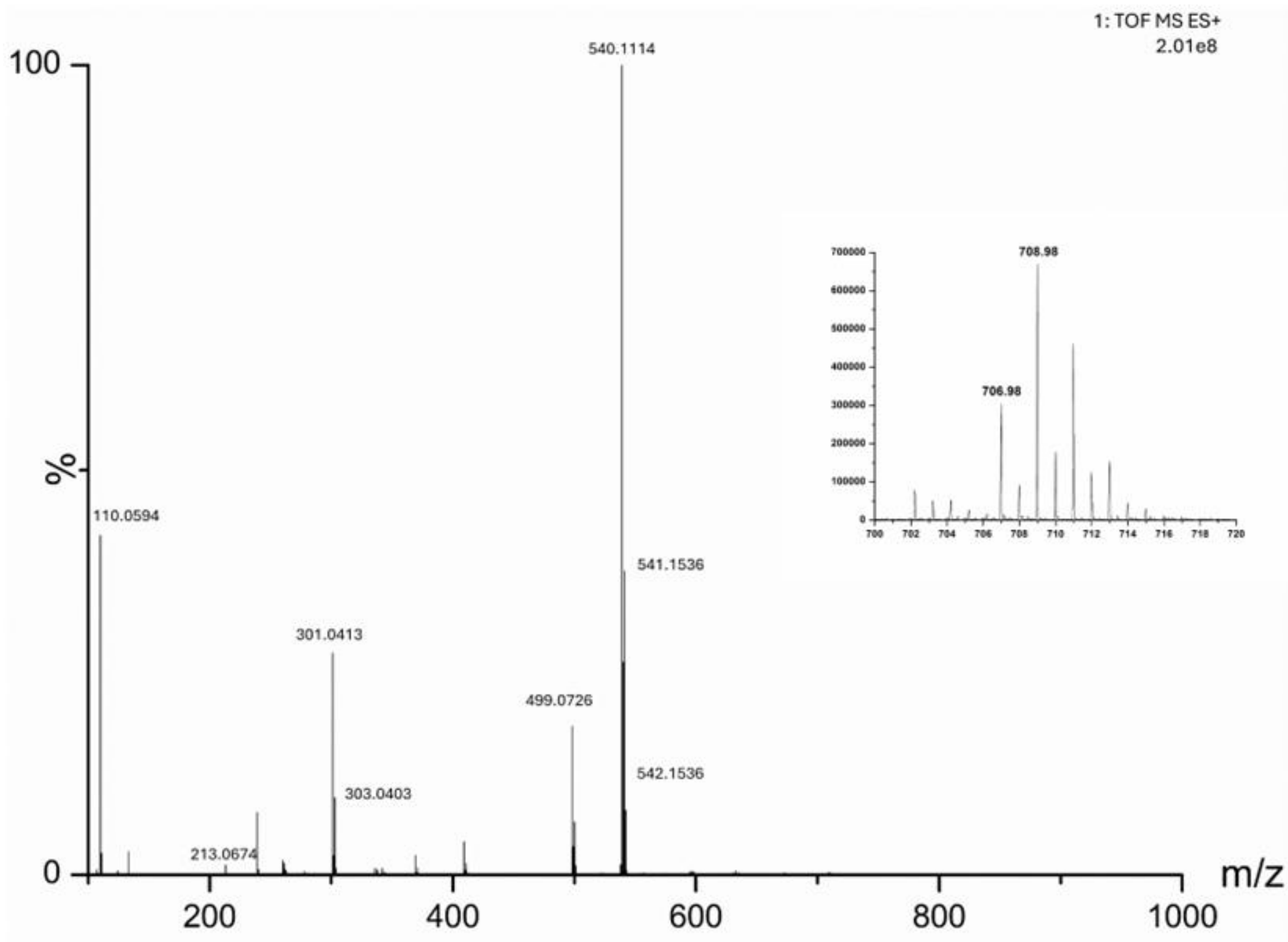

Figure S29. HRMS of complex **1** in the presence of substrate (OAPH) in MeOH (inset : magnified view of m/z 700-720 showing binuclear species $[Cu_2(Cl_3)(L_2)]^{+}$) (Catalyst Conc. = 5×10^{-5} M, Substrate Conc. = 2.5×10^{-3} M).

Detection of Hydrogen Peroxide (H_2O_2) in the Catalytic Reaction:-

To detect the H_2O_2 formation in the Catechol oxidase (CO) and Phenoxazinone synthase (PHS) mimicking activity, the reaction mixture was prepared as in the kinetic experiment. For 3,5 DTBC the substrate concentration was fixed at 1×10^{-2} M and catalyst concentration was 1×10^{-4} M while for OAPH the concentration of catalyst was 5×10^{-5} M and for substrate, it was 5×10^{-3} M. After 1 hour the pH of the mixtures adjusted to 2 using H_2SO_4 and aqueous layer was extracted with Dichloromethane (DCM). 1 ml of 10 % Potassium Iodide (KI) and 3-4 drops of 3 % ammonium

molybdate were added. In the presence of H_2O_2 and excess iodide ions the triiodide ion (I_3^-) is formed.

$$H_2O_2 + 2I^- + 2H^+ \longrightarrow 2H_2O + I_2 \qquad (Equation\ S5)$$

$$I_{2(aq)} + I^- \longrightarrow I_3^- \qquad (Equation\ S6)$$

The formation of the I_3^- monitored spectrophotometrically (λ = 353 nm, ε = 26,000 M^{-1} cm^{-1}) for both the reactions. The characteristics band at 353 nm for both the reactions confirms that H_2O_2 is generated in the enzyme mimicking reactions.[16-22]

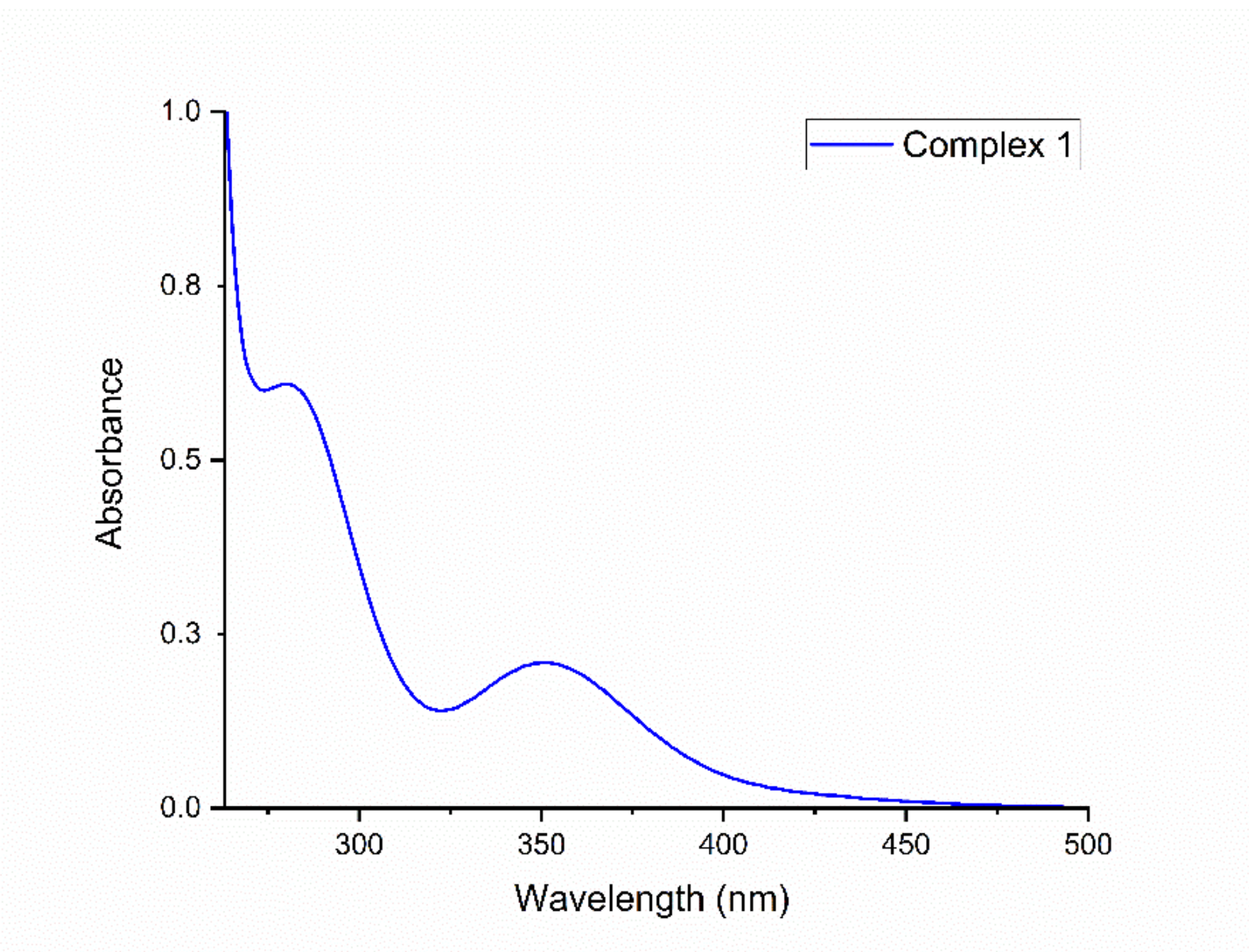

Figure S30. Iodometric Detection of H_2O_2 formation during oxidation of 3,5-DTBC observed spectrophotometrically at Room Temperature.

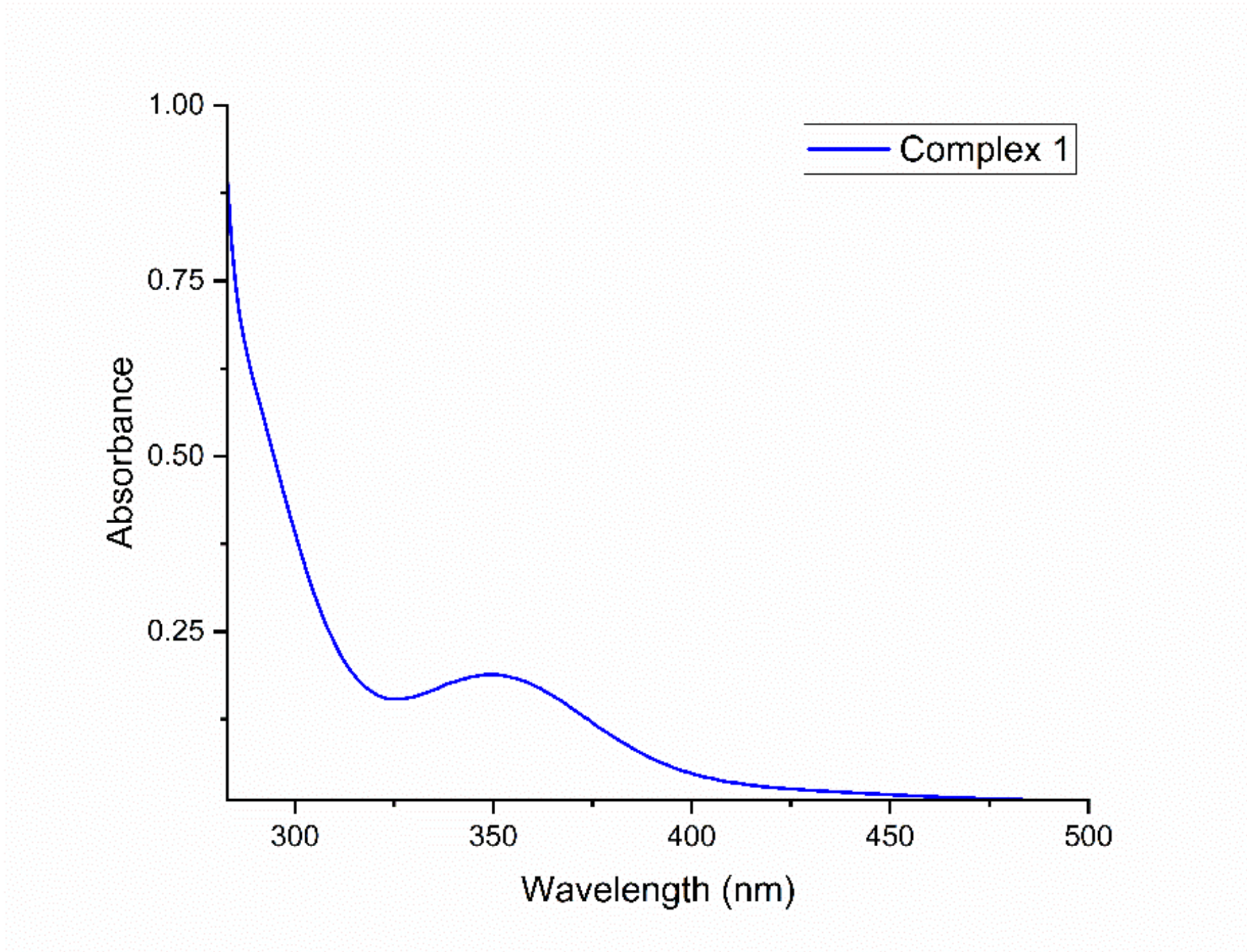

Figure S31. Iodometric Detection of H_2O_2 formation during oxidation of OAPH observed spectrophotometrically at Room Temperature.

Comparison Table

Table S8. Catalytic Activities (k_{cat}) of different Cu (II) based complexes Catechol Oxidase like Enzymatic Activity

S.No.	Molecular Formula	Solvent	Turnover Number (k_{cat}) h^{-1}	Binding Constant (K_M) (M)	Max. Velocity (V_{max}) (Ms^{-1})	Ref.
1.	$[CuL^{32}{}_2]_2$	**EtOH**	**5.74×10^{4}**	**9.1×10^{-2}**	**4.7×10^{-2}**	23
		**MeOH**	**5.38×10^{4}**	**4.18×10^{-2}**	**4.4×10^{-2}**	
2.	$[Cu(L^{33})Cl_2]$	**MeOH**	**6.12×10^{2}**	**1.77×10^{-3}**	**-**	16
3.	$[Cu(L^{34})Cl_2]$	**MeOH**	**3.43×10^{2}**	**1.195×10^{-3}**	**-**	

**4.**	$[Cu(L^{35})Cl]$	**MeOH**	**2.519×10^{1}**	**1.733×10^{-4}**	**6.99×10^{-8}**	24
**5.**	$[Cu(L^{36})Cl]_2$	**MeOH**	**1.076×10^{1}**	**1.01×10^{-3}**	**2.99×10^{-8}**	
**6.**	$[Cu(L^{32})(N(CN)_2)]_2.2H_2O$	**MeOH**	**1.06×10^{5}**	**6.78×10^{-2}**	**8.8×10^{-2}**	23
		**EtOH**	**5.96×10^{4}**	**5.23×10^{-2}**	**4.9×10^{-2}**	
		**ACN**	**8.93×10^{4}**	**1.05×10^{-1}**	**7.4×10^{-2}**	
		**DMF**	**4.19×10^{5}**	**3.44×10^{-1}**	**3.43×10^{-1}**	
**7.**	$[Cu(L^{37})(phen)](ClO_4)$	**Buffer**	**1.6×10^{6}**	**16.9×10^{-4}**	**14×10^{-4}**	25
**8.**	$[Cu(L^{38})(phen)](ClO_4)$	**Buffer**	**1.5×10^{6}**	**12×10^{-4}**	**12.1×10^{-4}**	25
		**MeOH**	**2.6×10^{3}**	**1.2×10^{-4}**	**3.367×10^{-5}**	
**9.**	$[Cu(L^{37})(phen)](ClO_4)$	**MeOH**	**1.7×10^{3}**	**0.4×10^{-4}**	**1.4×10^{-5}**	25
**10.**	$[Cu_2(L^{39})(\mu\text{-}OAc)(ClO_4)]ClO_4$	**MeOH-H2O**	**3.2684×10^{2}**	**8.1×10^{-3}**	**-**	26
**11.**	$[(CuL^{14})_2(PhCOO)]ClO_4$	**ACN**	**2.448×10^{2}**	**2.79×10^{-3}**	**6.8×10^{-7}**	27
**12.**	$[Cu_2(L^{40})(\mu\text{-}OAc)(ClO_4)]ClO_4$	**MeOH-H2O**	**2.3115×10^{2}**	**2.81×10^{-3}**	**-**	26
**13.**	$[Cu_2(L^{41})(\mu\text{-}OAc)(CH_3OH)(ClO_4)]ClO_4$	**MeOH-H2O**	**5.112×10^{1}**	**0.58×10^{-3}**	**-**	
**14.**	$[Cu(L^{31})(NCS)(ClO_4)]$	**EtOAc**	**5×10^{1}**	**2.8×10^{-2}**	**1.38×10^{-6}**	28
**15.**	$[Cu_2Ac_2O(L^{42})ClO_4]$	**MeOH**	**3.88×10^{1}**	**2.2302×10^{-4}**	**1.617×10^{-6}**	29
		**NPA**	**2.52×10^{1}**	**2.1530×10^{-4}**	**1.051×10^{-6}**	

**16.**	$[Cu(L^{31})(NCS)(ClO_4)]$	**MeOH**	**2.52×10^{1}**	**2.5×10^{-3}**	**7×10^{-7}**	28
**17.**	$[Cu_2Ac_2O(L^{42})ClO_4]$	**DMSO**	**2.51×10^{1}**	**2.1595×10^{-4}**	**1.047×10^{-6}**	29
**18.**	$[Cu_2(L^{42})(OH)(H_2O)(EtOH)][ClO_4]_2$	**MeOH**	**2.35×10^{1}**	**2.7293×10^{-4}**	**9.81×10^{-7}**	
**19.**	$[Cu_2Ac_2O(L^{42})ClO_4]$	**ACN**	**2.22×10^{1}**	**2.155×10^{-4}**	**9.26×10^{-7}**	
**20.**	$Cu(ClO_4)_2(L^{43})$	**ACN**	**2×10^{1}**	**1.61×10^{-3}**	**1.689×10^{-6}**	
**21.**	$[Cu_3(L^{44})_3(\mu 3\text{-}CO_3)](ClO_4)_3$	**MeOH**	**2.124×10^{1}**	**2.36×10^{-3}**	**5.90×10^{-8}**	30
**22.**	$[Cu_2(L^{42})(OH)(H_2O)(EtOH)][ClO_4]_2$	**DMF**	**1.76×10^{1}**	**2.2981×10^{-4}**	**7.33×10^{-6}**	29
**23.**	$[Cu_2(\mu\text{-}L^{45})(\mu\text{-}OH)](ClO_4)_2$	**MeOH**	**1.704×10^{1}**	**2.18×10^{-3}**	**4.73×10^{-8}**	31
**24.**	$[Cu_3(L^{46})_3(\mu 3\text{-}CO_3)](ClO_4)_3$	**MeOH**	**1.659×10^{1}**	**1.91×10^{-3}**	**4.61×10^{-8}**	
**25.**	$[Cu_2(\mu\text{-}L^{45})(\mu\text{-}OMe)](ClO_4)_4\cdot 2H_2O$	**MeOH**	**8.93×10^{0}**	**2.57×10^{-3}**	**2.48×10^{-8}**	31
**26.**	$[Cu(L^{32})NCS]_2.2CH_3OH$	**MeOH**	**1.18×10^{5}**	**8.2×10^{-2}**	**9.8×10^{-2}**	23
**27.**	**$Cu_2(\mu_2\text{-}Cl)_2(Cl)_2(L)_2$**	**MeOH**	**$1.9 \pm 0.4 \times10^{3}$**	**$7 \pm 1.5 *10^{-2}$**	**$5 \pm 1.1 *10^{-5}$**	**This Work**

Table S9. Catalytic Activities (k_{cat}) of different Cu (II) based complexes Phenoxazinone Synthase like Enzymatic Activity

S.No.	Molecular Formula	Solvent	Turnover Number (k_{cat}) h^{-1}	Binding Constant (K_M) (M)	Max. Velocity (V_{max}) (Ms^{-1})	Ref.
**1.**	$[Cu(L^1)]$	**ACN**	**5.36×10^2**	**9.46×10^{-4}**	**1.49×10^{-5}**	33
**2.**	$[Cu_4(L^2)_4]$	**MeOH**	**1.272×10^1**	**1.73×10^{-3}**	**3.54×10^{-8}**	34
**3.**	$[Cu_2(L^3)_2]$	**MeOH**	**1.432×10^1**	**1.24×10^{-3}**	**3.98×10^{-8}**	34
**4.**	$[Cu(L^{4R})Cl]$	**MeOH -DMF**	**3.22×10^6**	**13.5×10^{-1}**	**8.9×10^{-2}**	35
**5.**	$[Cu(L^{4R})Br]$	**MeOH -DMF**	**1.8×10^6**	**7.4×10^{-1}**	**5×10^{-2}**	35
**6.**	$[Cu(L^5)Cl]$	**MeOH -DMF**	**3.8×10^5**	**11.9×10^{-1}**	**1.05×10^{-2}**	35
**7.**	$[Cu(L^5)Br]$	**MeOH -DMF**	**2.6×10^5**	**3.3×10^{-1}**	**7.2×10^{-3}**	35
**8.**	$[Cu(L^6)_2Cl_2]_2$	**MeOH**	**2.31×10^4**	**2.63×10^{-3}**	**6.44×10^{-4}**	36
**9.**	$[Cu(L^7)Cl_2].H_2O$	**MeOH**	**1.92×10^4**	**1.46×10^{-3}**	**5.35×10^{-4}**	37
**10.**	$[Cu(L^8)Cl2]$	**MeOH**	**5.2555×10^2**	**1.036×10^{-2}**	**-**	16
**11.**	$[Cu(L^9)Cl_2]$	**MeOH**	**2.5532×10^2**	**2.83×10^{-3}**	**-**	
**12.**	$[Cu(L^{10})(Cl)_2]$	**MeOH -H_2O**	**8.424**	**5.33×10^{-5}**	**5.85×10^{-8}**	38
**13.**	$[Cu(L^{11})(Cl)_2].H_2O$ (2.H_2O)	**MeOH -H_2O**	**7.668**	**1.32×10^{-4}**	**5.33×10^{-8}**	38
**14.**	$[Cu(L^{12})(Cl)_2]$.MeOH(1.MeOH)	**MeOH -H_2O**	**6.264**	**1.46×10^{-4}**	**4.35×10^{-8}**	38

**15.**	$[Cu(L^{6})_2(NO_3)_2]_2$	**MeOH**	**6.3×10^{4}**	**4.18×10^{-3}**	**1.75×10^{-3}**	36
**16.**	$[Cu_4(L^{13}))_2(L^{13})_2](Cl)(NO_3)\cdot 5H_2O$	**MeOH**	**1.861×10^{1}**	**8.67×10^{-4}**	**5.17×10^{-8}**	34
**17.**	$[Cu(L^{14})(NO_3)]$	**ACN**	**1.82×10^{-1}**	**1.95×10^{-3}**	**1.52×10^{-7}**	39
**18.**	$[Cu(L^{15})_2](ClO_4)_2$	**MeOH**	**7.20×10^{2}**	**-**	**2×10^{-5}**	40
		**ACN**	**3.60×10^{2}**	**-**	**1×10^{-5}**	
**19.**	$[Cu_2(L^{16})_3]ClO_4$	**ACN**	**7.81×10^{1}**	**4.4×10^{-4}**	**2.17×10^{-6}**	17
**20.**	$[Cu_3(L^{17})_3(\mu 3\text{-}CO_3)](ClO_4)_3$	**MeOH**	**3.966×10^{1}**	**2.92×10^{-3}**	**1.10×10^{-7}**	30
**21.**	$[Cu_2(\mu\text{-}L^{18})(\mu\text{-}OMe)](ClO_4)_4\cdot 2H_2O$	**MeOH**	**3.107×10^{1}**	**1.97×10^{-3}**	**8.63×10^{-8}**	31
**22.**	$[Cu_3(L^{19})_3(\mu 3\text{-}CO_3)](ClO_4)_3$	**MeOH**	**2.891×10^{1}**	**2.79×10^{-3}**	**8.03×10^{-8}**	30
**23.**	$[Cu_2(\mu\text{-}L^{18})(\mu\text{-}OMe)](ClO_4)_4\cdot 2H_2O$	**MeOH**	**1.824×10^{1}**	**2.19×10^{-3}**	**5.06×10^{-8}**	31
**24.**	$[(CH_3CN)Cu(L^{20})_2Cu](ClO_4)_2$	**ACN**	**1.11×10^{1}**	**1.70×10^{-2}**	**1.11×10^{-3}**	32
**25.**	$[(CuL^{14})_2(PhCOO)]ClO_4$	**ACN**	**0.524×10^{1}**	**1.95×10^{-3}**	**8.74×10^{-7}**	27
**26.**	$[Cu(L^{21})(N_3)]$	**MeOH**	**3×10^{6}**	**7.3×10^{-1}**	**8.45×10^{-2}**	41
**27.**	$[Cu(L^{22R})(N)_3]$	**MeOH -DMF**	**9.3×10^{4}**	**1.9×10^{-1}**	**2.6×10^{-3}**	35
**28.**	$[L^{23}Cu(\mu_{1,1}\text{-}N_3)_2CuL23]$	**ACN**	**2.59×10^{4}**	**1.66×10^{-2}**	**7.2×10^{-5}**	42
**29.**	$[Cu(L^{24R})(N_3)]$	**DMF**	**1.344 ×10^{2}**	**4.48×10^{-2}**	**3.73×10^{-6}**	18
**30.**	$[Cu_2(L^{25})(N_3)_3].H_2O$	**DMF**	**3.16×10^{1}**	**1.23×10^{-1}**	**8.8×10^{-7}**	43
**31.**	$[Cu(L^{26})(N_3)]_n$	**DMF**	**9.6×10^{-1}**	**2.58×10^{-3}**	**2.68×10^{-8}**	18
**32.**	$[Cu(L^{14})(N_3)]$	**ACN**	**1.51×10^{-1}**	**2.96×10^{-3}**	**1.26×10^{-7}**	39
**33.**	$[Cu(L^{21})(SCN)]$	**MeOH**	**6.2×10^{6}**	**24.5×10^{-1}**	**1.73×10^{-1}**	41
**34.**	$[Cu(L^{27})(SCN)]$	**MeOH**	**7.8×10^{5}**	**7.5×10^{-1}**	**2.18×10^{-2}**	41

**35.**	[Cu(L^{22R})(SCN)]	**MeOH -DMF**	**3.9×10^{5}**	**7.5×10^{-1}**	**1.1×10^{-2}**	35
**36.**	[Cu(L^{28})(SCN)]	**MeOH**	**2.4×10^{5}**	**1.4×10^{-1}**	**6.69×10^{-3}**	41
**37.**	[Cu(L^{29R})(SCN]	**MeOH -DMF**	**8.2×10^{4}**	**4.7×10^{-1}**	**2.3×10^{-3}**	35
**38.**	[Cu(L^{30R})(SCN)]	**MeOH -DMF**	**6.3×10^{4}**	**3×10^{-2}**	**1.8×10^{-3}**	35
**39.**	[Cu(L^{23})(NCS)(ClO_4)]	**MeOH**	**7.82×10^{1}**	**2.4×10^{-2}**	**2.17×10^{-6}**	28
		**EtOAc**	**4.3×10^{1}**	**1.7×10^{-2}**	**1.91×10^{-6}**	
**40.**	[Cu(L^{26})(NCS)]$_n$	**DMF**	**3.37×10^{1}**	**8.32×10^{-3}**	**9.38×10^{-7}**	18
**41.**	[Cu(L^{31})(NCS)(ClO_4)]	**ACN**	**2.11×10^{1}**	**4.5×10^{-2}**	**5.88×10^{-7}**	28
**42.**	[Cu(L^{14})(NCS)]	**ACN**	**1.42×10^{-1}**	**2.97×10^{-3}**	**1.18×10^{-7}**	39
**43.**	**$Cu_2(\mu_2\text{-}Cl)_2(Cl)_2(L)_2$**	**MeOH**	**$5.5 \pm 0.6 \times10^{3}$**	**10 ± 0.11 *10^{-2}**	**7 ± 0.8 *10^{-5}**	**This Work**

Abb.: MeOH: Methanol, EtOH: Ethanol, NPA: n- Propanol, ACN: Acetonitrile, DMF: Dimethylformamide, H_2O: Water, EtOAc: Ethyl Acetate, DMSO: Dimethyl Sulfoxide. [Note: ^{R} with the ligand denotes it is reduced Schiff Base]

L^{1}: 2-(2-methoxybenzlideneamino)phenol, **L^{2}**: 2-[(1-Hydroxy-2-methylpropan-2-ylimino)methyl]-6-methoxy-4-methylphenol, **L^{3}**: 2-[(3-Hydroxypropylimino)methyl]-6-methoxy-4-methylphenol, **L^{4}**: E)-2-(((2-(Benzylamino)ethyl)amino)methyl)-4-bromophenol (Reduced), **L^{5}**: (E)-2-(((2-(Benzylamino)ethyl)imino)methyl)-4-bromophenol, **L^{6}**: 1-(4-methoxybenzyl)-2-(4-methoxyphenyl)benzimidazole, **L^{7}**: 1-(1H-imidazol-2-yl)-N-(2-(methylthio)phenyl)methanimine, **L^{8}**: 4-methyl-N-(pyridine-2-ylmethyl) aniline, **L^{9}**: 2,4,6-trimethyl-N-(pyridine-2-ylmethyl) aniline, **L^{10}**: *N*-(1-Methylimidazol-2-ylmethyl)quinolin-8-amine, **L^{11}**: *N*-(1-Methylbenzimidazol-2-ylmethyl)quinolin-8-amine, **L^{12}**: *N*-(pyridin-2-ylmethyl)quinolin-8-amine, **L^{13}**: 2-[(2-Hydroxyethylimino)methyl]-6-methoxy-4-methylphenol, **L^{14}**: 2,4-Di-tert-butyl-6-(quinolin-8-yliminomethyl)-phenol, **L^{15}**: 2-[(Phenyl-2-pyridinylmethylene)amino]ethanol, **L^{16}**: 2-Methoxy-6-[[(2-methoxyphenyl)imino]methyl]phenol, **L^{17}**: *N*-[3-[[3-(Dimethylamino)propyl]amino]propyl]imino-2-pyridinealdehyde, **L^{18}**: 2,6-Bis[[[3-[[3-(dimethylamino)propyl]amino]propyl]imino]methyl]-4-methylphenol, **L^{19}**: *N*-[3-[[3-(Dimethylamino)propyl]amino]propyl]imino-2-pyrizine, **L^{20}**: 3-[[2-(Dimethylamino)ethyl]imino]-2-butanone oxime, **L^{21}**: (E)-2-(((2-(Benzylamino)ethyl)imino)methyl)-4-bromophenol, **L^{22}**: (E)-2-(((2-(Benzylamino)ethyl)amino)methyl)-4-bromophenol (Reduced), **L^{23}**: 2-[[(3-Aminopropyl)imino]methyl]-6-methoxyphenol, **L^{24}**: 4-chloro-2-[(2-(propylaminoethylamino)methyl]phenol (Reduced), **L^{25}**: 2-methoxy-6-((2-(2-hydroxyethylamino)ethylimino)methyl)-phenol, **L^{26}**: 4-chloro-2-[(2-(propylaminoethylamino)methyl]phenol, **L^{27}**: (E)-2-(((2-(Benzylamino)ethyl)imino)methyl)phenol, **L^{28}**:

(E)-2-(((2-(Benzylamino)ethyl)imino)methyl)-4-methylphenol, **L[29]:** E)-2-(((2-(Benzylamino)ethyl)amino)methyl)phenol (Reduced), **L[30]:** E)-2-(((2-(Benzylamino)ethyl)amino)methyl)-4-methylphenol (Reduced), **L[31]:** N,N-diethyl-2-((phenyl(pyridine-2-yl)methylene)-amino)ethan-1-amine, **L[32]:** 4-((*E*)-(2-(pyridin-2-yl)ethylimino)methyl)-5-(hydroxymethyl)-2-methylpyridin-3-ol, **L[33]:** 4-methyl-N-(pyridine-2-ylmethyl) aniline, **L[34]**: 2,4,6-trimethyl-N-(pyridine-2-ylmethyl) aniline, **L[35]:** methyl-2-(1-(pyridine-2-yl)ethylidene)hydrazine-1-Carbodithioate, **L[36]:** Benzyl-2-(1-(pyridine-2-yl)ethylidene)hydrazine-1-carbodithioate, **L[37]:** 2-((Pyridine-2-ylethyl)imino)methylphenol, **L[38]:** 2-((Pyridine-2-ylmethyl)imino)methylphenol, **L[39]:** 2-[[bis(2-pyridinylmethyl)amino]methyl]-4-methyl-6-[[[2-(methylthio)phenyl](2 pyridinylmethyl)amino]methyl, **L[40]:**2-[[bis(2-pyridinylmethyl)amino]methyl]-4-methyl-6-[[[2-(methylthio)ethyl](2 pyridinylmethyl)amino]methyl], **L[41]:** 2-[[bis(2-pyridinylmethyl)amino]methyl]-4-methyl-6-[[(2-pyridinylmethyl)(2 thienylmethyl)amino]methyl], **L[42]:** 2,6-bis(4-methyl piperazin-1-yl-methyl)-4-formylPhenol, **L[43]:** N,N′-bis((6-methoxypyridin-2-yl)methyl)-N,N′- dimethylethane-1,2-diamine, **L[44]:** *N*-[3-[[3-(Dimethylamino)propyl]amino]propyl]imino-2-pyridinealdehyde, **L[45]:** 2,6-Bis[[[3-[[3-(dimethylamino)propyl]amino]propyl]imino]methyl]-4-methylphenol, **L[46]:** *N*-[3-[[3 (Dimethylamino)propyl]amino]propyl]imino-2-pyrizine.

References

1. Behera, S.; Dinda, S.; Saha, R.; Mondal, B. Quantitative Electrocatalytic Upcycling of Polyethylene Terephthalate Plastic and Its Oligomer with a Cobalt-Based One-Dimensional Coordination Polymer Having Open Metal Sites along with Coproduction of Hydrogen. *ACS Catalysis* **2022**, *13* (1), 469–474. https://doi.org/10.1021/acscatal.2c05270.
2. Zhou, D.; Li, P.; Lin, X.; McKinley, A.; Kuang, Y.; Liu, W.; Lin, W.-F.; Sun, X.; Duan, X. Layered double hydroxide-based electrocatalysts for the oxygen evolution reaction: identification and tailoring of active sites, and superaerophobic nanoarray electrode assembly. *Chemical Society Reviews* **2021**, *50* (15), 8790–8817. https://doi.org/10.1039/d1cs00186h.
3. Behera, S.; Aziz, S. T.; Singla, N.; Mondal, B. The synergy between electrochemical substrate oxidation and the oxygen reduction reaction to enable aerobic oxidation. *Chemical Communications* **2023**, *59* (77), 11528–11531. https://doi.org/10.1039/d3cc02428h.
4. Gupta, T., Bhavana, R., Ganguly, S., Ghosh, S., & Mondal, B. (2025). Copper phosphide quantum dot: A bifunctional catalyst for electro- and photochemical transformation of biomass-derived 5-

hydroxymethylfurfural. Catalysis Today, 455, 115302. https://doi.org/10.1016/j.cattod.2025.115302

5. Mir, R. A.; Pandey, O. P. Role of morphological features and oxygen vacancies on electrocatalytic oxygen evolution reaction (OER) activity and pseudocapacitance performance of BiVO4 structures. *Applied Physics Letters* **2021**, *118* (25). https://doi.org/10.1063/5.0057082.
6. Machado, R. R. L., Jaato, B. N., Schmidt, T. C., Sanjuán, I., & Andronescu, C. (2025). Quantification of gaseous products evolved at industrially relevant current densities during CO_2 electroreduction and water electrolysis using online gas chromatography. Advanced Energy and Sustainability Research, 7(2). https://doi.org/10.1002/aesr.202500290
7. Koepke, S. J., Light, K. M., VanNatta, P. E., Wiley, K. M., & Kieber-Emmons, M. T. (2017). Electrocatalytic water oxidation by a homogeneous copper catalyst disfavors Single-Site mechanisms. *Journal of the American Chemical Society*, *139*(25), 8586–8600. https://doi.org/10.1021/jacs.7b03278
8. Kumar, N., Wagh, L., Mehmood, S., Das, A. K., & Ghorai, T. K. (2024). Design and development of sustainable Cu2 (II)- and Mn2 (III)-Embedded Bifunctional Electrocatalysts: Enhanced hydrogen and oxygen generation. *Inorganic Chemistry*, *63*(19), 8567–8579. https://doi.org/10.1021/acs.inorgchem.3c04271
9. Zhou, L., Fang, T., Cao, J., Zhu, Z., Su, X., & Zhan, S. (2014). A dinuclear copper(II) electrocatalyst both water reduction and oxidation. *Journal of Power Sources*, *273*, 298–304. https://doi.org/10.1016/j.jpowsour.2014.09.075
10. Zhang, X., Li, Y., Jiang, J., Zhang, R., Liao, R., & Wang, M. (2020). A Dinuclear Copper Complex Featuring a Flexible Linker as Water Oxidation Catalyst with an Activity Far Superior to Its Mononuclear Counterpart. *Inorganic Chemistry*, *59*(8), 5424–5432. https://doi.org/10.1021/acs.inorgchem.9b03783

11. Su, X., Gao, M., Jiao, L., Liao, R., Siegbahn, P. E. M., Cheng, J., & Zhang, M. (2015). Electrocatalytic water oxidation by a dinuclear copper complex in a neutral aqueous solution. *Angewandte Chemie International Edition*, *54*(16), 4909–4914. https://doi.org/10.1002/anie.201411625

12. Chen, Q., Xian, K., Zhang, H., Su, X., Liao, R., & Zhang, M. (2024). Pivotal role of geometry regulation on O−O bond formation mechanism of bimetallic water oxidation catalysts. *Angewandte Chemie International Edition*, *63*(9), e202317514. https://doi.org/10.1002/anie.202317514

13. Fang, T., Fu, L., Zhou, L., & Zhan, S. (2015). A water-soluble dinuclear copper electrocatalyst, [Cu(oxpn)Cu(OH)2] for both water reduction and oxidation. *Electrochimica Acta*, *161*, 388–394. https://doi.org/10.1016/j.electacta.2015.02.101

14. Muthukumar, P., Arunkumar, G., Pannipara, M., Al-Sehemi, A. G., Moon, D., & Anthony, S. P. (2023). Highly enhanced electrocatalytic OER activity of water-coordinated copper complexes: effect of lattice water and bridging ligand. *RSC Advances*, *13*(18), 12065–12071. https://doi.org/10.1039/d3ra01186k

15. Hu, Q., Su, X., & Zhang, M. (2018). Electrocatalytic water oxidation by an unsymmetrical Di-Copper complex. *Inorganic Chemistry*, *57*(17), 10481–10484. https://doi.org/10.1021/acs.inorgchem.8b01173

16. Muley, A.; Karumban, K. S.; Kumbhakar, S.; Mathur, S.; Roy, I.; Verma, A.; Kumawat, M. K.; Maji, S. Tailored design, synthesis, and catalytic aspects of mononuclear cis-dichloro copper(ii) complexes with simple DPA-derived tridentate ligands and their biomimicking activities. *New Journal of Chemistry* **2024**, *48* (17), 7739–7753. https://doi.org/10.1039/d3nj05740b.

17. Mudi, P. K.; Bandopadhyay, N.; Joshi, M.; Shit, M.; Paul, S.; Choudhury, A. R.; Biswas, B. Schiff base triggering synthesis of copper(II) complex and its catalytic fate towards mimics of phenoxazinone synthase activity. *Inorganica Chimica Acta* **2020**, *505*, 119468. https://doi.org/10.1016/j.ica.2020.119468.

18. Mandal, A.; Sarkar, A.; Adhikary, A.; Samanta, D.; Das, D. Structure and synthesis of copper-based Schiff base and reduced Schiff base complexes: a combined experimental and theoretical investigation of biomimetic catalytic activity. *Dalton Transactions* **2020**, *49* (43), 15461–15472. https://doi.org/10.1039/d0dt02784g.

19. Mondal, D.; Ghosh, A. K.; Chatterjee, A.; Ghosh, R. Synthesis and structural characterization of a dinuclear copper(II) complex with a (N,S) donor ligand: Catecholase and phenoxazinone synthase activities. *Inorganica Chimica Acta* **2018**, *486*, 719–723. https://doi.org/10.1016/j.ica.2018.11.046.

20. Adak, P.; Ghosh, B.; Bauzá, A.; Frontera, A.; Blake, A. J.; Corbella, M.; Mukhopadhyay, C. D.; Chattopadhyay, S. K. Catecholase activity, DNA binding and cytotoxicity studies of a Cu(ii) complex of a pyridoxal schiff base: synthesis, X-ray crystal structure, spectroscopic, electrochemical and theoretical studies. *RSC Advances* **2016**, *6* (90), 86851–86861. https://doi.org/10.1039/c6ra14059a.

21. Mandal, A.; Dasgupta, S.; Ganguly, S.; Bauzá, A.; Frontera, A.; Das, D. Cooperative influence of pseudohalides and ligand backbone of Schiff-bases on nuclearity and stereochemistry of cobalt(iii) complexes: experimental and theoretical investigation. *Dalton Transactions* **2017**, *46* (44), 15257–15268. https://doi.org/10.1039/c7dt03040a.

22. Mukherjee, D.; Nag, P.; Shteinman, A. A.; Vennapusa, S. R.; Mandal, U.; Mitra, M. Catechol oxidation promoted by bridging phenoxo moieties in a bis(μ-phenoxo)-bridged dicopper(ii) complex. *RSC Advances* **2021**, *11* (37), 22951–22959. https://doi.org/10.1039/d1ra02787e.

23. Chakraborty, M.; Mondal, A.; Chattopadhyay, S. K. Structural divergence in binuclear Cu(ii) pyridoxal Schiff base complexes probed by co-ligands: catecholase mimetic activity and sulphide ion sensing. *New Journal of Chemistry* **2020**, *44* (30), 12916–12925. https://doi.org/10.1039/d0nj00719f.

24. Santra, A.; Brandao, P.; Mondal, G.; Bera, P.; Jana, A.; Bhattacharyya, I.; Pramanik, C.; Bera, P. The role of methyl and benzyl substituted dithiocarbazate of 2-acetyl pyridine for the formation of

bridged dimeric and unbridged monomeric copper(II) complexes and catecholase mimetic activity of the complexes. *Polyhedron* **2019**, *176*, 114277. https://doi.org/10.1016/j.poly.2019.114277.

25. Ravisankar, V. C.; Selvakumaran, B.; Ajaykamal, T.; Murali, M. Functional biomimetics for copper oxidases: interesting catalytic promiscuity of novel monocopper(ii) complexes. *Dalton Transactions* **2025**, *54* (18), 7221–7239. https://doi.org/10.1039/d5dt00077g.
26. Durigon, D. C.; Glitz, V. A.; Pimenta, B. F.; Guedes, A. M. V.; Silva, J. V. O.; Cruz, C. C. B.; Andrade, L. M.; Pereira-Maia, E. C.; Mikcha, J. M. G.; Cruz, A. B.; Xavier, F. R.; Terenzi, H. F.; Poneti, G.; Ribeiro, R. R.; Nordlander, E.; Caramori, G. F.; Bortoluzzi, A. J.; Peralta, R. A. The influence of thioether-substituted ligands in dicopper(II) complexes: Enhancing oxidation and biological activities. *Journal of Inorganic Biochemistry* **2024**, *256*, 112573. https://doi.org/10.1016/j.jinorgbio.2024.112573.
27. Mandal, S.; Naskar, R.; Mukherjee, R.; Mondal, A. S.; Das, A.; Murmu, N.; Mondal, T. K. Synthesis of a new benzoate bridged NNO tethered copper(ii) complex: exploration of its bio, catalytic and anticancer activities. *New Journal of Chemistry* **2023**, *47* (32), 15267–15282. https://doi.org/10.1039/d3nj01806g.
28. Chowdhury, I. R.; Chatterjee, A.; Mahanta, A.; Jasimuddin, S.; Ghosh, R. Catecholase and phenoxazinone synthase activities of two structurally characterized mononuclear complexes [Cu(L)(NCS)(ClO4)] and [Zn(L)(NCS)2] [L = N,N-diethyl-2-((phenyl(pyridin-2-yl)methylene)-amino)ethan-1-amine]. *Inorganica Chimica Acta* **2024**, *566*, 122006. https://doi.org/10.1016/j.ica.2024.122006.
29. Sýs, M.; Kocábová, J.; Klikarová, J.; Novák, M.; Jirásko, R.; Obluková, M.; Mikysek, T.; Sokolová, R. Comparison of mononuclear and dinuclear copper(ii) biomimetic complexes: spectroelectrochemical mechanistic study of their catalytic pathways. *Dalton Transactions* **2022**, *51* (36), 13703–13715. https://doi.org/10.1039/d2dt01610a.
30. Jana, N. Ch.; Sun, Y.-C.; Herchel, R.; Nandy, R.; Brandão, P.; Bagh, B.; Wang, X.-Y.; Panja, A. Chemical fixation of atmospheric CO2 in tricopper(ii)-carbonato complexes with tetradentate N-

donor ligands: reactive intermediates, probable mechanisms, and catalytic and magneto-structural studies. *Dalton Transactions* **2024**, *53* (27), 11514–11530. https://doi.org/10.1039/d4dt00503a.

31. Jana, N. Ch.; Ghorai, P.; Brandão, P.; Jagličić, Z.; Panja, A. Proton controlled synthesis of two dicopper(ii) complexes and their magnetic and biomimetic catalytic studies together with probing the binding mode of the substrate to the metal center. *Dalton Transactions* **2021**, *50* (42), 15233–15247. https://doi.org/10.1039/d1dt02369a.
32. Dutta, T.; Mirdya, S.; Giri, P.; Chattopadhyay, S. Synthesis and characterization of a double oximato bridged dimeric copper(II) complex and its use in oxidative dimerisation of o-aminophenol. *Polyhedron* **2019**, *175*, 114164. https://doi.org/10.1016/j.poly.2019.114164
33. Mahato, S.; Meheta, N.; Kotakonda, M.; Joshi, M.; Ghosh, P.; Shit, M.; Choudhury, A. R.; Biswas, B. Ligand directed synthesis of a unprecedented tetragonalbipyramidal copper (II) complex and its antibacterial activity and catalytic role in oxidative dimerisation of 2-aminophenol. *Applied Organometallic Chemistry* **2020**, *34* (11). https://doi.org/10.1002/aoc.5935.
34. Jana, N. Ch.; Escuer, A.; Brandão, P.; Panja, A. Exploring structural, magnetic, and catalytic diversity in tetranuclear {Cu4O4} cubane cores and a dinuclear complex derived from closely related ligand systems. *Dalton Transactions* **2025**, *54* (7), 3000–3012. https://doi.org/10.1039/d4dt03283g.
35. Mohammed, T. P.; George, A.; Sivaramakrishnan, M. P.; Vadivelu, P.; Balasubramanian, S.; Sankaralingam, M. Deciphering the effect of amine versus imine ligands of copper(II) complexes in 2-aminophenol oxidation. *Journal of Inorganic Biochemistry* **2023**, *247*, 112309. https://doi.org/10.1016/j.jinorgbio.2023.112309.
36. Mudi, P. K.; Mahato, R. K.; Joshi, M.; Shit, M.; Choudhury, A. R.; Das, H. S.; Biswas, B. Copper(II) complexes with a benzimidazole functionalized Schiff base: Synthesis, crystal structures, and role of ancillary ions in phenoxazinone synthase activity. *Applied Organometallic Chemistry* **2021**, *35* (6). https://doi.org/10.1002/aoc.6211.

37. Bandopadhyay, N.; Paramanik, K.; Mudi, P. K.; Sarkar, G.; Kotakonda, M.; Shit, M.; Biswas, B.; Das, H. S. A thiomethyl-substituted imidazolyl imine functionalized copper(II) complex: synthesis, structural characterization, phenoxazinone synthase mimics and biological activities. *Polyhedron* **2022**, *218*, 115783. https://doi.org/10.1016/j.poly.2022.115783.

38. Podder, N.; Mandal, S. Aerobic oxidation of 2-aminophenol catalysed by a series of mononuclear copper(ii) complexes: phenoxazinone synthase-like activity and mechanistic study. *New Journal of Chemistry* **2020**, *44* (29), 12793–12805. https://doi.org/10.1039/d0nj02558e.

39. Mandal, S.; Naskar, R.; Mondal, A. S.; Bera, B.; Mondal, T. K. Facile synthesis of novel NNO-tethered copper(ii) complexes: characterization details, theoretical studies, promising enzyme-like activities, and biomolecular interactions. *Dalton Transactions* **2023**, *52* (18), 5983–5998. https://doi.org/10.1039/d2dt04170g.

40. Chatterjee, A.; Khan, S.; Ghosh, R. Structurally characterized mononuclear isostructural Ni(II), Cu(II) and Zn(II) complexes as a functional model for phenoxazinone synthase activity. *Polyhedron* **2019**, *173*, 114151. https://doi.org/10.1016/j.poly.2019.114151.

41. Thennarasu, A. S.; Mohammed, T. P.; Sankaralingam, M. Mononuclear copper(ii) Schiff base complexes as effective models for phenoxazinone synthase. *New Journal of Chemistry* **2022**, *46* (45), 21684–21694. https://doi.org/10.1039/d2nj03934f.

42. Roy, S.; Dutta, T.; Drew, M. G. B.; Chattopadhyay, S. Phenoxazinone synthase mimicking activity of a dinuclear copper(II) complex with a half salen type Schiff base ligand. *Polyhedron* **2019**, *178*, 114311. https://doi.org/10.1016/j.poly.2019.114311.

43. Mirdarvatan, V.; Bahramian, B.; Khalaji, A. D.; Vaclavu, T.; Gómez-García, C. J.; Benmansour, S.; Triki, S. A novel mixed azido/phenoxido bridged 1D CuII coordination polymer containing o-vanillin-based compartmental ligand: Synthesis, magnetism, phenoxazinone synthase and catalytic activity in epoxidation of alkenes. *Polyhedron* **2023**, *244*, 116581. https://doi.org/10.1016/j.poly.2023.116581.
